# Energy status-promoted growth and development of Arabidopsis require copper deficiency response transcriptional regulator SPL7

**DOI:** 10.1093/plcell/koac215

**Published:** 2022-07-22

**Authors:** Anna Schulten, Björn Pietzenuk, Julia Quintana, Marleen Scholle, Regina Feil, Marcus Krause, Maida Romera-Branchat, Vanessa Wahl, Edouard Severing, George Coupland, Ute Krämer

**Affiliations:** Department of Molecular Genetics and Physiology of Plants, Ruhr University Bochum, 44801 Bochum, Germany; Department of Molecular Genetics and Physiology of Plants, Ruhr University Bochum, 44801 Bochum, Germany; Department of Molecular Genetics and Physiology of Plants, Ruhr University Bochum, 44801 Bochum, Germany; Department of Molecular Genetics and Physiology of Plants, Ruhr University Bochum, 44801 Bochum, Germany; Max Planck Institute of Molecular Plant Physiology, 14476 Potsdam-Golm, Germany; Department of Molecular Genetics and Physiology of Plants, Ruhr University Bochum, 44801 Bochum, Germany; Max Planck Institute for Plant Breeding Research, 50829 Cologne, Germany; Max Planck Institute of Molecular Plant Physiology, 14476 Potsdam-Golm, Germany; Max Planck Institute for Plant Breeding Research, 50829 Cologne, Germany; Max Planck Institute for Plant Breeding Research, 50829 Cologne, Germany; Department of Molecular Genetics and Physiology of Plants, Ruhr University Bochum, 44801 Bochum, Germany

## Abstract

Copper (Cu) is a cofactor of around 300 Arabidopsis proteins, including photosynthetic and mitochondrial electron transfer chain enzymes critical for adenosine triphosphate (ATP) production and carbon fixation. Plant acclimation to Cu deficiency requires the transcription factor SQUAMOSA PROMOTER-BINDING PROTEIN-LIKE7 (SPL7). We report that in the wild type (WT) and in the *spl7-1* mutant, respiratory electron flux via Cu-dependent cytochrome *c* oxidase is unaffected under both normal and low-Cu cultivation conditions. Supplementing Cu-deficient medium with exogenous sugar stimulated growth of the WT, but not of *spl7* mutants. Instead, these mutants accumulated carbohydrates, including the signaling sugar trehalose 6-phosphate, as well as ATP and NADH, even under normal Cu supply and without sugar supplementation. Delayed *spl7-1* development was in agreement with its attenuated sugar responsiveness. Functional TARGET OF RAPAMYCIN and SNF1-RELATED KINASE1 signaling in *spl7*-*1* argued against fundamental defects in these energy-signaling hubs. Sequencing of chromatin immunoprecipitates combined with transcriptome profiling identified direct targets of SPL7-mediated positive regulation, including *Fe SUPEROXIDE DISMUTASE1* (*FSD1*), *COPPER-DEFICIENCY-INDUCED TRANSCRIPTION FACTOR1* (*CITF1*), and the uncharacterized *bHLH23* (*CITF2*), as well as an enriched upstream GTACTRC motif. In summary, transducing energy availability into growth and reproductive development requires the function of SPL7. Our results could help increase crop yields, especially on Cu-deficient soils.

## Teaser

Arabidopsis requires the transcription factor SQUAMOSA PROMOTER-BINDING PROTEIN-LIKE7 (SPL7), a known regulator of copper deficiency responses, for transducing sugar levels into growth and development.

IN A NUTSHELL **Background:** To accomplish a multitude of biochemical reactions, life takes advantage of the rich chemical properties of metals present on Earth — among these copper (Cu) — in the form of protein cofactors. Of the approximately 300 Cu-requiring proteins encoded in the genome of the model plant Arabidopsis, two proteins mediate decisive electron transfer reactions in photosynthesis and respiration, which are central processes in plant energy metabolism. Available Cu levels in soil are often insufficient. In response, plants activate root Cu uptake, and reorganize their metabolism to consume less Cu, for example by replacing some Cu-dependent proteins by Cu-independent functional equivalents. The transcription factor SQUAMOSA PROMOTER-BINDING PROTEIN-LIKE7 (SPL7) mediates the required changes in gene regulation that accompany this transition.

**Question:** Given the key roles of Cu in plant energy metabolism, we asked whether SPL7 has additional functions in integrating Cu handling with metabolism and development in Arabidopsis. Furthermore, we addressed which genes that may contribute to such additional functions undergo direct transcriptional regulation by SPL7.

**Findings:** We discovered that *spl7* mutants fail to utilize sugars and instead accumulate them. Despite their high energy status, *spl7* mutants grow poorly. We discovered that critical Cu-dependent functions in photosynthesis and respiration remain intact in the mutants, suggesting that *spl7* mutants might be impaired in sensing their high energy status or in transducing it into the appropriate growth responses. Yet, the best-studied central sugar-signaling pathways responded normally in an *spl7* mutant. Consequently, to understand how SPL7 enables plants to utilize sugar, we globally identified the genes whose promoters SPL7 binds directly to and that undergo SPL7-dependent regulation in Arabidopsis seedlings.

**Next steps:** Now that this information is available, the next step is to test several of the identified SPL7 target genes and pathways for their involvement in SPL7-dependent sugar utilization. An analysis of global SPL7-dependent responses to endogenous sugar levels will also be informative.

## Introduction

Land plants depend on acquiring mineral nutrients from the soil solution through their roots. The functions of numerous metalloproteins require mineral nutrients of the class B and borderline elements (Nieboer and Richardson, 1980), for example, iron (Fe), zinc (Zn), and copper (Cu), referred to as “transition metals” or simply “metals”. A plant metal homeostasis network operates to fulfill the demands of the metalloproteomes of different organs, tissues, and cell types, as well as to counteract the local accumulation of a toxic excess of any essential or chemically similar nonessential metal (Clemens, 2001; Krämer and Clemens, 2005). Many of the network components mediating metal acquisition, distribution, utilization, and storage in plants have been functionally characterized. Notably, we know much less about the upstream regulatory components, for example metal sensors, signal transduction pathways, and proteins activating acclimation responses. Among the latter, several identified transcription factors mediate transcriptional responses to Fe, Zn, or Cu deficiency in Arabidopsis (Colangelo and Guerinot, 2004; Wang et al., 2007; Yamasaki et al., 2009; Assunção et al., 2010; Bernal et al., 2012; Li et al., 2016; Liang et al., 2017; Yan et al., 2017).

Of all essential metals, Cu^+^ and Cu^2+^ cations possess the highest ligand-binding affinities, and Cu readily catalyzes single-electron transfer reactions involving the Cu^2+^/Cu^+^ redox couple (Fraústo da Silva and Williams, 2001). Probably as a result of these potent chemical properties, cellular Cu homeostasis is exceptionally tight (Rae et al., 1999; Robinson and Winge, 2010; Foster et al., 2014). In plants, the largest Cu quota are required in chloroplasts, where Cu acts as a cofactor of PLASTOCYANIN (PC), the soluble electron carrier between the cytochrome *b_6_f* complex and photosystem I in photosynthetic electron transfer and one of the most abundant proteins in the thylakoid lumen (Redinbo et al., 1994; Schubert et al., 2002; Shikanai et al., 2003; Weigel et al., 2003; Ravet et al., 2011; Yruela, 2013). The association of PC with its Cu cofactor occurs posttranslationally in the thylakoid lumen and involves the Cu-transporting P-TYPE ATPASE OF ARABIDOPSIS1 (PAA1) in the inner chloroplast envelope membrane and PAA2 in the thylakoid membrane (Shikanai et al., 2003; Abdel-Ghany et al., 2005; Blaby-Haas et al., 2014). In mitochondria, the second most relevant cellular site of Cu use, Cu acts as a cofactor in cytochrome *c* oxidase (COX or mitochondrial complex IV), which transfers electrons to oxygen as the terminal electron acceptor of the respiratory mitochondrial electron transport chain (mETC) (Kadenbach et al., 2000). Total respiration dropped by ∼60% in Cu-deficient cells of the green alga Chlamydomonas (*Chlamydomonas reinhardtii*) compared to Cu-replete cells (Kropat et al., 2015). The presence of Cu-dependent enzymes at the two key cellular sites of adenosine triphosphate (ATP) production emphasizes the central relevance of Cu in plant energy metabolism. Whether plants integrate Cu homeostasis and energy metabolism, however, remains unknown.

The transcription factor SQUAMOSA PROMOTER-BINDING PROTEIN-LIKE7 (SPL7) operates as a regulator of Cu deficiency-responsive gene expression in Arabidopsis (*Arabidopsis thaliana*) (Yamasaki et al., 2009; Bernal et al., 2012). SPL7 is a member of a transcription factor family characterized by the Squamosa promoter-Binding Protein (SBP) domain, which contains both a nuclear localization signal and a recognition domain for binding to a GTAC core DNA motif (Cardon et al., 1999; Birkenbihl et al., 2005). The Arabidopsis SPL family comprises 16 proteins grouped in subfamily I (SPL1, 7, 12, 14, and 16) and subfamily II, based on size and sequence similarity (Xing et al., 2010). Subfamily II SPL proteins (SPL2–6, SPL8–11, SPL13, and SPL15) have regulatory roles in diverse aspects of plant development, including leaf and trichome formation, developmental phase transitions, floral meristem identity, and fertility (Unte et al., 2003; Wu and Poethig, 2006; Wang et al., 2008; Yamaguchi et al., 2009; Xing et al., 2010, 2013; Yu et al., 2010; Xu et al., 2016; He et al., 2018). With plant age, a gradual decrease in the abundance of microRNA (miRNA) 156, which targets transcripts encoding all subfamily II SPL proteins except SPL8, causes an increasing abundance of these SPL proteins and thus of their cellular activities, thereby promoting the vegetative-to-reproductive phase change (Wu et al., 2009; Yang et al., 2011, 2013; Yu et al., 2013). With the exception of SPL7, the biological functions of subfamily I SPL proteins are less well understood (Stone et al., 2005; Chao et al., 2017; Schulten et al., 2019).

SPL7 is required to enhance the transcription of genes encoding proteins with roles in Cu acquisition, such as the root surface Cu(II) chelate reductases (annotated as FERRIC REDUCTASE OXIDASE [FRO]) FRO4/FRO5, and several COPPER TRANSPORTER (COPT) family membrane proteins which transport Cu^+^ into the cytosol (Bernal et al., 2012). Additionally, SPL7 mediates the miRNA-dependent posttranscriptional downregulation of the levels of transcripts encoding several abundant but nonessential Cu metalloproteins. This aspect of the Arabidopsis Cu-deficiency response, which resembles Cu sparing in the green alga Chlamydomonas, is thought to reflect an economization strategy that prioritizes the allocation of Cu to essential cuproproteins like PC (Quinn and Merchant, 1995; Kropat et al., 2005; Abdel-Ghany and Pilon, 2008; Yamasaki et al., 2009). For example, under Cu-deficiency conditions, the transcripts encoding the abundant Cu/Zn SUPEROXIDE DISMUTASES1 and 2, *CSD1* and *CSD2*, are targeted by miR398 to replace these CSDs by Fe SUPEROXIDE DISMUTASE1 (FSD1) in an SPL7-dependent manner (Yamasaki et al., 2007, 2009; Abdel-Ghany and Pilon, 2008). Chromatin immunoprecipitation followed by deep sequencing (ChIP-seq) using transgenic Arabidopsis lines expressing a construct encoding FLAG-tagged SPL7 under the control of the cauliflower mosaic virus (CaMV) 35S promoter identified 1,266 genes associated with SPL7-binding sites under Cu deficiency (Zhang et al., 2014), far more than the 188 transcripts identified as being upregulated or downregulated in an *SPL7*-dependent manner (Bernal et al., 2012). COPPER-DEFICIENCY-INDUCED TRANSCRIPTION FACTOR1 (CITF1) was identified through its physical interaction with SPL7 (Yan et al., 2017). The regulation of *CITF1* transcript levels is largely dependent on *SPL7*, and *CITF1* contributes to Cu-deficiency responses of roots and reproductive organs, as well as to pollen fertility and jasmonate responses in flowers (Yan et al., 2017).

Here we tested the hypothesis of an integration between Cu homeostasis and plant energy metabolism through SPL7. Arabidopsis *spl7* mutants, which lack a broad range of Cu-deficiency responses, were unresponsive to growth stimulation by exogenous sucrose under Cu deficiency, contrasting with a strong growth-enhancing effect of sucrose in the wild type (WT). An accumulation of sugars including the signaling sugar trehalose 6-phosphate (T6P) suggested that *spl7* mutants are impaired in sugar utilization upon cultivation in both Cu-deficient and Cu-sufficient media. Normal or even elevated levels of ATP and respiratory electron flux via COX, as well as NADH (reduced nicotinamide adenine dinucleotide), argue against a simple biochemical defect in *spl7-1*. These metabolic features clearly differentiated *spl7* mutants from Cu-deficient WT plants, and they were also unlike the metabolic features previously reported in severely Cu-limited WT plants (Rahmati Ishka and Vatamaniuk, 2020). The activities of the kinases TARGET OF RAPAMYCIN (TOR) and SNF1-RELATED KINASE1 (SnRK1) were sugar responsive and in agreement with elevated sugar levels in *spl7-1*, suggesting that these major energy signaling pathways are generally functional in the mutant. When grown under long-day (LD) conditions, the *spl7*-*1* mutant flowered after a substantially longer growth period than the WT, irrespective of soil Cu levels. We sequenced chromatin immunoprecipitates from Arabidopsis *spl7-1* lines expressing a construct encoding HA-tagged SPL7 under the control of the *SPL7* promoter and terminator, and we also conducted a comparative transcriptome deep sequencing (RNA-seq) analysis in *spl7*-*1* and the WT. Together, these data suggest a direct activation of the transcription of genes encoding basic helix–loop–helix (bHLH) transcription factors by SPL7 and delineate candidate processes and genes for roles in *SPL7*-dependent sugar signaling. We report a predominantly promoter-localized GTACTRC motif as being enriched among SPL7-bound genes that show *SPL7*-dependent increases in transcript abundance under Cu deficiency, while other enriched motifs are likely to reflect additional and complex roles of SPL7. In summary, maintaining a balance between energy availability, growth, and development requires SPL7 function, especially under Cu-deficient growth conditions.

## Results

### The *spl7*-*1* mutant is impaired in directing sugars into growth processes and accumulates sugars

**Figure 1:**
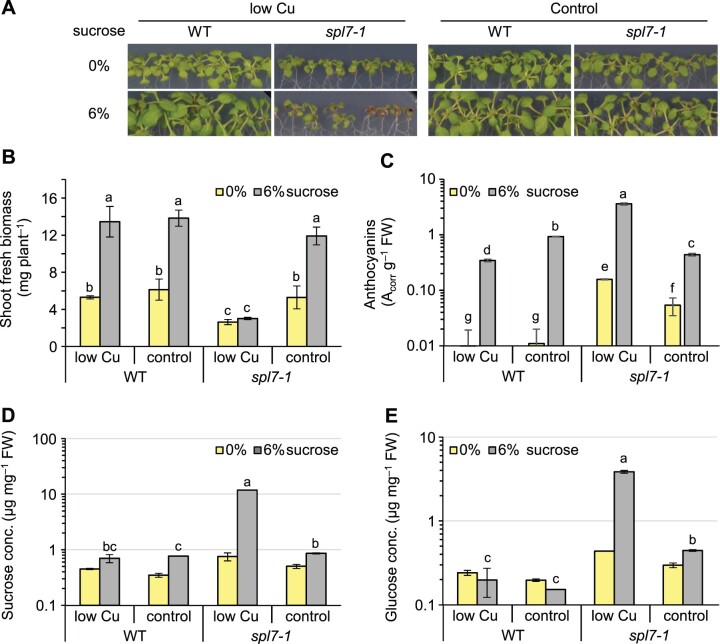
The *spl7-1* mutant accumulates elevated levels of sugars instead of directing them into growth. A, Photographs of 21-day-old WT and *spl7-1* mutant seedlings cultivated for 14 days in vertically oriented glass petri plates on low-Cu (0.05 µM CuSO_4_) or control (0.5 µM CuSO_4_) EDTA-washed agar medium with no sucrose (0%) or supplemented with 6% (w/v) sucrose. B, Fresh shoot biomass of seedlings in (A). Data are means ± sd (*n* = 3 pools of seedlings, with each pool from one replicate plate). C, Anthocyanin levels of shoots in (A). Data are means ± sd (*n* = 3 technical replicates). D and E, Sucrose (D) and glucose (E) concentrations of shoots in (A). Data are means ± sd (*n* = 2 and 3 technical replicates for 0% and 6% sucrose samples, respectively). Technical replicates (C–E) are replicate measurements conducted per extract from a pool of 20 or 40 shoots of seedlings cultivated on one plate per genotype and treatment; see “Materials and Methods”). Data from independent experiments (independently grown sets of plants) are shown in Supplemental Figure S3, A–C. Different lowercase letters denote significant differences between means based on *t* tests with FDR adjustment, *q*-value < 0.05, B–E, wherever *n* > 2). FW: fresh biomass; n.d.: not detectable.

To test for interactions between Cu homeostasis and sugar metabolism, we transferred 7-day-old WT and *spl7-1* mutant seedlings onto solid agar medium differing in Cu and sucrose contents. After 14 days, we quantified the concentrations of Cu, Fe, Zn, and magnesium (Mg) in shoots (Supplemental Figure S1), which confirmed the specific establishment of Cu deficiency. Concentrations of Cu were in agreement with our expectation of generally lowered Cu levels in *spl7-1* (Bernal et al., 2012; Marschner and Marschner, 2012). In the WT, we observed a sucrose-dependent stimulation of biomass production by up to 60% irrespective of Cu supply, but this sugar response was absent in *spl7-1* cultivated on low Cu (Figure 1, A and B). In addition, the biomass of *spl7*-*1* and *spl7-2* did not increase significantly when grown on moderate sucrose concentrations between 0.1% (w/v) and 3% (w/v) in low-Cu medium, in contrast to the WT and an *spl7-2 SPL7* complemented line (Supplemental Figure S2) (Bernal et al., 2012).

Anthocyanin production is a well-known plant response to high internal levels of sucrose (Larronde et al., 1998; Weiss, 2000; Teng et al., 2005; Solfanelli et al., 2006). The *spl7*-*1* and *spl7-2* mutants both accumulated higher anthocyanin levels than the WT and a complemented line (Bernal et al., 2012), with the highest anthocyanin concentrations measured for *spl7* mutants cultivated under low-Cu conditions (Figure 1, A and C; Supplemental Figure S3A). RESPONSIVE-TO-ANTAGONIST1 (RAN1, also named HEAVY METAL ATPASE7 [HMA7]) is a Cu-transporting ATPase required for the incorporation of the Cu^+^ cofactor in the ethylene receptor (Rodriguez et al., 1999), and the partial loss-of-function mutant *ran1-1* exhibits a mild constitutive ethylene response. We would expect a trend toward a constitutive ethylene response in *spl7* mutants due to their Cu acquisition and allocation defects, similar to *ran1-1*. The WT-like anthocyanin levels in *ran1-1* even under low-Cu high-sucrose conditions argued against the possibility of a secondary ethylene signaling defect as a cause of the anthocyanin accumulation in *spl7* mutants. Indeed, mutations suppressing ethylene responses result in enhanced sugar-inducible anthocyanin accumulation, in contrast to mutations that constitutively activate ethylene responses, such as *constitutive triple response 1-1* (*ctr1-1*), *ran1-1* (Jeong et al., 2010), and *spl7*.

The shoots of *spl7*-*1* cultivated in low Cu accumulated about two-fold higher sugar levels than WT shoots without exogenous sucrose supply and 17- and 20-fold higher sucrose and glucose levels under high-sucrose cultivation conditions (Figure 1, D and E; see Supplemental Figure S3, B and C for an independent experiment). Notably, sugar levels were elevated in the *spl7-1* mutant even under Cu-sufficient control conditions, despite a fresh biomass comparable to that of the WT (Figure 1, A, B, D and E; Supplemental Figure S3, B and C).

We also observed *SPL7*-independent ionomic alterations in seedlings grown on high-sucrose-containing medium, namely generally decreased shoot Fe and Cu levels under low-Cu conditions (Supplemental Figure S1, A and B, compare with S1, C–F). Changes in transcript levels were reported for SPL7-regulated genes in response to sucrose in WT plants cultivated on MS (Murashige and Skoog, 1962) medium containing 6% (w/v) sucrose (Dugas and Bartel, 2008; Ren and Tang, 2012). We confirmed a sucrose-dependent upregulation of *pri-MIR398B* transcripts, accompanied by a downregulation of transcript levels for the miR398 target *CSD2*, albeit only under low-Cu conditions, as well as a downregulation of *FSD1* and *COPT2* transcript levels (Supplemental Figure S4). Importantly, these sucrose responses of transcript levels were generally of a low magnitude and remained detectable in the *spl7-1* mutant.

Pursuing our focus on SPL7-dependent phenotypes in this study, we wished to identify the causes of the defects in sugar utilization in *spl7-1*. Fixed carbon accumulates as starch during the day and is remobilized during the night to avoid carbon depletion and allow continued growth. Accordingly, reduced growth rates have been observed under short-day (SD) conditions (8-h light/16-h dark) in several mutants exhibiting defects in starch biosynthesis or degradation (e.g. *phosphoglucomutase*) (Caspar et al., 1985; Gibon et al., 2004). At the end of the day, starch concentrations in the *spl7*-*1* mutant paralleled sugar levels, and they were similar or higher (no sucrose) or far higher (6% sucrose) than in the WT, demonstrating that the starch biosynthesis pathways are generally functional in the mutant (Supplemental Figure S5A). At the end of the night, starch was depleted or strongly reduced throughout all seedlings, except in *spl7* mutants cultivated under combined low-Cu and high-sucrose conditions (Supplemental Figure S5, B and C). We interpret this result as a consequence of the very high availability of soluble sugars (see Figure 1, D and E; Supplemental Figure S3, B and C) and starch in *spl7* mutants under this condition. We conclude that both the biosynthesis and mobilization of starch are generally functional in *spl7-1*.

Taken together, these results suggest that *spl7* mutants are generally impaired in the utilization of sugars and that low-Cu growth conditions exacerbate this metabolic phenotype further. This phenotype may reflect a direct function of SPL7, or it may arise indirectly in *spl7* mutants as a symptom from the loss of Cu-dependent biochemical functions normally required for sugar utilization, given the Cu homeostasis defects of *spl7* mutants and their apparent susceptibility to physiological Cu limitation.

### Increased respiration rates and altered profile of respiration-related metabolites in *spl7*-*1*

**Figure 2:**
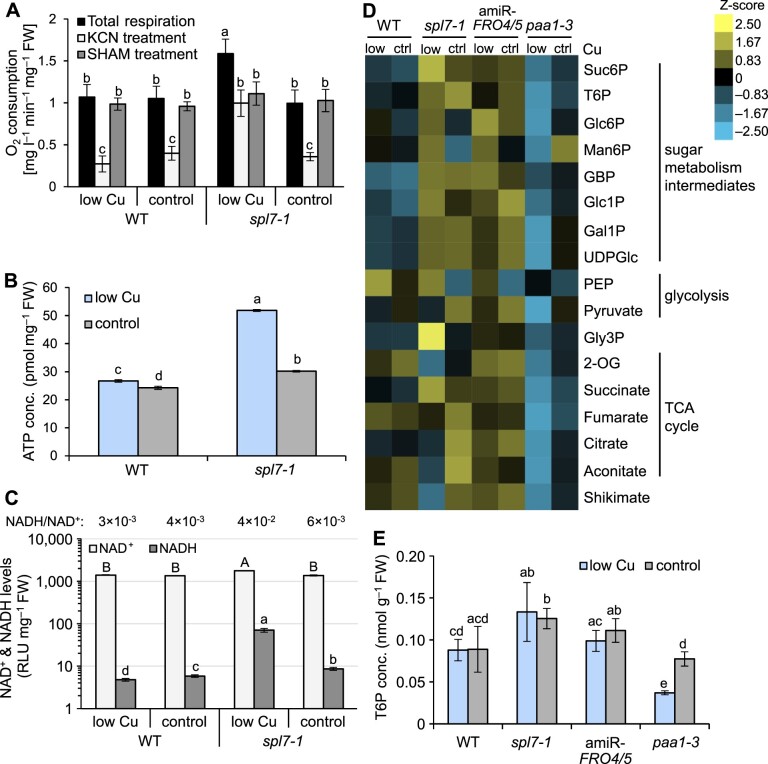
Respiratory activity and quantification of metabolites associated with sugar metabolism and respiration. A, Respiration rates, measured as O_2_ consumption in darkness. Data are from leaves of 21-day-old WT and *spl7-1* seedlings cultivated in vertically oriented glass petri plates on low-Cu (no CuSO_4_ added) or control (0.5 µM CuSO_4_) EDTA-washed agar solid medium supplemented with 1% (w/v) sucrose. The Complex IV inhibitor KCN (1 mM) or AOX inhibitor SHAM (20 mM) was present during a subset of respiration measurements, as indicated. Data are means ± sd (*n* = 8 and 4 replicate seedling batches for total respiration and for inhibitor treatments, respectively). B and C, ATP (B), NAD^+^ and NADH levels (C) in the shoots of seedlings cultivated as described for (A). Data are means ± sd (*n* = 3 technical replicates). D, Heatmap representation of metabolite levels of respiratory and sugar metabolism. Data are from shoots of 21-day-old seedlings (WT, *spl7-1*, amiR*-FRO4/5*, *paa1-3*) cultivated in vertically oriented glass petri plates on low-Cu (0.05 µM CuSO_4_) or control (0.5 µM CuSO_4_) EDTA-washed agar solid medium with no added sucrose for 14 days. Represented are *z*-scores (*n* = 4 replicate pools per genotype, with one pool per replicate petri plate). E, T6P concentrations in shoots of seedlings cultivated as described for (D). Data are means ± sd (*n* = 4, see (D)). Compare to Figure 5A. Different lowercase letters denote statistically significant differences (*P* < 0.05) between means based on ANOVA (followed by Tukey’s honestly significant test [HSD]) (A, B) or *t* tests with FDR adjustment (*q* < 0.05) for (C and E), with uppercase and lowercase letters corresponding to different test groups. Data for a second (B and C) and two additional independent experiments (D) are shown in Supplemental Figure S6, A–D. RLU: relative light units; Suc6P: sucrose 6-phosphate; T6P: trehalose 6-phosphate; Glc6P: glucose 6-phosphate; Man6P: mannose 6-phosphate; GBP: glucose 1,6-bisphosphate; Glc1P: glucose 1-phosphate; Gly3P: glycerol 3-phosphate; 2-OG: 2-oxoglutarate; Gal1P: galactose 1-phosphate; UDPGlc: uridine diphosphate glucose; PEP: phosphoenolpyruvate.

Mitochondrial respiration connects the breakdown of carbohydrates to the production of ATP and carbon skeletons as biosynthetic precursors (O'Leary et al., 2019) (Supplemental Figure S6A). We tested whether defective Cu homeostasis in the *spl7*-*1* mutant results in respiratory restrictions, which might occur through a decrease in Cu-dependent COX activity and feed back to cause insufficient sugar catabolism (Dahan et al., 2014). Notably, total respiration rate was not decreased in *spl7*-*1* and was even significantly higher under low Cu in the mutant compared to the WT (Figure 2A). In the presence of the inhibitor of mitochondrial ALTERNATIVE OXIDASE (AOX) salicylhydroxamic acid (SHAM), respiration rates did not differ between the *spl7-1* mutant and the WT (Figure 2A). When AOX activity is inhibited by SHAM, electrons are not redirected into the COX pathway (Bahr and Bonner, 1973; Møller et al., 1988). Consequently, our results suggest that COX (mitochondrial complex IV) is fully functional in *spl7*-*1* even when grown on low-Cu medium.

In the presence of potassium cyanide (KCN), electron flux through the cytochrome *c* pathway is inhibited and consequently redirected to the alternative pathway according to the maximum AOX capacity (Møller et al., 1988). The respiration rates of *spl7*-*1* seedlings grown on low-Cu medium remained more than twice as high as those of the WT under KCN treatment, indicating a much higher capacity for KCN-insensitive respiration in *spl7*-*1* (Figure 2A). Accordingly, elevated levels of total respiration in *spl7*-*1* cultivated under low-Cu conditions can likely be attributed to a higher AOX activity, in agreement with increased transcript levels of all four genes encoding AOX isoforms in shoots of the *spl7*-2 mutant, particularly of the isoform *AOX1D* (Bernal et al., 2012).

ATP levels were about 1.3-fold higher in the *spl7-1* mutant under control conditions and two-fold higher under low-Cu conditions, by comparison to the WT (Figure 2B; Supplemental Figure S6B). The NADH/NAD^+^ ratio of *spl7-1* was between ∼1.5-fold (control Cu) and nine-fold (low Cu) higher than that of the WT (Figure 2C; Supplemental Figure S6C). These ratios are generally consistent with previous findings that NADH contributes less than ∼1% of the cytosolic NAD pool in WT plants (Heineke et al., 1991; Shen et al., 2006). Compared to the WT, the *spl7*-*1* mutant contained higher levels of both NAD^+^ and NADH under low Cu (Figure 2C; Supplemental Figure S6C). Taken together, our observations are consistent with a simple model in which the high availability of sugars in *spl7*-*1* feeds into the production of reductants in the form of NADH. The high levels of NADH in turn fuel respiratory electron flow and ATP production, with normal levels of COX activity and increased AOX activity. AOX partially uncouples NADH oxidation in the mETC from ATP production and can thus function in redox balancing (Vanlerberghe, 2013), but its increased activity in *spl7-1* may be insufficient to prevent the excess of NADH and ATP. This model does not yet provide an explanation for the accumulation of sugars in the *spl7-1* mutant.

We analyzed the levels of metabolites associated with respiratory and sugar metabolism in the shoots of 21-day-old seedlings grown on low-Cu and control solid agar media with no added sucrose. In addition to WT and *spl7-1* seedlings, we included two previously characterized Cu-deficient Arabidopsis lines, the *paa1-3* mutant (Shikanai et al., 2003; Abdel-Ghany et al., 2005) and a transgenic artificial microRNA (amiR)*-FRO4/5* line producing an amiR directed against *FRO4* and *FRO5* under the control of the CaMV 35S promoter (Bernal et al., 2012). In *paa1-3*, severe photosynthetic defects resulting from impaired chloroplastic Cu import and compromised PC function can be rescued by high levels of exogenous Cu. The amiR*-FRO4/5* lines are partially impaired in the reduction of Cu(II) to Cu(I) at the cell surface, which is required for high-affinity cellular Cu uptake in low-Cu medium (Bernal et al., 2012).

We observed few and quantitatively minor changes in metabolite concentrations in the WT under Cu deficiency compared to control conditions, none of which were consistent across independent experiments (Figure 2D; Supplemental Figure S6, D and E; Supplemental Table S1). Most metabolites were present at higher levels in *spl7*-*1* than in the WT under both low-Cu and Cu-sufficient cultivation conditions. Compared to the WT, we observed the consistently largest alterations in *spl7-1* for the levels of glycerol 3-phosphate (Gly3P), 2-oxoglutarate (2-OG), shikimate, sucrose 6-phosphate (Suc6P), and succinate, especially under low Cu. The profiles of these metabolites in *spl7-1* differed from those in the amiR*-FRO4/5* line, whereas several other sugar- and glycolysis-related metabolites showed similar alterations in both *spl7-1* and amiR*-FRO4/5* compared to the WT (e.g. glucose 1-phosphate [Glc1P] and glucose 1,6-bisphosphate [GBP]). Different from *spl7-1*, the concentrations of nearly all analyzed metabolites were markedly lower in *paa1-3* cultivated in low-Cu medium. Chloroplasts of *spl7-1* contain ∼20% less Cu than those of the WT (Zhang et al., 2014), whereas a 58% reduction in chloroplast Cu levels was reported for *paa1-3* (Abdel-Ghany et al., 2005), suggesting a more severe lack of Cu in *paa1-3* chloroplasts. The opposing trends in metabolite profiles between *spl7-1* and *paa1-3*, together with the specific alterations in *spl7-1* compared to amiR*-FRO4/5*, suggested that the contributions of chloroplast and general physiological Cu deficiency to the metabolite profiles of *spl7*-*1* are minor.

Notably, the two most strongly altered metabolites, Gly3P (57% increase in *spl7*-*1* compared to WT in low Cu) and 2-OG (60% decrease in *spl7*-*1*), both have roles in the shuttling of reducing agents, for example NADH, across the inner mitochondrial membrane as part of the glycerol phosphate shuttle (Shen et al., 2006) and the malate/aspartate shuttle (Journet et al., 1981). The alterations in the levels of Gly3P and 2-OG in *spl7*-*1* are consistent with the mutant being locked in an overall more reduced state.

In accordance with the elevated sugar concentrations seen in *spl7*-*1* (see Figure 1, D and E), we detected higher levels of all monitored sugar metabolism intermediates in *spl7*-*1* under low Cu compared to the WT in at least two out of three independent experiments, with Suc6P levels increasing by 76%, for example (Figure 2D; Supplemental Figure S6, D and E; Supplemental Table S1). Relative to the WT, we observed increased levels of the signaling sugar T6P in *spl7-1* (56% increase compared to WT; Figure 2, D and E; Supplemental Figure S6, D and E; Supplemental Table S1). This was independent of Cu supply in two out of three independent experiments, and T6P levels broadly followed sugar levels, in agreement with a previous report (Lunn et al., 2006). Consequently, growth defects in *spl7-1* cannot be attributed to a depletion in the signaling sugar T6P (Schluepmann et al., 2003; Figueroa and Lunn, 2016). In an experiment carried out under low-Cu conditions alone, we confirmed elevated T6P levels in *spl7-1* and *spl7-2*, as well as their decrease down to WT levels in an *spl7-2* complemented line (Supplemental Table S2, Experiment 4). Together, these results suggest that the primary metabolite profile of *spl7* mutants is rather different from that of other Cu-deficient mutants, and thus they implicate SPL7-dependent processes, rather than pleiotropic defects common to Cu-limited plants, in reduced growth despite the high energy status of *spl7*.

### Major energy signaling pathways are generally functional in the *spl7-1* mutant

**Figure 3:**
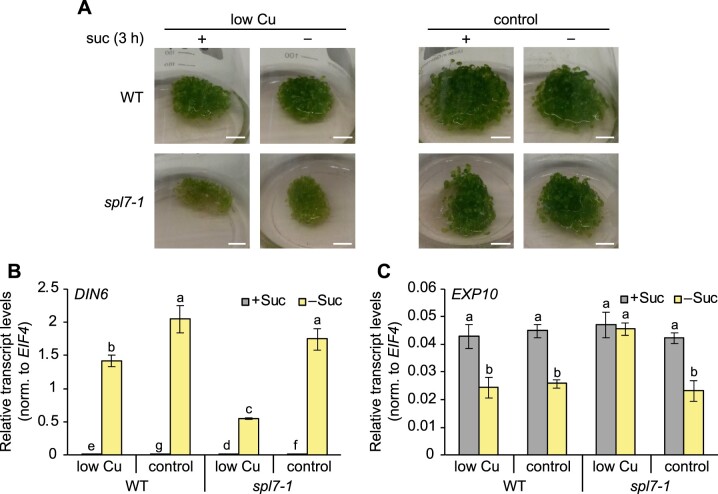
SnRK1 pathway activation in response to sucrose starvation in WT and *spl7-1* mutant seedlings. A, Photographs of 15-day-old seedlings cultivated in liquid medium under low-Cu (no CuSO_4_ added) or control (1 µM CuSO_4_) conditions with 0.5% (w/v) sucrose following a 3-h sucrose starvation treatment (no sucrose). Scale bar = 1 cm. B and C, Relative transcript levels for the SnRK1 pathway marker genes *DIN6* (induced by SnRK1 activity) and *EXP10* (repressed by SnRK1 activity) in the shoots of seedlings cultivated as described for (A), as quantified by RT**–**qPCR. Data are means ± sd (*n* = 3 technical replicates, that is independent PCR runs, each with three replicate wells per transcript). Transcript levels were normalized to those of *EIF4* as a constitutively expressed control gene. Different lowercase letters denote significant differences (*P* < 0.05) between means based on *t* tests with FDR adjustment (*q*-value < 0.05) (B) or ANOVA (Tukey’s HSD) (C).

Next, we examined sugar-dependent signaling, which connects energy status with growth and development (Baena-Gonzalez and Hanson, 2017). The kinase SnRK1 is activated in response to a low energy status, acting to repress energy-consuming processes and growth, and is inhibited by sugars, likely in the form of sugar phosphates such as T6P (Baena-González et al., 2007; Zhang et al., 2009; Nunes et al., 2013a; Zhai et al., 2018). To test the functionality of the SnRK1-activating pathway in *spl7-1* based on marker transcripts, we employed a submerged liquid cultivation system in flasks, which allows the rapid exchange of medium for 3-h sucrose starvation treatments (Nunes et al., 2013a). In response to sucrose starvation, relative transcript levels of *DARK INDUCIBLE6* (*DIN6)*, a marker SnRK1-activated gene, were strongly upregulated in both the WT and *spl7-1*, irrespective of Cu supply (Figure 3, A and B). This result indicated an ability to activate SnRK1 throughout. However, transcript abundance of *DIN6* was clearly lower in *spl7-1* compared to WT under low-Cu conditions. Relative transcript levels of *EXPANSIN10* (*EXP10)*, a marker gene that is downregulated by increased SnRK1 activity, decreased in response to sucrose starvation in all samples except for *spl7-1* under low Cu (Figure 3C). Together, these observations suggest that SnRK1 activity reflects sugar levels in *spl7-1*, congruent with our interpretation that the SnRK1 pathway is sugar responsive and thus generally functional in the mutant.

**Figure 4:**
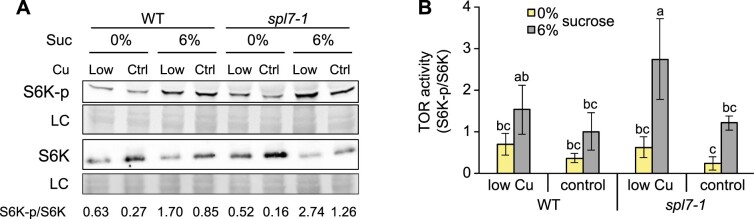
TOR activity in WT and *spl7-1* mutant seedlings. A, Immunodetection of phosphorylated (S6K-p) and total S6K (S6K1 and S6K2) protein as a marker of TOR activity. Data are from shoots of 21-day-old WT and *spl7-1* seedlings cultivated in vertically oriented glass petri plates on low-Cu (0.05 µM CuSO_4_) or control (0.5 µM CuSO_4_) EDTA-washed agar solid medium without sucrose (0%) or supplemented with 6% (w/v) sucrose for 14 days. Total protein extracts were separated by SDS–PAGE and transferred to nitrocellulose membranes. Proteins were visualized on the membrane through Ponceau S staining as a loading control (LC) prior to immunodetection (S6K-p, S6K apparent sizes 52 kDa). The ratio of S6K-p/S6K band intensities is shown below each lane. Blots from two additional independent experiments are shown in Supplemental Figure S7. B, Ratios of S6K-p/S6K band intensities for immunoblot images shown in (A) and Supplemental Figure S7. Data are means ± sd (*n* = 3 replicate blots from independent experiments). Different lowercase letters denote significant differences (*P* < 0.05) between means based on ANOVA (Tukey’s HSD).

The activity of the second central regulator in plant energy signaling, the TOR kinase, is stimulated by sugars to promote growth and development (Deprost et al., 2007; Xiong et al., 2013). To assess TOR activity, we analyzed the phosphorylation state of its downstream target S6 kinase (S6K) as a marker in seedlings cultivated in our combined Cu (low Cu and control) and sucrose (0% and 6%) growth conditions, using modification-specific anti-S6K antibodies (Dong et al., 2017). In seedlings grown in the presence of 6% sucrose, compared to no added sucrose (0%), the ratio of phosphorylated relative to nonphosphorylated S6K protein was at least as high in *spl7-1* as in WT seedlings, independently of Cu supply (Figure 4; Supplemental Figure S7). This observation is consistent with the known activation of TOR by glucose and sucrose and indicated that TOR-mediated sugar signaling is generally functional in *spl7-1* (Xiong and Sheen, 2012; Xiong et al., 2013; Dobrenel et al., 2016). Paralleling the levels of internal sugars (see Figure 1, D and E), the relative abundance of phosphorylated S6K protein was higher in *spl7-1* than in the WT upon cultivation in medium containing 6% sucrose, and the difference between genotypes was more pronounced and more consistently observed when seedlings were grown in low-Cu medium (Figure 4; Supplemental Figure S7). Taken together, our data implicate a process that acts either independently, or downstream, of the TOR and SnRK1 kinases, in SPL7-dependent energy metabolism.

### Flowering time of *spl7-1*

**Figure 5:**
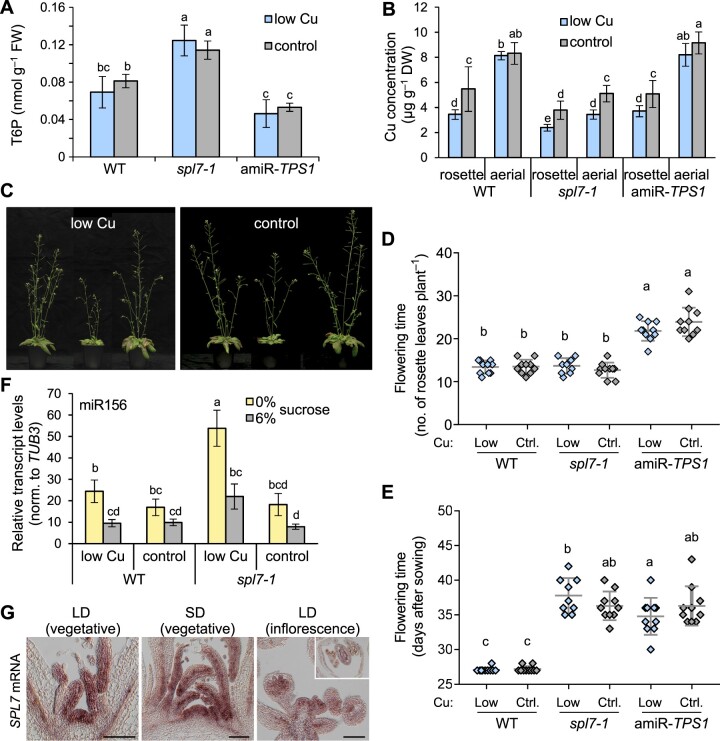
Flowering time of WT, *spl7-1*, and amiR*-TPS1* Arabidopsis plants. A, T6P concentrations. Data are from shoots of 21-day-old WT, *spl7-1*, and amiR*-TPS1* seedlings cultivated in vertically oriented glass petri plates on low-Cu (0.05 µM CuSO_4_) or control (0.5 µM CuSO_4_) EDTA-washed agar solid medium without added sucrose for 14 days. Data are means ± sd (*n* = 4 replicate pools, with one pool per petri plate). Compare to Figure 2E. B, Cu concentrations in rosette and aerial tissues of plants cultivated in soil under LD conditions. Plants were watered with equal amounts of tap water without (low Cu) or with 2 mM CuSO_4_ (control) once per week and harvested 10 days after bolting. Data are means ± sd (*n* = 8 plants per genotype, treatment, and tissue). C, Photographs of WT, *spl7-1*, and amiR*-TPS1* plants (left to right) 10 days after bolting. Plants were cultivated as in (B). D and E, Flowering time, shown as the number of rosette leaves (D) and age at bolting time (E) of plants cultivated as in (B). Data are means (line) ± sd with measurements shown for each individual plant (diamonds) (*n* = 8 plants per genotype and treatment). Two independent experiments are shown in Supplemental Figure S8. F, Relative miR156 levels of in shoots of 21-day-old seedlings. Cultivation was in vertically oriented glass petri plates on low-Cu (0.05 µM CuSO_4_) or control (0.5 µM CuSO_4_) EDTA-washed agar solid medium with no sucrose added (0%) or supplemented with 6% (w/v) sucrose for 14 days, with harvest at ZT 3. Data are means ± sd (*n* = 3 technical replicates, that is, independent PCR runs, each with three replicate wells per transcript) as determined by RT**–**qPCR. Data shown are representative of two independent experiments. G, *SPL7* mRNA detection in the shoot apex by in situ RNA hybridization. Shown are longitudinal sections through apices of WT plants cultivated in soil in (SD) or LD conditions. Inset shows a transverse section of a flower. Scale bars = 100 µm. Different lowercase letters denote significant differences between means (*t* tests with FDR adjustment; *q*-value < 0.05) (A, B, and D–G). T6P: trehalose 6-phosphate.

Sugar signals such as T6P promote growth as well as developmental phase transitions, for example flowering (Schluepmann et al., 2003; Wahl et al., 2013; Yang et al., 2013; Yu et al., 2013; Ponnu et al., 2020). We thus explored possible phenotypic changes in *spl7-1* at later developmental stages. For comparison, we included the transgenic *35S:amiR-TPS1* line (amiR-*TPS1* thereafter) in which T6P levels are reduced as a consequence of the posttranscriptional downregulation of *TREHALOSE 6-PHOSPHATE SYNTHASE1* (*TPS1*) transcript levels, resulting in a strongly delayed flowering time in long days (Wahl et al., 2013). Under our growth conditions, T6P levels were ∼34% lower in amiR*-TPS1* compared to WT independently of Cu supply (Figure 5A), in agreement with published data (Wahl et al., 2013). In *spl7*-*1* mutant plants cultivated alongside, T6P levels were increased to ∼178% of WT levels on low-Cu medium and to 140% of WT levels on control medium (see also Figure 2D; Supplemental Figure S6, D and E; Supplemental Tables S1 and S2).

Our standard greenhouse soil is naturally Cu deficient (low Cu), and the defects in reproduction of *spl7-1* are rescued by watering with 2 mM CuSO_4_ (control) once per week (Schulten et al., 2019). With this supplementation regime, rosette leaves and aerial tissues of *spl7-1* accumulated significantly higher Cu concentrations than plants grown on unamended low-Cu soil (Figure 5B), and the stunted growth of *spl7-1* was partially rescued (Figure 5C), as reported earlier (Yamasaki et al., 2009; Bernal et al., 2012; Garcia-Molina et al., 2014a; Yan et al., 2017). Cu supplementation led to a significant increase in rosette Cu levels to ∼5 µg g^−1^ dry biomass in the WT and amiR*-TPS1*. Cu concentrations in aerial tissues of WT and amiR*-TPS1* were around 8 µg g^−1^ DW, clearly above those in rosette tissues and unaffected by Cu supplementation. In contrast, Cu concentrations in aerial tissues of *spl7-1* were only a little higher than in rosette tissues. This observation suggested that WT plants preferentially allocate Cu into aerial tissues during the reproductive phase of the life cycle in a largely SPL7-dependent manner.

Consistent with published results, amiR*-TPS1* plants flowered later than the WT according to both plant age and the number of rosette leaves at bolting (Figure 5, D and E; Supplemental Figure S8) (Wahl et al., 2013). The *spl7-1* mutant had produced as many, or slightly fewer rosette leaves at bolting than the WT (Figure 5D; Supplemental Figure S8, A and C). Based on plant age, flowering time was clearly delayed in *spl7-1* by ∼7 days compared to WT, irrespective of Cu supply (Figure 5E; Supplemental Figure S8, B and D), and comparable to amiR*-TPS1*. Taken together, these results suggest that, in addition to growth, the transition to flowering is partially uncoupled from sugar levels in *spl7-1*. Consistent with a known repression of miR156 abundance by metabolizeable sugars and T6P (Wahl et al., 2013; Yang et al., 2013; Yu et al., 2013; Ponnu et al., 2020), miR156 levels were lower in the shoots of WT and *spl7-1* seedlings grown in the presence of 6% sucrose compared to no sucrose added (Figure 5F). This result confirmed that miR156 levels remain responsive to internal sugar levels in *spl7-1* (Yang et al., 2013; Yu et al., 2013). However, despite elevated sugar levels in *spl7-1* compared to the WT (see Figures 2 and 5A), miR156 were higher in *spl7-1* relative to the WT when cultivated under low-Cu conditions (Figure 5F). Thus, the known sugar-dependent decrease in miR156 levels with plant age is attenuated in the mutant in low Cu.

Flowering requires a developmental switch at the apical meristem. Therefore, we investigated whether SPL7 might locally influence this switch. RNA in situ hybridization revealed the presence of *SPL7* transcripts at the shoot apical meristem and in young leaf primordia of vegetative plants cultivated in both SD and LD, as well as in reproductive shoot apices (Figure 5G). Several miR156 targets, for example, *SPL9*, *SPL13*, and *SPL15*, are also expressed in the shoot apical meristem (Wang et al., 2009; Yamaguchi et al., 2014b; Hyun et al., 2016; Xu et al., 2016). The function of SPL7 in the shoot apical meristem warrants further investigation.

### Global identification of SPL7-binding sites

**Figure 6:**
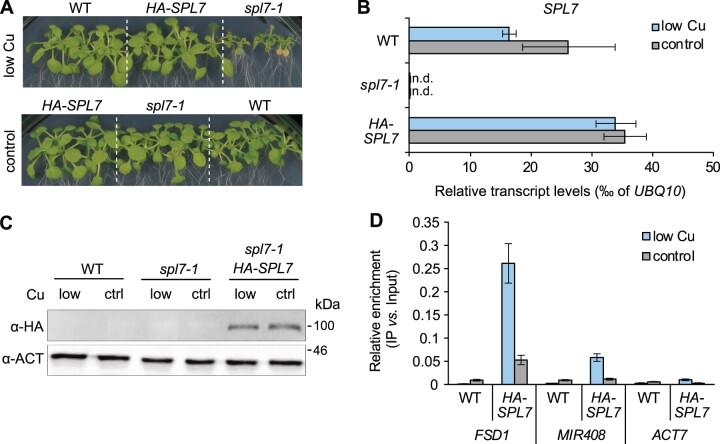
Complementation of the *spl7-1* mutant by the *SPL7prom:HA-SPL7-HA:SPL7term* transgene. A, Photographs of the WT, the *spl7-1* mutant and a selected transgenic homozygous *spl7-1 SPL7prom:HA-SPL7-HA:SPL7term* (*HA-SPL7-HA*) line. Shown are 21-day-old seedlings cultivated in low-Cu (no CuSO_4_ added) or control (0.5 µM CuSO_4_) medium supplemented with 1% (w/v) sucrose and solidified with EDTA-washed agar in vertically oriented glass petri plates. B, Relative *SPL7* transcript levels according to RT**–**qPCR, in the shoots of seedlings cultivated as in (A). Data are means ± sd (*n* = 3 technical replicates, that is independent PCR runs, each with three replicate wells per transcript). Data from two additional transgenic lines are shown in Supplemental Figure S9. C, Immunoblot detection of HA-SPL7-HA (expected at ∼90 kDa) in shoots of WT, *spl7-1*, and *HA-SPL7-HA* seedlings cultivated as in (A). PVDF membranes were stripped and reprobed with α-ACTIN (ACT) antibody (shown as an LC). The full image and results from an independent experiment are shown in Supplemental Figure S10. D, Validation of ChIP prior to sequencing using ChIP-qPCR of previously implicated direct SPL7 target genes. Data are mean ± SD (*n* = 3 technical replicates on a PCR plate) of relative DNA enrichment of the promoter regions of *FSD1, MIR408* as a positive control gene, and *ACT7* (negative control gene), quantified by ChIP-qPCR on immunoprecipitation (IP) and input samples. Chromatin was isolated from shoot tissues of *HA-SPL7-HA* and WT seedlings (WT, negative control) cultivated in R 1 (see Figure 7A). Input samples represent aliquots taken after chromatin shearing and before the addition of α-HA for the IP.

We reasoned that expanding our knowledge on direct target genes of SPL7-dependent transcriptional regulation could provide mechanistic insights into how SPL7 affects energy metabolism. To globally identify SPL7 DNA-binding sites using ChIP-seq, we generated transgenic lines harboring an *SPL7* genomic construct encoding SPL7 flanked by HA-tags at the N- and the C-termini, in the *spl7-1* genetic background (*SPL7prom:HA-SPL7-HA:SPL7term*; *HA-SPL7-HA* thereafter). Of the three homozygous lines complementing the *spl7* phenotype under low-Cu cultivation conditions, we chose one line with *SPL7* transcript levels similar to those in the WT (Figure 6, A and B; Supplemental Figure S9). Relative *SPL7* transcript levels were unaffected by Cu levels in the medium, as reported previously (Yamasaki et al., 2009; Bernal et al., 2012). Likewise, an anti-HA antibody detected the HA-SPL7-HA fusion protein on immunoblots under both low-Cu and control conditions (Figure 6C; Supplemental Figure S10). This observation is in line with a proposed posttranslational mechanism for the regulation of the activity of SPL7 as a transcriptional enhancer, similar to Chlamydomonas COPPER RESPONSE REGULATOR1 (CRR1) (Kropat et al., 2005; Sommer et al., 2010). We detected the HA-SPL7-HA protein as a single band running with an apparent molecular mass of around 100 kDa, which was slightly higher than the predicted protein mass including the HA-tags of ∼92 kDa (Figure 6C; Supplemental Figure S10). We conducted ChIP on rosette tissues of 21-day-old *HA-SPL7-HA* seedlings cultivated in low-Cu and control conditions. ChIP-qPCR confirmed an enrichment relative to chromatin input for the known SPL7 target gene *MIR408* (Zhang and Li, 2013), and the expected SPL7 target gene *FSD1* (Zhang et al., 2014), in *HA-SPL7-HA* samples relative to the WT, in contrast to the negative control gene *ACTIN7* (*ACT7*) (Figure 6D).

**Figure 7:**
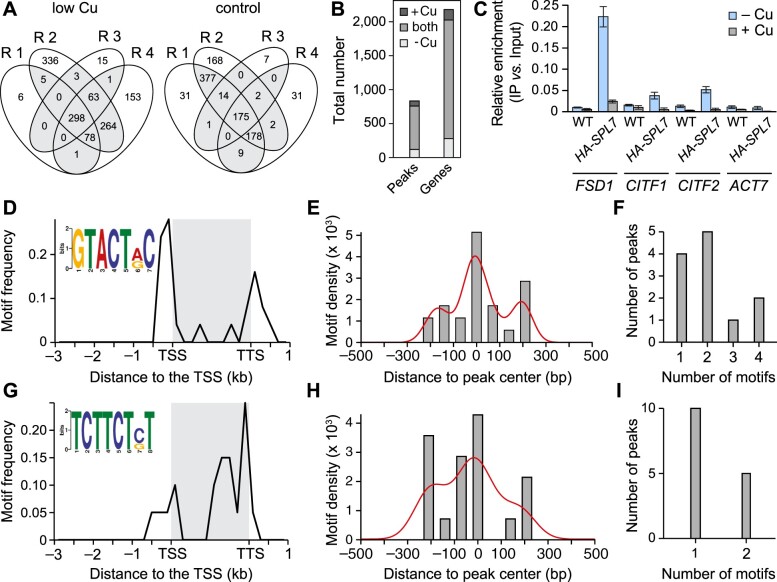
Global identification of SPL7 DNA-binding sites. A, Venn diagrams showing the reproducibility of results. Given are the numbers of SPL7-binding segments (peaks) identified by ChIP-seq in each of four independent experiments (each with independent plant cultivation, denoted as R 1 to R 4) across the genome. ChIP-seq was conducted on shoots of 21-day-old seedlings cultivated on control (0.5 µM Cu) and low-Cu (no Cu added) medium supplemented with 1% (w/v) sucrose and solidified with EDTA-washed agar in vertically oriented glass petri plates. B, Number of SPL7-binding segments identified by ChIP-seq (shown on a dark background in A) and associated loci. C, Validation of ChIP-seq results by ChIP-qPCR. Data are mean ± sd (*n* = 3 technical replicates on a PCR plate) of relative DNA enrichment of the promoter regions of novel candidates *FSD1*, *CITF1*, *CITF2* (*bHLH23*), with *ACTIN7* (*ACT7*) as a negative control. Chromatin was independently isolated from a different replicate pool of shoots of *HA-SPL7-HA* and WT seedlings (WT, negative control) cultivated in R 1 (see A), generating independent immunoprecipitate (IP) and input samples. Input samples represent aliquots taken after chromatin shearing and before the addition of α-HA for the IP. See Supplemental Figure S12, J and K for two additional independent experiments. D–I, The GTACTRC motif (D–F, *E*-value = 8.20 × 10^−3^, 21 motif sites) and the TCTTCTST motif (G–I, *E*-value = 3.40 × 10^−2^, 19 motif sites) identified by MEME motif analysis. Motif frequency positional distribution (200-bp bin size), with conservation logo generated by MEME as an inset (D and G). Density plot summarizing the distances of motif from the center of peaks from ChIP-seq (75-bp bin size) (E and H). Number of motif copies within single peaks (F and I). The gene body (gray box) of all corresponding genes was normalized to 2,000 bp (D and G). The curved line indicates the shape of the distribution (E and H). TSS: transcriptional start site, TTS: transcriptional termination site (positions from Arabidopsis TAIR10 genome annotation).

Next, we globally identified putative SPL7-binding sites based on sequencing of input and ChIP sample pairs. In low-Cu conditions and control Cu conditions, respectively, we identified 758 peaks/2,026 genes and 713 peaks/1,901 genes as containing SPL7-binding sites, with support from at least two (out of four) experiments, each with independent plant cultivation and sample processing (Figure 7, A and B; Supplemental Data Sets 1 and 2). Out of the 655 peaks identified here to contain putative SPL7-binding sites under both low-Cu and control Cu conditions (Figure 7B), both negative controls of a single replicate of ChIP-seq on the WT cultivated in low-Cu and control Cu conditions, respectively, identified segments overlapping with 119 peaks, which may originate from cross-reactions of the anti-HA antibody with DNA-bound proteins other than HA-SPL7-HA (Supplemental Data Set 2). Note that read coverage for negative control WT ChIP-seq samples was low after correction for input, and the resulting peaks were narrower and frequently included stacks of identical reads, which we attribute to low DNA amounts used in library preparation. Around 9% of the genes associated with genomic segments identified here using SPL7 ChIP-seq were among 1,266 genes previously identified based on a single SPL7 ChIP-seq experiment (Zhang et al., 2014) (more than expected by chance; *P* < 10^−15^, hypergeometric test; Supplemental Table S3). Different from the earlier study by Zhang et al. (2014), by far the most frequently observed localization of peaks was 200 to 20 bp upstream of the predicted transcriptional start site (Supplemental Figure S11). Possible explanations for these differences between studies may lie in distinct plant cultivation conditions, the earlier expression of *FLAG-SPL7* under the control of the CaMV 35S promoter, or the fact that these FLAG-SPL7 ChIP-seq data were apparently based on a single replicate (Zhang et al., 2014).

To evaluate genomic SPL7-binding sites based on *SPL7*-dependence of transcript levels, we conducted transcriptome deep sequencing (RNA-seq) on rosette tissues of WT and *spl7* seedlings cultivated as for ChIP-seq. Out of the top eight candidate genes for *SPL7*-dependent transcriptional activation identified through the largest *SPL7*-dependence at the transcript level under low-Cu conditions (Supplemental Data Set 3), five genes were also identified in our ChIP-seq under low-Cu conditions: *FSD1*, the bHLH transcription factor gene *bHLH160* (*CITF1*; Yan et al., 2017), *bHLH23* (addressed here as *CITF2*), *YELLOW STRIPE-LIKE2* (*YSL2)*, and *ZINC-REGULATED TRANSPORTER, IRON-REGULATED TRANSPORTER-RELATED PROTEIN2* (*ZIP2*) (Table 1; Supplemental Figure S12, A–I; Supplemental Data Set 2). Of these, we tested three genes, *FSD1*, *CITF1*, and *CITF2*, in independent ChIP-qPCR experiments and confirmed all three (Figure 7C; Supplemental Figure S12, J and K). This result supported the role of SPL7 in transcriptional activation under low-Cu conditions (Kropat et al., 2005).

**Table 1:** Genes undergoing maximal *SPL7*-dependent transcriptional regulation and microRNA loci associated with SPL7-binding sites according to ChIP-seq on shoot tissues of seedlings cultivated on copper-deficient medium

			TL^a^	RTL (WT versus *spl7*)^b^		ChIP-seq peaks^c^		Identified by DAP-seq^d^					
AGI code	Short name	Annotation	TPM	Low Cu	Control Cu	Low Cu	Control Cu	SPL1	SPL5	SPL9	SPL13	SPL14	SPL15 ampl.
At4g25100	*FSD1*	Fe SUPEROXIDE DISMUTASE1	1800	4,700	1,500	4G^e^^,^^f^	None	Y		Y	Y	Y	Y
At1g71200	*BHLH160, CITF1*	Cu-DEFICIENCY-INDUCED TRANSCRIPTION FACTOR1	3	390	–	3P^e^	None	Y		Y	Y	Y	Y
At5g59520	*ZIP2*	ZRT/IRT-like PROTEIN2	0.86	32	–	2G^e^	None	Y		Y	Y	Y	Y
At4g28790	*BHLH23, CITF2*	Cu-DEFICIENCY-INDUCED TRANSCRIPTION FACTOR2	2.2	18	–	3P^e^	None	Y		Y			Y
At5g24380	*YSL2*	YELLOW STRIPE-LIKE2	30	13	5.24	3G^e^	None	Y		Y			Y
At3g23326	*MIR853a*	Unknown function	NA	NA	NA	4P, 2P	4P, 2P						
At5g14545	*MIR398B*	Targets both CSD and CytC oxidase family members	NA	NA	NA	4G^g^	None	Y		Y	Y	Y	Y
At5g14565	*MIR398C*	Targets both CSD and CytC oxidase family members	NA	NA	NA	4P, 4G^g^	None	Y		Y	Y	Y	Y
At2g47015	*MIR408*	Targets a LACCASE and PLANTACYANIN-like family member	NA	NA	NA	3S^h^	None	Y	Y	Y	Y	Y	Y
At4g13554	*MIR857a*	Targets a LACCASE family member	NA	NA	NA	3P^h^	None						
At4g13555	*MIR397B*	Targets several LACCASE family members	NA	NA	NA	3G^h^	None	Y		Y	Y	Y	Y
At5g26038	*MIR860a*	Unknown function	NA	NA	NA	3G	4G		Y	Y			Y
At1g19371	*MIR169H*	Targets several HAP2 family members	NA	NA	NA	2P	None		Y	Y			Y

^a^TL (normalized transcript levels in transcripts per million, TPM, in WT low Cu).

^b^Relative transcript levels (RTLs) according to RNA-seq data (Supplemental Data Set 3).

^c^ChIP-seq peaks: number of replicates identifying peaks, followed by localization: P (promoter), S (overlapping start), G (gene body).

^d^DAP-seq data were taken from O’Malley et al. (2016); ampl., amplified; Y, locus identified; NA, no data obtained; –, ratio not significantly different from 1.

^e^Two GTACTRC motifs (see Figure 7D; Supplemental Data Set 6).

^f^At least two nonconsensus upstream GTAC core motifs (Supplemental Figure S15).

^g^One GTACTRC motif (Supplemental Data Set 4).

^h^Two GTACTRC motifs (Supplemental Data Set 4).

All SPL family members are thought to share a core GTAC motif in their DNA-binding sites (Birkenbihl et al., 2005). The numbers of potential target genes shared between SPL7 (from our SPL7 ChIP-seq data) and SPL1, SPL5, SPL9, SPL13, SPL14, or SPL15 (based on DNA Affinity Purification sequencing [DAP-seq] from O’Malley et al. (2016)), or those in common across all of the latter SPL proteins, were not significantly above expectations based on random picking (Supplemental Table S3; Supplemental Data Set 2). Importantly, the top five genes highlighted above also all contained binding sites for three to five of the other SPL family members based on DAP-seq data (Table 1). There was a similarly large agreement between SPL DAP-seq data and the miRNA loci previously identified as direct targets of SPL7 binding and SPL7-mediated transcriptional activation, miR408, miR398B, and miR398C (Yamasaki et al., 2009; Zhang and Li, 2013), which were again confirmed here by SPL7 ChIP-seq (Table 1).

Examining the sequences of all peaks identified by SPL7 ChIP-seq together, we failed to identify any enriched sequence motif (Supplemental Data Set 4). Restricting our analysis to ChIP-seq peaks unique to low Cu, we identified the significantly enriched **GTAC**TA/GC motif that is partially identical to the previously reported A/T**GTAC**T/A, as well as significantly enriched AGGAAGC/T (reverse complement: A/G**CTTCCT**) that is reminiscent of TCTTCTTCTC**CTTCCT**C (Zhang et al., 2014) (Supplemental Figure S13). The subfamily II SPL protein Ideal Plant Architecture 1 (IPA1, also named SPL14) of rice (*Oryza sativa*) binds to differing core motifs, either GTAC or TGGGCC/T, as a function of environmental conditions (Wang et al., 2018). To further accommodate possible alternative SPL7-binding preferences dependent on Cu supply, we generated differing sets of peaks based on the transcriptional regulation of the associated genes for subsequent motif identification (Supplemental Data Sets 5 and 6). In this manner, we obtained 51 and 52 candidate genes for direct activation and repression, respectively, by SPL7 under low-Cu conditions (Supplemental Data Set 5). Note that binding site positions predominantly upstream of transcriptional start sites were equally consistent with *SPL7*-dependent activation and repression in low-Cu conditions (compare Supplemental Figure S11, D and F). Under control cultivation conditions, only 16 and 24 genes were candidates for undergoing direct SPL7-mediated activation and repression, respectively (Supplemental Data Set 5). SPL7-binding sites were positioned upstream or within the gene body at similar frequencies, suggesting either a lesser or a more complex role of SPL7 in transcriptional regulation under Cu-sufficient conditions (compare Supplemental Figure S11, C and E).

We identified enriched motifs **GTAC**TA/GC and AC/GAGAAGA (TCTTCTC/GT) among ChIP-seq peaks associated with genes undergoing *SPL7*-dependent transcriptional activation under low-Cu conditions (Figure 7, D–I). Overall motif positioning, density, and abundance supported the GTACTRC motif more strongly than the TCTTCTST motif. Applying progressively relaxed filtering criteria for assembling sets of ChIP-seq peak-associated and *SPL7*-dependently regulated genes, we identified multiple sequence motifs, some of which contain previously reported binding sites of other transcription factors (Supplemental Figure S14; Supplemental Data Sets 4 and 6). Four motifs newly identified here were predominantly positioned within gene bodies (Supplemental Figure S14, A, D, G, and I), similar to the other non-GTAC-containing motifs (see Figure 7G; Supplemental Figure S13D). Taken together, these results are consistent with a possible direct transcriptional activation of *FSD1*, *CITF1*, and *CITF2*, alongside a small set of other genes, by SPL7 under low Cu. Beyond these results, our data are consistent with complex roles and interactions of SPL7 (Zhang et al., 2014; Garcia-Molina et al., 2014a; Yan et al., 2017).

Despite strong support for both positive and direct regulation of *FSD1* transcript levels by SPL7 (Table 1; Figures 6, D and 7, C; Supplemental Figures S4, D, S12, J, and K), MEME (Multiple Expectation maximizations for Motif Elicitation) did not identify the *FSD1* promoter as containing any of the enriched sequence motifs above (Supplemental Data Sets 4 and 6). Manual inspection identified eight GTAC core motifs in the region corresponding to the *FSD1*-associated peak, with at least four copies of the GTAC core motif upstream of all annotated alternative transcriptional start sites of *FSD1* (Supplemental Figure S15). Two of these copies correspond to **GTAC**TRC, the best supported motif identified here (see Figure 7D; Supplemental Figure S13A). There are also two copies of A**GTAC**A/T**GTAC**T, previously concluded to have the highest in vitro binding affinity for SPL7 out of several variants tested using electrophoretic mobility shift assays (Zhang et al., 2014).

## Discussion

### How do altered sugar responses arise in the *spl7* mutant?

A Cu limitation of photosynthesis would have offered a plausible explanation for the growth defect of *spl7* mutants cultivated under low-Cu conditions (see Figure 1), given the known requirement for SPL7 in the transcriptional activation of Cu acquisition and Cu economization processes (Yamasaki et al., 2009; Bernal et al., 2012). However, the unresponsiveness of growth of *spl7* mutants to exogenous sucrose in low Cu and an enhanced accumulation of sugars in *spl7-1* implicated processes other than photosynthetic assimilate production in its growth defect (Figure 1; Supplemental Figures S2–S4).

Electron flow via the COX-dependent cytochrome *c* pathway was not affected by Cu deficiency in WT Arabidopsis (Figure 2), consistent with normal COX2 protein levels in Arabidopsis and black cottonwood (*Populus trichocarpa*) cultivated on low-Cu medium (Yamasaki et al., 2007; Ravet et al., 2011). Interestingly, we observed no reduction in electron flux via the cytochrome *c* pathway in low-Cu-grown *spl7-1*. A slight SPL7-independent increase in *HOMOLOGUE OF THE COPPER CHAPERONE SCO1* transcript levels, encoding a chaperone involved in Cu delivery to COX (Attallah et al., 2011), under Cu deficiency may contribute to COX functionality in Cu-deficient Arabidopsis (Bernal et al., 2012). It is possible that *spl7-1* seedlings can only maintain adequate Cu supply to COX because of their attenuated growth (Bernal et al., 2012). An elevated total respiration rate in the *spl7-1* mutant under low Cu resulted from the activation of AOX-dependent respiration and may have a compensatory role in the presence of elevated ATP levels and NADH/NAD^+^ ratios in *spl7-1* (Figure 2; Supplemental Figure S6). The increased engagement of the alternative electron pathway in response to sugar stimuli is thought to constitute a balancing mechanism for an excess of reducing equivalents (Lambers, 1982; Azcón-Bieto et al., 1983). Enhanced respiration, increased NADH/NAD^+^ ratio, accumulation of ATP, and an altered metabolite profile in *spl7-1* are metabolic features different from the properties of COX-defective plants (Dahan et al., 2014), severely Cu-limited WT plants (Rahmati Ishka and Vatamaniuk (2020), and other Cu-deficient genotypes (Figure 2D). We interpret these metabolic phenotypes of *spl7* mutants as consequences of an elevated sugar status, and not as its causes.

As we excluded major contributions from defects in the two most prominent Cu-dependent metabolic pathways, the attenuation of growth seen in *spl7-1* under low Cu despite high internal sugar levels might result from defective sugar/energy sensing or signaling. SnRK1 kinase activity is directly inhibited by T6P (Zhang et al., 2009; Zhai et al., 2018), a signaling metabolite that accumulated in *spl7-1* (Figures 2 and 5; Supplemental Figure S6). The responsiveness of downstream marker transcripts of the SnRK1 signaling pathway was consistent with its general functionality in *spl7-1* (Figure 3). The phosphorylation state of the TOR kinase target S6K was also in accordance with sugar levels in *spl7-1*, indicating that the TOR pathway is generally functional in *spl7-1* under low Cu (Figures 1 and 4; Supplemental Figures S3 and S7) (Xiong and Sheen, 2012; Dobrenel et al., 2016). Different from these observations in *spl7-1*, TOR activity decreases under nitrogen starvation conditions (Liu et al., 2021). To summarize, our data argue against an involvement of TOR and SnRK1 kinases in the SPL7-dependent integration of Cu deficiency responses with the energy-dependent regulation of growth in Arabidopsis. Yet, we cannot exclude a possible partial attenuation of SnRK1 or TOR signaling in *spl7*.

Different from *spl7-1*, failure to grow as a consequence of impaired sulfur assimilation in the Arabidopsis *sulfite reductase1-1 (sir1-1*) mutant, which lacks the enzyme sulfite reductase, was linked to reduced TOR activity (Dong et al., 2017). In agreement with sugar signals governing TOR activity, the *sir1-1* mutant contained lower carbohydrate levels, different from *spl7*. Finally, transcript levels of published targets of nuclear HEXOKINASE1 (HXK1) signaling function *CARBONIC ANHYDRASE2* (*CA2*) and *LIGHT HARVESTING CHLOROPHYLL A/B-BINDING PROTEIN1.1* (*LHCB1.1*) (Cho et al., 2006), as well as of the proposed downstream target of HXK1-dependent metabolic regulation, *NITRATE TRANSPORTER2.*1 (*NRT2.1*) (Lejay et al., 2003, 2008), appeared to be regulated as expected based on higher sugar contents in *spl7-1* (Supplemental Data Set 3; *CA2*, *LHCB1.1* four-fold down, *NRT1.2* eight-fold up in *spl7-1* versus WT under low Cu). The combination of Cu deficiency and the lack of SPL7 function might generate sink-limited conditions by affecting unknown processes downstream of the T6P/SnRK1 module. Under sink-limited environmental conditions, namely low nitrogen supply or low temperature, the strongly inter-related levels of sucrose, T6P, and SnRK1 activity were proposed to be disconnected from the growth outcome (Nunes et al., 2013b). REGULATOR OF G-PROTEIN SIGNALING1 (RGS1) was suggested to operate as a plasma membrane-localized sensor of extracellular glucose (Urano et al., 2012). The activation of RGS1-dependent signaling required unexpectedly high extracellular glucose concentrations (Li and Sheen, 2016). A possible defect in RGS1-dependent signaling in *spl7* deserves attention in future work.

### *SPL7* and Cu deficiency in plant development

Our results are consistent with an attenuated response of developmental transitions to internal sugar levels in *spl7-1*. Compared to the WT, flowering of *spl7-1* was delayed based on plant age and unaltered by Cu supplementation. We observed this result despite the fact that at a given age of *spl7-1*, the levels of the metabolizeable sugars sucrose and glucose increased, and T6P concentrations were similarly elevated irrespective of whether plants were cultivated in low-Cu or Cu-supplemented conditions (Figures 1 and 5; Supplemental Figures S3 and S8). Although they remained sugar-responsive in the *spl7-1* mutant, miR156 levels were strongly elevated in the mutant upon cultivation in low-sucrose low-Cu conditions despite comparably high internal sugar levels, and miR156 could thus contribute to a condition-specific developmental delay (Figures 1 and 5, F). It is unlikely, however, that elevated levels of miR156 cause the sugar accumulation of *spl7-1*, because sugar levels including T6P are not elevated in miR156-overexpressing plants (Ponnu et al., 2020). In Arabidopsis, eight distinct loci encode miR156 precursors, and not all precursor transcripts decrease in abundance in response to sugars (Yang et al., 2013). Moreover, various abiotic stresses, such as heat stress, phosphate starvation, salt stress, and drought, lead to increased miR156 levels (Hsieh et al., 2009; Cui et al., 2014; Stief et al., 2014). Note that a delay in the transition from vegetative to reproductive stage (based on leaf number) was also reported in severely Cu-limited hydroponically cultivated WT Arabidopsis plants (Rahmati Ishka and Vatamaniuk, 2020). This finding was in accordance with a decrease in *FLOWERING LOCUS T* (*FT*) expression, but contradictory to the strongly increased transcript levels of the floral marker *MIR172C* and *MIR172D* transcript levels, in Cu-limited WT plants (Rahmati Ishka and Vatamaniuk, 2020).

The elevated Cu levels in aerial tissues compared to rosette leaves in the WT indicated that Cu allocation to the inflorescence is prioritized, in agreement with the previously established role of Cu in plant fertility, for example in the Cu-binding protein plantacyanin involved in pollen tube guidance (Kim et al., 2003; Dong et al., 2005; Yan et al., 2017; Rahmati Ishka and Vatamaniuk, 2020). This prioritization was to a large extent dependent on SPL7 (Figure 5B). Employing in situ RNA hybridization, we detected a signal for *SPL7* mRNA in the vasculature of the shoot apical meristem (Figure 5G). Future work will be required to analyze possible functions of SPL7 in the shoot apical meristem and during the reproductive phase of development.

### Genomic targets of SPL7 binding and transcriptional regulation

All SPL family members are characterized by a highly conserved recognition domain for the binding to a GTAC core DNA motif, and there is functional overlap among subfamily II SPLs in the regulation of plant development (Birkenbihl et al., 2005; Xing et al., 2010, 2013; Xu et al., 2016). Our phenotypic analysis of *spl7-1* mutants did not support a predominant functional antagonism between SPL7 and miR156-regulated subfamily II SPLs (Figure 5). Alternatively, direct or indirect target genes of regulation by the transcription factor SPL7 could include critical functions in sugar sensing, signaling, or utilization. The combined analysis of genomic SPL7-binding sites and *SPL7*-dependent regulation of transcript levels did not reveal any evident well-characterized genes directly targeted by SPL7 and likely to cause the altered energy metabolism observed in *spl7-1* (Supplemental Data Set 5). Consequently, it is possible that SPL7 is required for the expression of an as-yet uncharacterized gene critical for sugar utilization.

The putative direct SPL7 target genes *CITF1* (*bHLH160*) and *CITF2* (*bHLH23*) identified here were reported as differentially regulated between the WT and *spl7-2* in previous studies (Bernal et al., 2012; Yan et al., 2017). Additionally, our data support *FSD1* as a direct target of SPL7 (Table 1; Supplemental Figures S12 and S15). *FSD1* undergoes the most pronounced *SPL7*-dependent transcriptional regulation (Table 1; Supplemental Figure S4D). *FSD1* was also detected in the earlier ChIP-seq study of SPL7 (Zhang et al., 2014), together with the well-established direct targets of SPL7-dependent transcriptional activation under Cu deficiency, *MIR398B*, *MIR398C*, and *MIR408* (Yamasaki et al., 2009; Zhang and Li, 2013). For all these genes, the positions of ChIP-seq peaks were consistent with SPL7 binding directly to their promoters under Cu deficiency (Table 1; Supplemental Figures S12 and S15). CITF1 belongs to group Ib of bHLH transcription factors, alongside the central Fe-deficiency response regulatory transcription factors bHLH38, bHLH39, bHLH100, and bHLH101 (Heim et al., 2003). Group VIIa of the bHLH transcription factors comprises CITF2 as well as the well-studied PHYTOCHROME-INTERACTING FACTOR (PIF) and PIF-LIKE bHLH proteins. Future work will address possible functions of these transcription factors, as well as of superoxide dismutases, in particular FSD1, in sugar responses and sugar utilization. An example of sugar signaling via reactive oxygen species was recently published (Roman et al., 2021).

Our ChIP-qPCR data suggested the highly effective binding of SPL7 to *FSD1* in Cu-deficient conditions, associated with very high *FSD1* transcript levels, but also notable SPL7 binding even in Cu-sufficient seedlings, as well as both consensus and unusual GTAC-containing sequence elements in the promoter region (Figures 6 and 7; Table 1; Supplemental Figures S12, S13, and S15). Our observations support some residual SPL7 activity even when sufficient Cu is available, implying that at least small amounts of SPL7 are located inside the nucleus and active on a subset of target sites. The transient infiltration of a construct encoding GFP-tagged SPL7 into *Nicotiana benthamiana* leaf epidermal cells suggested that SPL7 is anchored to the endoplasmic reticulum membrane by a transmembrane helix in its C-terminus (Garcia-Molina et al., 2014b). The release of SPL7 into the cytosol and translocation to the nucleus was proposed to occur only under Cu deficiency upon proteolytic cleavage at a site in the center of the protein. Accordingly, the nuclear entry of the N-terminal half of SPL7, which includes the SBP domain, would then allow the transcriptional activation of Cu deficiency-responsive genes (Garcia-Molina et al., 2014b). Different from this proposed mechanism, immunoblots revealed only a single band corresponding approximately to the full protein size, irrespective of plant physiological Cu status, in a stably transformed *HA-SPL7-HA* line (Figure 6; Supplemental Figure S10). Our results do not provide any evidence in support of SPL7 trafficking.

Although we identified several motifs here, based on simple expectations our data supported the consensus GTACTRC motif in promoter regions for the SPL7-mediated transcriptional enhancement primarily under low-Cu conditions (Table 1; Figure 7; Supplemental Figures S12–S15). A shift in the SPL7 regulon under control Cu conditions, as exemplified by *FSD1*, could involve differing cis-regulatory DNA sequence elements that exhibit a higher binding affinity for SPL7, or alternatively as yet unidentified conditional protein interaction partners of SPL7. For example, KIN17 interacts with SPL7 specifically in aerial tissues and is involved in promoting Cu deficiency responses (Garcia-Molina et al., 2014a). SPL7 interacts with ELONGATED HYPOCOTYL5 (HY5) to enhance the levels of miR408 (Zhang et al., 2014). Physical interactions between SPL7 and other transcription factors may help explain the identification of multiple overrepresented motifs among SPL7 ChIP-seq peaks, the apparent binding of SPL7 within gene bodies under Cu-sufficient cultivation conditions, or the predominant localization of peaks in promoter regions of genes repressed at the transcript level in an SPL7-dependent manner (Figure 7, G–I; Supplemental Figures S11, C–F, S13, D–F, and S14; Supplemental Data Sets 4 and 6). We cannot exclude that SPL7 could act alone as a repressor of some of its direct target genes. To date, SPL7-dependent negative regulation was exclusively reported to occur indirectly via SPL7-dependent transcriptional activation of miRNA loci (Abdel-Ghany and Pilon, 2008; Yamasaki et al., 2009).

Another mechanism that can modulate the activity of SPL proteins was described for SPL14/IPA1 in rice, a homolog of Arabidopsis SPL9/15 (Wang et al., 2018). The phosphorylation of a conserved serine residue in the SBP domain caused an altered DNA-binding specificity of OsSPL14, that is a change in preference for binding to a non-GTAC TGGGCC motif. OsSPL14 was thus found to have a dual role in alternatively promoting either yield or disease resistance. If a similar mechanism operated in SPL7, it could explain the additional identification of a non-GTAC SPL7-binding motif (Figure 7, G–I; Supplemental Figures S13, D–F, and S14; Supplemental Data Set 6). Among genes associated with SPL7 ChIP-seq peaks, “response to hypoxia” was strongly overrepresented, but far less so among transcripts for genes regulated by SPL7 (Supplemental Figure S16). Chlamydomonas CRR1 mediates transcriptional responses to both Cu deficiency and hypoxia (Hemschemeier et al., 2013). It now appears relevant to examine whether SPL7 and its orthologs regulate a subset of hypoxia responses in land plants, as well.

Future work will address the complex functions of SPL7 including the molecular mechanisms underlying growth and developmental impairment despite sugar accumulation in *spl7* mutants. Understanding the coordination of plant metal homeostasis with energy metabolism, growth and reproduction can help increase crop yield and quality, especially on soils deficient in bioavailable Cu, which comprise more than 10% of the agricultural land in Europe (Reimann et al., 2014).

## Materials and methods

### Plant materials

WT *A. thaliana* seeds (Columbia-0 [Col-0]) were obtained from Lehle seeds (Round Rock, TX, USA). The *spl7-1* (SALK_093849) and *spl7-2* (SALK_125385) mutants are T-DNA insertion lines obtained from the Nottingham Arabidopsis Stock Centre (NASC) and were characterized previously (Yamasaki et al., 2009; Bernal et al., 2012). The generation of the *spl7-2 SPL7* complemented line (*spl7-2_*C) and transgenic *35S:amiR-FRO4/FRO5* plants (amiR-*FRO4/5*, line 27) was described in Bernal *et al.* (2012). The *paa1-3* loss-of-function mutant was a kind gift from Prof. Marinus Pilon (Shikanai et al., 2003), and *ran1-1* (N3808) was from NASC. Transgenic line *35S:amiR-TPS1* (amiR-*TPS1*) was kindly provided by Dr. Vanessa Wahl (Wahl et al., 2013). All mutants and transgenic lines are in the Col-0 genetic background. Primers used for genotyping are listed in Supplemental Table S4.

The *HA-SPL7-HA* (*SPL7prom:HA-SPL7-HA:SPL7term*) construct was generated as follows (Lampropoulos et al., 2013). The *SPL7* upstream region (–2,506 to –5 bp, with +1 bp being the beginning of the ATG) was amplified by polymerase chain reaction (PCR) from Col-0 genomic DNA and cloned into the Greengate entry module pGGA000 via BsaI restriction digest. The genomic *SPL7* (At5g18830.1) coding region (translational start to stop codon) was PCR amplified and cloned into the vector pBluescript SK+ (Stratagene/Agilent Technologies, Waldbronn, Germany), which was used a PCR template for site-directed mutagenesis (A279T) to remove the internal BsaI recognition site in *SPL7* through a silent mutation. A Kozak consensus sequence and N- and C-terminal HA-tag sequences were added to the genomic *SPL7* sequence with primer overhangs by PCR before cloning into the Greengate entry module pGGI000 via BsaI restriction digest. The downstream *SPL7* terminator segment (+1 to +438 from the end of the translational stop codon) was PCR-amplified and cloned into the Greengate entry module pGGE000 via BsaI restriction digest. Using all entry modules and pGGF005 (Lampropoulos et al., 2013), the construct *SPL7prom:HA-SPL7-HA: SPL7term* (*HygR*) was assembled into the Greengate destination vector pGGZ003. The resulting binary plasmid was used to transform Agrobacterium (*Agrobacterium tumefaciens*) (strain GV3130 [pSoup]), and the *spl7-1* mutant was transformed using the floral dip method (Clough and Bent, 1998). All primer sequences used for cloning are listed in Supplemental Table S4.

### Plant growth

Plant cultivation in soil was under an LD photoperiod (16-h light at 145 µmol m^−2^ s^−1^, 22°C/8-h dark, 18°C), with Cu conditions as described (Schulten et al., 2019). Glass petri dishes were soaked in 0.2 N HCl overnight and rinsed with deionized water to remove possible contaminant Cu before autoclaving. For plant cultivation in sterile culture on glass petri dishes, WT or mutant seeds were surface-sterilized by sequential incubation with 70% (v/v) ethanol and 10% (v/v) bleach, stratified in the dark at 4°C for 2 days and sown on a modified Hoagland solution (0.28 mM KH_2_PO_4_, 1.25 mM KNO_3_, 1.5 mM Ca(NO_3_)_2_, 0.75 mM MgSO_4_, 5 µM of a complex of Fe(III) and *N*,*N*′‐di‐(2‐hydroxybenzoyl)‐ethylenediamine‐*N*,*N*′‐diacetate (HBED), 25 µM H_3_BO_3_, 5 µM MnSO_4_, 5 µM ZnSO_4_, 0.5 µM CuSO_4_, 50 µM KCl, and 0.1 µM Na_2_MoO_4_, buffered to pH 5.7 with 3 mM 2‐(*N*‐morpholino)ethanesulfonate) in ultrapure water (Becher et al., 2004, with modifications), containing 1% (w/v) sucrose unless indicated otherwise and solidified with 1% (w/v) agar Type M (Sigma-Aldrich, Steinheim, Germany), which was EDTA-washed prior to its use as described (Schulten et al., 2019). Generally, 20 (or 40 for *spl7-1* under conditions without added CuSO_4_) seedlings were grown on each vertically oriented round glass petri plate (diameter of 150 mm) under an SD photoperiod (8 h light at 145 µmol m^−2^ s^−1^, 22°C/16 h dark, 18°C) in a growth chamber (CFL Plant Climatics, Wertingen, Germany) for 21 days and pooled during harvest. For ChIP and RNA-seq, seedlings were grown for 21 days as described for Cu-deficiency experiments in sterile culture, on glass petri plates in an 11-h light/13-h dark photoperiod (Schulten et al., 2019).

For experiments with combined Cu and sugar treatments, seedlings were pregerminated on modified Hoagland medium without added CuSO_4_, containing 0.5% (w/v) sucrose and solidified with 1% (w/v) unwashed agar Type M (Sigma-Aldrich, Steinheim, Germany), on square polypropylene petri dishes (120 mm × 120 mm) for 7 days. Seedlings were then transferred to controlled Cu growth conditions based on EDTA-washed agar as described above for further cultivation for 14 days, with the addition of either 0.05 µM CuSO_4_ (low Cu) or 0.5 µM CuSO_4_ (control conditions) and no (0%) or 6% (w/v) sucrose. All seedlings cultivated on soil and on agar-solidified medium were harvested at Zeitgeber time 3 (ZT 3) (3 h after lights on), or transferred to darkness for 30 min at ZT 3 for the quantification of ATP and NADH/NAD^+^, unless indicated otherwise.

For liquid cultures, 3.5 mg of surface-sterilized and stratified seeds of WT and *spl7-1* were grown in 50 mL liquid 2 × modified Hoagland solution with 0.5% (w/v) sucrose in 300-mL Erlenmeyer flasks placed on a rotary shaker (80 rpm) under an 8-h light (145 µmol m^−2^ s^−1^, 22°C)/16-h dark (18°C) photoperiod in a growth chamber (CFL Plant Climatics, Wertingen, Germany) for 14 days. Note that all liquid cultures were germinated without added CuSO_4_ for 1 week, after which the medium was exchanged and half of the cultures for each genotype were cultivated with 1 µM CuSO_4_ for the remainder of the growth period. Two days before the sugar starvation treatment (day 13), the medium was exchanged again. The 3-h sugar starvation treatment was started at ZT 1 on day 15: cultures were washed twice with sterile ultrapure water before incubation in fresh solutions without or with 0.5% (w/v) sucrose. For harvest (ZT 4), seedlings were washed in ultrapure water, shoots were separated from roots with a scalpel, and blotted dry before snap-freezing in liquid nitrogen.

### Quantification of plant biomass and elemental concentrations

Quantification of plant biomass and elemental concentrations in plant tissues was conducted as described (Sinclair et al., 2017; Schulten et al., 2019). Aerial tissue samples were homogenized by grinding with a pestle in a mortar that had been soaked in 0.2 N HCl overnight, rinsed in ultrapure water, and dried at 60°C for >1 h beforehand.

### RNA extraction and reverse transcription–quantitative PCR

RNA extraction, cDNA synthesis using oligo(dT)_18_ primers, and reverse transcription–quantitative PCR (RT–qPCR) were performed as described (Schulten et al., 2019). Stem–loop pulsed reverse transcription of mature miRNAs was performed following a published protocol (Varkonyi-Gasic et al., 2007). Relative transcript levels (RTLs) were calculated as follows: RTL = ${\text{RE}}_{m}^{–\Delta \text{CT}}$, with RE_m_ as the mean of reaction efficiencies per primer pair and ΔC_T_ = C_T_(target gene) – C_T_(constitutively expressed reference genes: *EIF4*, *HEL*, or *TUB3*), as described (Bernal et al., 2012). Primer sequences are listed in Supplemental Table S4.

### RNA in situ hybridization

Vegetative-stage shoot apical meristems were harvested from soil-grown plants at 8 days (cultivation in 16-h LDs) and 30 days (cultivation in 8-h SDs) of age, inflorescences from LD grown plants at 15–20 cm height; tissues were processed to conduct RNA in situ hybridization, with probe synthesis from the coding sequence of *SPL7* amplified and cloned into pGEM-Teasy (Promega, Mannheim, Germany) according to manufacturer instructions, as described (Wahl et al., 2013).

### Metabolite extraction and measurement

Anthocyanins were extracted from aliquots (50–100 mg) of frozen ground shoot tissue powder in 2 mL methanol containing 1% (w/v) HCl by shaking overnight on a rotary shaker (150 rpm) at 4°C in the dark. Spectrophotometry was conducted on supernatants in 96-well plates and relative anthocyanin concentrations were calculated as (A_530_ – 0.25*A_657_) g^−1^ fresh biomass (Rabino and Mancinelli, 1986).

Soluble sugars were extracted from aliquots (20 mg) of frozen ground shoot tissue powder in 250 µL of 80% (v/v) ethanol containing 10 mM HEPES/NaOH (pH 7.0) at 80°C for 30 min. After a 10-min centrifugation step at 1,200 × *g* at room temperature, the supernatant was collected and stored on ice. The extraction was repeated in 150 µL of the same solution and in 250 µL of 50% (v/v) ethanol containing 10 mM HEPES/NaOH (pH 7.0). Supernatants were combined and evaporated to dryness using a centrifugal vacuum dryer, followed by resuspension in 250 µL sterile ultrapure water. Aliquots (10–50 µL) were analyzed for sucrose and glucose contents with a K-SUFRG Kit (Megazyme, Bray, Ireland) as triplicate measurements in 96-well plates using a Synergy HTX microplate reader (BioTek, Bad Friedrichshall, Germany). For the quantification of starch, dried residues from the ethanolic extractions were washed in 1 mL ultrapure water, then resuspended in 400 µL 0.1 M NaOH and heated at 98°C for 30 min. After pH neutralization, 100 µL starch degradation mix (16.8 U mL^−1^ amyloglucosidase [#10102857001; Roche, Mannheim, Germany] and 12 U mL^−1^ α-amylase [#10102814001; Roche, Mannheim, Germany] in 50 mM Na-acetate pH 4.9) was added per sample, followed by constant shaking at 37°C overnight. After centrifugation as described above, aliquots (50 µL) were used for the quantification of glucose levels as described above. For starch staining, seedlings were fixed in 80% (v/v) methanol and heated for 5 min at 80°C for chlorophyll removal. Seedlings were incubated in Lugol’s iodine solution for 5 min and washed with deionized water before imaging.

T6P, phosphorylated intermediates, and organic acids were extracted from aliquots (15–20 mg) of frozen ground shoot tissue powder in a chloroform/methanol mixture (3:7, v/v) and evaporated to dryness using a centrifugal vacuum dryer. The dried extracts were dissolved in 350 μL purified water and filtered through MultiScreen PCR-96 Filter Plate membranes (Merck Millipore, Darmstadt, Germany) to remove high-molecular mass compounds. The metabolite extracts were subjected to high‐performance anion‐exchange chromatography coupled to tandem mass spectrometry (LC–MS/MS) as described (Lunn et al., 2006), with modifications (Figueroa et al., 2016).

For the quantification of ATP levels, frozen ground shoot tissue (50 mg) was homogenized in 1 mL phenol (equilibrated with 10 mM Tris–HCl pH 8.0, 1 mM EDTA) and extracted into 0.5 mL of 10 mM Tris–HCl pH 8.0 according to a protocol for maize endosperm (Lappe et al., 2018). After centrifugation at room temperature for 10 min at 10,000 × *g*, the aqueous phase was extracted in an equal volume of chloroform. The supernatant was diluted 200-fold in 10 mM Tris–HCl pH 8.0, and 100 μL sample volumes were assayed in triplicate for ATP contents using a BacTiter-Glo bioluminescence kit (Promega, Mannheim, Germany). After a 5-min incubation in the dark to allow for the decay of plate autofluorescence, photon emissions were recorded in white 96-well plates with an integration time of 0.5 s using a Synergy HTX microplate reader. ATP concentrations were quantified based on a standard curve (50 pM to 1 µM) and normalized to fresh biomass. For the quantification of NAD^+^ and NADH levels, 25-mg aliquots of frozen ground shoot tissues were extracted with 250 µL of either 0.2 M NaOH (NADH) or 0.2 M HCl (NAD^+^). The homogenates were centrifuged at 10,000 × *g* and 4°C for 10 min. The supernatant (200 µL) was neutralized with 175 µL of either 0.2 M HCl or 0.2 M NaOH, and the neutralization to pH 7.0–8.0 was confirmed with pH indicator paper. Aliquots of 50 µL were analyzed in triplicate for NAD^+^ and NADH with a NAD/NADH-Glo Kit (Promega, Mannheim, Germany) in white 96-well plates using a Synergy HTX microplate reader, with normalization to fresh biomass.

### Respiration measurements

The uptake of O_2_ by whole rosettes was quantified using a CG867 O_2_-meter (Schott Instruments, Weilheim, Germany) and a Clark-type oxygen electrode OX1100 (Schott Instruments, Weilheim, Germany), calibrated with saturated sodium dithionite solution and air-saturated water. Measurements were performed in darkness under constant stirring in a cuvette surrounded by a water-flow cooling system. Seedlings were kept in the dark for 30 min prior to the start of measurements to prevent photosynthesis. A total of 100 mg fresh biomass of rosette tissue was submerged in 3 mL of air-saturated 20 mM potassium phosphate buffer (pH 6.8) in the measuring cuvette. After 5 min of equilibration time, total respiration was monitored for 10 min, during which O_2_ concentration was recorded every 30 s. Then, KCN (1 mM final concentration) or SHAM (20 mM final concentration) was added to the cuvette for the measurement of cyanide (CN)- or SHAM-resistant respiration, respectively. Following the addition of an inhibitor and stabilization of the rate of O_2_ uptake during an equilibration time, respiration was monitored for another 7–10 min.

### Immunoblots

Immunological detection of S6K-1/2 and S6K-p was performed as described with some modifications (Dong et al., 2017). In brief, total soluble protein was extracted from 50  mg of ground frozen shoot material in 250  µL of 2 × Laemmli buffer containing 1% (v/v) phosphatase inhibitor cocktail 2 (Sigma-Aldrich, Steinheim, Germany). Proteins were denatured for 10 min at 90°C and separated using sodium dodecyl sulfate polyacrylamide gel electrophoresis (SDS-PAGE) at 10% (w/v) acrylamide (22 mA, 2 h), followed by wet tank transfer to nitrocellulose membranes (100 V, 1 h, 4°C) (Towbin et al., 1979). After Ponceau S staining (0.2% [w/v] Ponceau-S in 1% [v/v] acetic acid) to confirm equal loading per lane and blocking with 5% (w/v) BSA (bovine serum albumine) in Tris-buffered saline containing 0.05% (v/v) Tween-20 (TBST) for 1 h, membranes were incubated with the primary antibody Phospho-p70 S6K (#9205; Cell Signaling, Frankfurt am Main, Germany) or anti-S6K1/2 (#AS12-1855; Agrisera, Vännas, Sweden) diluted 1:5,000 in TBST additionally containing 1% (w/v) BSA (TBSTB) at 4°C overnight. Membranes were washed 3 times for 10 min each in TBST and then incubated with an HRP-conjugated secondary antibody diluted 1:15,000 in TBSTB at room temperature for 1 h. After repeating the washes with TBST, detection was carried out with Pierce ECL Western Blotting Substrate (ThermoFisher, Schwerte, Germany) using a Fusion Fx7 GelDoc (Vilber Lourmat, Eberhardzell, Germany).

For the immunological detection of HA epitope-tagged SPL7, total soluble proteins were extracted from 50 mg of ground frozen shoot material with 100 µL 2 × Laemmli buffer (#S3401, Sigma-Aldrich, Steinheim, Germany). Proteins were denatured and separated as described above, with wet/tank transfer overnight (60 mA, 4°C) (Towbin et al., 1979). After Ponceau S staining of the membrane as described above and blocking with 5% (w/v) blotting-grade nonfat dry milk in TBST for 1 h, membranes were incubated with a primary anti-HA antibody (#26183; ThermoFisher, Schwerte, Germany) diluted 1:5,000 in TBST containing 1% (w/v) nonfat dry milk (TBSTM) or anti-Actin antibody diluted 1:2,500 in TBSTM (#AS132640; Agrisera, Vännas, Sweden) at room temperature for 2 h. Membranes were washed as described above and then incubated with horseradish peroxidase (HRP)-conjugated secondary antibodies (ThermoFisher, Schwerte, Germany) diluted 1:250 (#32430 following anti-HA) in TBSTM or 1:2,500 (#31466, following anti-Actin) in TBSTM at room temperature for 1 h, followed by washing as described above and detection with ECL Select Western Blotting Reagent (GE Healthcare, Little Chalfont, England) as described above.

### Flowering time parameters

The number of days to flowering was scored from the day the seeds were released in the growth chamber after stratification until the bolting shoot reached ∼0.5 cm in height. The number of rosette leaves was counted alongside plant growth until the bolting shoot had reached ∼0.5 cm in length. Plants were photographed 10 days after bolting to record the flowering phenotype. Rosette leaves and aerial tissues above the rosette were then harvested for analysis of elemental contents.

### ChIP and analysis

For one pool of rosette leaves from 80 *spl7-1 HA-SPL7-HA* or WT seedlings grown on petri plates (four replicate plates per pool, three replicate pools were generated per experiment: one for sequencing, one for qPCR, and one as a back-up), chromatin was crosslinked by vacuum-infiltrating seedlings in a solution of 1% (w/v) formaldehyde in phosphate-buffered saline for 5 min, briefly releasing the vacuum, followed by vacuum infiltration for another 10 min (Gendrel et al., 2002). The crosslinking reaction was stopped by adding glycine to a final concentration of 0.125 M and vacuum infiltrating for 5 min. The rosettes were rinsed twice with sterile ultrapure water (Purelab Flex 2; ELGA LabWater, Celle, Germany), blotted dry, and snap-frozen in liquid nitrogen. After grinding the material to a fine powder with a mortar and pestle in liquid nitrogen, nuclei were isolated as described (Moehs et al., 1988), with all extraction buffers containing 1 mM phenylmethylsulfonyl fluoride (PMSF) and a protease inhibitor cocktail (PIC) at a 1:1,000 dilution (P9599, Sigma-Aldrich, Steinheim, Germany). Chromatin was extracted with nuclei lysis buffer (50 mM Tris–HCl pH 8.0, 10 mM EDTA, 1% [w/v] SDS, 1 mM PMSF, PIC) and sonicated using a Bioruptor Pico (Diagenode, Seraing, Belgium) for five cycles (30 s on/30 s off) to achieve an average fragment size of 200 bp. After removing cellular debris by centrifugation (twice for 10 min at 10,000 × *g* at 4°C), the chromatin was diluted 10-fold with ChIP dilution buffer (1.1% [v/v] Triton X-100, 1.2 mM EDTA, 16.7 mM Tris–HCl pH 8.0, 167 mM NaCl) and precleared at 4°C for 1 h by incubation with 80 µL of protein A beads (#17127901, GE Healthcare, Freiburg, Germany), preequilibrated with ChIP dilution buffer. After this preclearing step, an aliquot corresponding to 1% (v/v) of the starting chromatin volume was removed for use as the input DNA control. Monoclonal anti-HA antibody 12CA5 (ROAHA; Roche, Mannheim, Germany) was used to immunoprecipitate HA-SPL7-HA-bound chromatin (5 µg of antibody per IP) with 50 µL of preequilibrated protein A beads at 4°C overnight on a rotary shaker (12 rpm). Washes of the immunocomplexes were performed as follows: one wash step with low salt wash buffer (150 mM NaCl, 0.1% [w/v] SDS, 1% [v/v] Triton X-100, 2 mM EDTA, 20 mM Tris–HCl pH 8.0), two wash steps with high salt wash buffer (500 mM NaCl, 0.1% [w/v] SDS, 1% [v/v] Triton X-100, 2 mM EDTA, 20 mM Tris–HCl pH 8.0), three wash steps with LiCl wash buffer (250 mM LiCl, 0.5% [w/v] IGEPAL CA-630, 0.5% [w/v] sodium deoxycholate, 1 mM EDTA, 10 mM Tris–HCl pH 8.0) and two wash steps with TE buffer (1 mM EDTA, 10 mM Tris–HCl pH 8.0) (Yamaguchi et al., 2014a, with modifications). The immunocomplexes were eluted twice from the protein A beads with freshly prepared elution buffer (1% [w/v] SDS, 0.1 M NaHCO_3_) by incubating at 65°C for 15 min at 1,200 rpm in a ThermoMixer Comfort (Eppendorf AG, Hamburg, Germany). NaCl was added to the eluates and the input DNA aliquots to a final concentration of 0.2 M; samples were then incubated overnight (65°C, 600 rpm) for de-crosslinking and treated with proteinase K at 42°C for 1 h. DNA was purified with a NucleoSpin PCR Cleanup kit (#740609; Macherey-Nagel, Düren, Germany) with buffer NTB (#740595.150; Macherey-Nagel, Düren, Germany) and eluted in 15 µL of 5 mM Tris–HCl pH 8.5.

ChIP samples were tested for enrichment by qPCR, which was performed as described above, measuring enrichment on the promoter regions of *FSD1* and *MIR408* as positive controls and *ACTIN7* as a negative control (Schulten et al., 2019). The sequences of the primers used in ChIP-qPCR are listed in Supplemental Table S4. Libraries for ChIP-seq were prepared and sequenced at the Max-Planck-Genome-Center, Cologne, on an Illumina HiSeq3000 instrument (Romera-Branchat et al., 2020).

### Transcriptome sequencing

WT and *spl7-1* seedlings were grown and harvested as described above for ChIP. Total RNA was extracted from 50-mg aliquots of frozen ground rosette tissues using an RNeasy Plant Mini Kit (#74904, Qiagen, Hilden, Germany) including on-column DNase I digestion to remove any contaminating genomic DNA (#79254, Qiagen, Hilden, Germany). Total RNA was quantified using a Qubit RNA Assay Kit with a Qubit 2.0 Fluorometer (Thermo Fisher, Schwerte, Germany). RNA integrity was assessed with an RNA Nano 6000 Assay Kit of an Agilent Bioanalyzer 2100 system (Agilent Technologies, Waldbronn, Germany); the RNA integrity number was between 8 and 9 for all samples. Library construction and sequencing were performed by Novogene Company (Wan Chai, Hong Kong). In brief, 1 µg of total RNA was used as input for library preparation, mRNA was enriched with oligo(dT)-beads and cDNA was synthesized with random hexamer primers with the NEB Next Ultra RNA Library Prep Kit (NEB, Ipswich, MA, USA). All libraries were sequenced using an Illumina NovaSeq6000 instrument in paired-end mode with a read length of 150 bp.

### ChIP sequence data analysis

Upon sequencing of chromatin immonoprecipitates (IPs) and the corresponding input control libraries of all samples from four independent experiments (addressed as four replicates below), adapter sequences were removed and low-quality ends were trimmed from raw reads using cutadapt (Martin, 2011) and Trimmomatic (Bolger et al., 2014), respectively. Read length distribution was summarized using the density function available in R (R Core Team, 2019). Reads were mapped to the Arabidopsis TAIR10 reference genome assembly using bowtie2 (Langmead et al., 2009; Langmead and Salzberg, 2012) with default settings and a minimum mapping quality of 30, obtaining between 9 and 11 million reads per sample (median 10.4 million reads). ChIP peaks were identified using MACS2 version 2.1.3.3 (Zhang et al., 2008) on each IP together with the respective input from each sample by adjusted manifold confidence of enrichment ratio (-m 2 20; false discovery rate (FDR) ≤ 0.05 (Benjamini and Hochberg, 1995). The peaks of replicates were merged when at least 80% of the length of the shortest genomic segment covered by a peak overlapped with the segment covered by another peak (Romera-Branchat et al., 2020). A merged peak segment was thus at least as long as, or longer than, the broadest of the replicate peaks merged. A peak was considered as present in both low-Cu and control-Cu conditions, or in both the *HA-SPL7-HA* line and the WT, when there was any overlap between the segments defined by peak center (mid-point of segment covered by the merged peak) ± 30% length of segment covered by the merged peak, respectively. Peaks were associated with Arabidopsis genes (TAIR10) using the Bioconductor R package chipPeakAnno and as previously described (Romera-Branchat et al., 2020).

### RNA-seq data analysis

Adapters and low-quality bases were removed from raw reads of the RNA-seq data from three independent experiments using trimmomatic (Bolger et al., 2014), keeping reads of at least 120 bp in length (19.6–27.3 million trimmed reads per sample). Reads were mapped to the Arabidopsis TAIR10 genome assembly using hisat2 version 2.1, excluding unaligned reads from the output BAM file (Lamesch et al., 2012; Berardini et al., 2015; Kim et al., 2019), followed by corrections of multiple mapping with identical start or end positions through COMEX 2.1 (Pietzenuk et al., 2016). Total numbers of counts per gene were retrieved nonstrand specifically using Qualimap version 2.2.1 employing the proportional algorithm for multiply mapping reads and the Arabidopsis Araport11 genome annotation (Okonechnikov et al., 2016; Cheng et al., 2017). Differentially expressed genes were identified using DESeq2 version 3.11 on the R 4.0.3 statistical computing platform with default settings using the local fit type (fitType =  “local”) (Love et al., 2014; R Core Team, 2019) (Supplemental Data Sets 3 and 5).

### Motif discovery

For each set of ChIP peaks (Supplemental Data Set 1) passing a given set of filters (Supplemental Data Sets 3–6), the corresponding genomic sequences were extracted and analyzed for enriched motifs using MEME-ChIP (Bailey et al., 2009; Machanick and Bailey, 2011), with the following parameters: maximum motif width (-meme-maxw) 10, minimum motif width (-meme-minw) 4, motif occurrences mode (-meme-mod), anr (any number of repetitions), and motif database (-db) ArabidopsisDAPv1.meme. The predicted motifs were extracted from the corresponding FIMO (Find Individual Motif Occurrences) output GFF file using a custom-made shell script and subsequently collated with the corresponding set of genes for calculation of motif distance to peak center, motif distance to TSS (gene transcriptional start site), and motif incidence using custom-made scripts (https://github.com/bpietzenuk/spl7_ChIP-RNAseq_KraemerLab).

### Quantification and statistical analyses

Each experiment was performed independently 2 to 3 times. Poorly germinated seedlings or obvious phenotypic outlier individuals were excluded from harvest and measurements. Data are shown from one representative experiment, with *n* as indicated in the figure legends. Statistical analyses were performed with R version 3.5.0 (R Core Team, 2019). Analysis of variance (ANOVA) followed by a Tukey’s post-hoc test (*P* < 0.05) was conducted for datasets with homoscedasticity and a normal distribution of residuals. For all other datasets, pairwise *t* tests (Student’s *t* test or Welch *t* test as applicable) with false discovery rate adjustment (*q*-value < 0.05) were performed instead (Storey et al., 2019).

### Accession numbers

Sequence data from this article can be found at ENA, EMBL-EBI under accession number PRJEB47134 (ERR6548266–ERR6548277 for RNA-seq, ERR6558497–ERR6558513 for ChIP-seq). Graph bed files for called peaks per sample are provided in https://github.com/bpietzenuk/spl7_ChIP-RNAseq_KraemerLab. AGI codes are as follows (see also Supplemental Table S5): At5g18830.1 (*SPL7*), At4g25100 (*FSD1*), At1g71200 (*bHLH160*/*CITF1*), At4g28790 (*bHLH23*/*CITF2*), At5g59520 (*ZIP2*), At5g24380 (*YSL2*), At5g44790 (*RAN1*/*HMA7*), At2g28190 (*CSD2*), At3g46900 (*COPT2*), At1g32350 (*AOX1D*), At5g23980 (*FRO4*), At5g23990 (*FRO5*), At4g33520 (*PAA1*/*HMA6*), At3g47340 (*DIN6*), At1g26770 (*EXP10*), At1g78580 (*TPS1*), MIR156, At2g47015 (MIR408), At2g47070 (*SPL1*), At3g15270 (*SPL5*), At2g42200 (*SPL9*), At5g50570/At5g50670 (*SPL13A*/*B*), At1g20980 (*SPL14*), At3g57920 (*SPL15*), At5g14545 (*MIR398B*), At5g14565 (*MIR398C*), At4g13555 (*MIR397B*), At4g13554 (*MIR857a)*, At3g23326 (*MIR853a)*, At5g26038 (*MIR860a*), At1g19371 (*MIR169H*), At5g60390 (*EF1α*), At3g13920 (*EIF4A1*), At1g58050 (*HEL*)*.* At4g05320 (*UBQ10*), At5g62700 (*TUB3*), and At5g09810 (*ACT7*).

## Supplemental data

The following materials are available in the online version of this article.

**Supplemental Table S1.** Metabolite data.

**Supplemental Table S2.** Metabolite data for the WT, *spl7-1*, *spl7-2*, and *spl7-2 SPL7* (*spl7-2*_C).

**Supplemental Table S3.** Number of genes commonly identified in this study and in earlier studies.

**Supplemental Table S4.** Oligonucleotides used in this study.

**Supplemental Table S5.** List of locus identifiers for genes mentioned in this article.

**Supplemental Figure S1.** Effects of Cu deficiency and sucrose on nutrient metal concentrations in WT and *spl7-1* mutant seedlings.

**Supplemental Figure S2.** Sucrose does not stimulate shoot fresh biomass gain in *spl7* mutants cultivated under low-Cu conditions.

**Supplemental Figure S3.** Independent experiment (repeat) related to Figure 1, C–E.

**Supplemental Figure S4.** Effects of Cu deficiency and sucrose on relative transcript levels of known SPL7-dependently expressed genes.

**Supplemental Figure S5.** Starch levels in WT and *spl7* mutant seedlings upon cultivation in solid agar media containing different combinations of Cu and sucrose.

**Supplemental Figure S6.** Mitochondrial electron transfer chain and independent experiments (repeats) related to Figure 2.

**Supplemental Figure S7.** Two independent experiments (repeats) related to Figure 4.

**Supplemental Figure S8.** Two independent experiments (repeats) related to Figure 5, D and E.

**Supplemental Figure S9.** Complementation of the *spl7-1* mutant by the *SPL7prom:HA-SPL7-HA:SPL7term* transgene (two independent lines in addition to line 4-1 shown in Figure 6).

**Supplemental Figure S10.** Full image and independent replication of immunoblot shown in Figure 6C.

**Supplemental Figure S11.** Distribution of genomic SPL7 binding sites relative to genes.

**Supplemental Figure S12.** SPL7 binding profiles at chosen loci, and two independent repeats related to Figure 7C.

**Supplemental Figure S13.** Enriched motifs identified by MEME motif analysis among SPL7 binding sites detected exclusively under low Cu.

**Supplemental Figure S14.** Enriched motifs identified by MEME motif analysis on subgroups of SPL7 binding sites delineated by including information on gene expression.

**Supplemental Figure S15.** Putative SPL7-binding motifs at the *Fe SUPEROXIDE DISMUTASE1* (*FSD1*) locus identified by ChIP-seq.

**Supplemental Figure S16.** GO and KEGG enrichment analyses.

**Supplemental Data Set 1.** Genomic binding sites of SPL7 detected by ChIP-seq.

**Supplemental Data Set 2.** Genes associated with SPL7-binding sites detected by ChIP-seq, and comparison with published DAP-seq data for other Arabidopsis SPL proteins.

**Supplemental Data Set 3.** Universal RNA-seq data.

**Supplemental Data Set 4.** Motifs in genomic SPL7 binding sites identified by ChIP-seq.

**Supplemental Data Set 5.** Genes exhibiting both *SPL7*-dependent regulation of transcript abundance and SPL7-binding peaks identified by ChIP-seq.

**Supplemental Data Set 6.** Motifs identified in genes exhibiting different combinations of SPL7-dependent transcriptional regulation and SPL7-binding sites identified by ChIPseq.

**Supplemental Data Set 7.** Statistics summary.

## Supplementary Material

koac215_Supplementary_Data

## Notes

A.S., J.Q., M.S., M.R.B., R.F., and V.W. performed experiments, A.S., B.P., M.K., M.S. E.S., G.C., and U.K. conducted computational or other data analysis, A.S. and U.K. designed the research and wrote the manuscript, with contributions from J.Q. and B.P., all authors edited the manuscript.

The author responsible for distribution of materials integral to the findings presented in this article in accordance with the policy described in the Instructions for Authors (https://academic.oup.com/plcell) is: Ute Krämer (Ute.Kraemer@ruhr-uni-bochum.de).
