# Supplementary material for: Energy status-promoted growth and development of Arabidopsis require copper deficiency response transcriptional regulator SPL7
Source: Plant Cell. 2022 Jul 22;34(10):3873–98. doi: 10.1093/plcell/koac215 (PMC9516184; doi:10.1093/plcell/koac215)
Supplement: koac215_Supplementary_Data [file koac215_supplementary_data.zip › koac215_Supplementary_Data/Schulten_et_al_Suppl_Data_corrected_Tables_first.pdf]

Supplemental Table S1. Metabolite data.

	**Experiment 1 (n = 4)**							
	WT				*spl7-1*			
	low Cu		control		low Cu		control	
	Mean	Stdev	Mean	Stdev	Mean	Stdev	Mean	Stdev
Suc6P	0.14	0.02	0.16	0.06	0.34	0.08	0.17	0.01
Tre6P	0.06	0.01	0.07	0.01	0.12	0.03	0.07	0.02
Glc6P	105	13	104	9	120	12	100	14
Man6P	0.8	0.3	1.3	0.3	2.0	1.1	0.8	0.2
GBP	1.28	0.09	1.29	0.21	1.41	0.16	1.25	0.16
Glc1P	7.4	0.7	7.3	0.5	9.3	1.0	7.5	0.7
Gal1P	1.68	0.16	1.78	0.23	2.00	0.31	1.89	0.16
UDPGlc	72.0	8.5	77.5	10.0	78.7	10.4	70.0	5.5
PEP	22.8	4.6	29.3	7.9	17.8	4.3	19.5	7.8
Pyruvate	70	32	69	6	127	23	71	29
Gly3P	10.5	0.8	12.1	1.7	20.3	5.6	13.4	1.5
2-OG	29.08	9.53	42.15	14.06	15.29	2.89	39.88	14.05
Succinate	86	14	103	11	178	55	108	12
Fumarate	3977	912.74	3570	2414.33	4077	1148	3759	1435
Citrate	2617	395	2808	414	3061	704	3096	328
Aconitate	76.8	11.2	82.0	5.0	86.8	19.9	88.2	6.9
Shikimate	12.1	3.4	13.6	2.4	8.7	2.1	13.0	2.6

	**Experiment 2 (n = 4)**															
	WT				*spl7-1*				*amiRFRO4/5*				*paa1-3*			
	low Cu		control		low Cu		control		low Cu		control		low Cu		control	
	Mean	Stdev	Mean	Stdev	Mean	Stdev	Mean	Stdev	Mean	Stdev	Mean	Stdev	Mean	Stdev	Mean	Stdev
Suc6P	0.17	0.05	0.15	0.03	0.31	0.08	0.24	0.07	0.23	0.10	0.24	0.04	0.12	0.01	0.17	0.02
Tre6P	0.08	0.01	0.08	0.01	0.12	0.04	0.13	0.03	0.09	0.02	0.11	0.02	0.02	0.00	0.06	0.01
Glc6P	112	9	104	7	122	18	106	6	130	14	120	27	84	8	108	12
Man6P	3.7	2.0	3.6	1.8	4.3	1.1	2.9	1.4	4.2	1.9	3.6	2.0	2.6	1.5	4.4	1.5
GBP	1.61	0.17	1.57	0.12	1.95	0.30	1.94	0.12	1.95	0.13	1.86	0.12	1.66	0.13	1.77	0.16
Glc1P	8.2	0.7	7.6	0.6	9.7	1.9	8.9	0.5	9.2	0.8	9.9	0.4	7.4	0.3	8.5	0.3
Gal1P	1.78	0.16	1.82	0.20	2.11	0.47	2.12	0.21	1.99	0.16	2.14	0.05	1.48	0.13	1.93	0.10
UDPGlc	81.0	9.9	73.4	9.5	79.4	11.1	83.0	7.2	86.6	8.7	89.7	16.7	73.2	6.1	86.2	4.0
PEP	23.2	7.1	15.5	4.4	21.4	4.4	7.4	3.0	17.4	1.9	6.4	4.8	14.2	3.3	9.5	3.1
Pyruvate	78	31	85	33	77	21	100	23	86	8	101	22	45	13	85	27
Gly3P	11.8	1.2	11.7	0.9	18.6	6.2	12.7	1.7	13.6	1.6	13.4	1.9	10.5	0.7	12.3	0.8
2-OG	85.99	43.69	106.60	77.94	33.92	3.30	74.19	23.12	104.97	27.28	110.30	64.49	27.05	11.08	67.79	23.65
Succinate	175	29	143.	21	291	114	211	53	213	53	232	17	32	11	119	24
Fumarate	6088	80	5692	483	5225	980	6830	1433	5294	918	5459	727	1520	353	3571	491
Citrate	2887	258	3080	295	2805	852	4358	733	36212	888	4065	855	1497	299.48	2935	242
Aconitate	66.6	12.5	74.2	14.0	51.0	13.2	89.2	18.1	70.5	23.0	65.4	15.0	29.6	6.4	60.2	10.9
Shikimate	13.3	1.4	15.0	0.6	5.8	1.2	15.7	4.0	14.8	2.6	16.4	2.7	4.2	0.8	10.7	1.2

	**Experiment 3 (n = 6)**															
	WT				*spl7-1*				*amiRFRO4/5*				*paa1-3*			
	low Cu		control		low Cu		control		low Cu		control		low Cu		control	
	Mean	Stdev	Mean	Stdev	Mean	Stdev	Mean	Stdev	Mean	Stdev	Mean	Stdev	Mean	Stdev	Mean	Stdev
Suc6P	0.15	0.04	0.16	0.05	0.29	0.08	0.21	0.04	0.17	0.03	0.18	0.03	0.13	0.02	0.16	0.03
Tre6P	0.09	0.01	0.09	0.03	0.13	0.04	0.13	0.01	0.10	0.01	0.11	0.01	0.04	0.00	0.08	0.01
Glc6P	146	34	152	53	126	32	143	26	137	18	125	13	120	27	134	26
Man6P	17.1	3.5	16.8	5.0	19.3	5.0	18.4	3.3	18.3	2.1	18.4	1.3	14.4	1.7	16.2	1.5
GBP	1.56	0.25	1.63	0.40	1.67	0.30	1.79	0.37	1.74	0.24	1.54	0.11	1.75	0.31	1.55	0.21
Glc1P	9.8	1.3	9.4	2.9	10.7	1.7	10.3	1.8	10.1	0.9	10.0	0.7	9.3	1.0	9.8	0.8
Gal1P	2.55	0.42	2.43	0.65	2.92	0.52	2.81	0.42	2.49	0.22	2.55	0.22	2.45	0.21	2.42	0.19
UDPGlc	58.9	9.8	54.4	13.7	54.4	7.1	61.7	11.5	56.9	4.1	55.3	2.00	57.0	7.7	54.8	4.7
PEP	18.3	8.9	16.7	1.8	22.9	9.0	13.8	6.8	16.3	5.5	9.7	6.5	27.1	3.9	12.0	3.7
Pyruvate	68	25	62	17	87	40	120	38	108	24	135	37	123	30	94	17
Gly3P	12.0	1.7	11.6	2.9	19.9	5.3	14.1	2.2	12.9	2.0	12.9	0.78	12.1	1.1	11.6	0.9
2-OG	249.05	92.75	219.73	60.06	179.89	43.68	330.90	95.15	299.05	47.93	337.27	38.98	176.98	28.85	199.13	21.68
Succinate	103	15	118	47	204	77	131	20	12	29	131	24	24	7	71	18
Fumarate	22541	4556	21698	8118	38736	12481	41313	20793	20301	3388	21270	7525	2883	814	8646	3624
Citrate	3290	455.27	3341	875	3032	711	4212	721	3656	604	3911	561	1728	268	2762	319
Aconitate	56.9	8.6	53.9	12.4	42.4	9.4	66.7	13.3	54.1	3.8	54.4	7.2	39.8	7.2	49.7	2.9
Shikimate	17.8	1.3	18.8	5.0	9.6	1.4	19.6	4.4	19.5	3.3	19.7	3.0	9.4	2.8	14.4	1.4

Given in [nmol g^{-1} FW].
(related to Figure 2).

Supplemental Table S2. Metabolite data for the WT, *spl7-1*, *spl7-2*, and *spl7-2 SPL7* (*spl7-2*_C).

	**Experiment 4 (*n* = 4)**															
	WT				*spl7-2*_C				*spl7-1*				*spl7-2*			
	low Cu		control		low Cu		control		low Cu		control		low Cu		control	
	Mean	Stdev	Mean	Stdev	Mean	Stdev	Mean	Stdev	Mean	Stdev	Mean	Stdev	Mean	Stdev	Mean	Stdev
Suc6P	0.23	0.02	-	-	0.17	0.03	-	-	0.31	0.05	-	-	0.33	0.04	-	-
Tre6P	0.13	0.01	-	-	0.10	0.02	-	-	0.16	0.01	-	-	0.18	0.04	-	-
Glc6P	117	25	-	-	98	17	-	-	104	27	-	-	124	17	-	-
Man6P	13	2	-	-	12	2	-	-	12	2	-	-	15	2	-	-
GBP	1.0	0.3	-	-	1.0	0.1	-	-	0.9	0.2	-	-	1.0	0.2	-	-
Glc1P	9.8	1.9	-	-	8.5	1.0	-	-	9.5	1.5	-	-	10.9	1.5	-	-
Gal1P	2.2	0.3	-	-	2.0	0.3	-	-	2.1	0.5	-	-	2.5	0.4	-	-
UDPGlc	56	7	-	-	49	7	-	-	45	10	-	-	50	7	-	-
PEP	19	5	-	-	23	3	-	-	20	4	-	-	20	8	-	-
Pyruvate	90	20	-	-	69	25	-	-	80	21	-	-	82	14	-	-
Gly3P	14	1	-	-	13	1	-	-	14	2	-	-	15	1	-	-
2-OG	312	44	-	-	404	122	-	-	231	72	-	-	252	31	-	-
Succinate	217	2	-	-	189	36	-	-	337	26	-	-	373	57	-	-
Fumarate	10317	2719	-	-	9370	2882	-	-	11970	885	-	-	11984	1416	-	-
Citrate	4870	654	-	-	4121	445	-	-	2873	254	-	-	3378	796	-	-
Aconitate	89	13	-	-	78	7	-	-	53	6	-	-	56	12	-	-
Shikimate	20	3	-	-	17	1	-	-	12	2	-	-	13	1	-	-

Given in [nmol g^{-1} FW].
(related to Figure 2).

Supplemental Table S3. Number of genes commonly identified in this study and in earlier studies.

	Total no. of genes identified	Total number of genes shared with this study (Low Cu: 2,026 genes)	Total number of genes shared with this study (Control Cu: 1,901 genes)
FLAG-SPL7 ChIP-seq[1]	1,266	185 (2.0×10^{-21})	163 (2.6×10^{-16})
Common to FLAG-SPL7 ChIP-seq and HY5 ChIP-chip[1]	586	90 (3.5×10^{-12})	83 (6.5×10^{-11})
SPL1 DAP-seq[2]	3,731	231 (n.s.)	207 (n.s.)
SPL5 DAP-seq[2]	4,301	264 (n.s.)	242 (n.s.)
SPL9 DAP-seq[2]	13,831	809 (n.s.)	755 (n.s.)
SPL13 DAP-seq[2]	1,061	83 (n.s.)	72 (n.s.)
SPL14 DAP-seq[2]	2,263	167 (n.s.)	146 (n.s.)
Common to all DAP-seq[2]	672	55 (n.s.)	50 (n.s.)
SPL15 DAP amp.2	13,631	780 (n.s.)	725 (n.s.)

P-values of Hypergeometric tests for the number of common loci exceeding the number expected by chance are given in parentheses. n.s.: not significant ($P > 0.1$; total no. of loci 28,497). [1]Zhang et al., 2014; [2]O'Malley et al., 2016.

Supplemental Table S4. Oligonucleotides used in this study.

Oligo name	Oligo sequence (5' → 3')
Oligos used for genotyping	
spl7-1_geno_for	TTGGAAATTCAAGCTGATTCG
spl7-1_geno_rev	TCCACCTGTCAAAACCAAGAC
Oligos used for RT-qPCR and ChIP-qPCR	
AtCOPT2_qRT_f	CATTCACCGATCCTACGTGTCA
AtCOPT2_qRT_r	CACCAAATACGAAAGGCCAGTC
AtCSD2_qRT_f	GATGGCGTGGCAGAAACAA
AtCSD2_qRT_r	AGCTCGTGAACCACAAAGGCT
AtDIN6_qRT_f	TGATGTCGAACGCGGGGCAT
AtDIN6_qRT_r	CCACGGTGGCACCTCCAGGA
AtEF1α_qRT_f	TGAGCACGCTCTTCTTG
AtEF1α_qRT_r	GGTGGTGGCATCCATCT
AtEIF4_qRT_f	TCATAGATCTGGTCCTTGAAACC
AtEIF4_qRT_f	GGCAGTCTCTTCGTGCTGAC
AtEXP10_qRT_f	TCATGGCTTCTTCTGTGAGC
AtEXP10_qRT_r	ACAAGCACCACCCATTGTG
AtFSD1_qRT_f	TCGGCTCTTTCCATTGCTT
AtFSD1_qRT_r	TGGTCTTCGGTTCTGGAAGTCA
AtFT_qRT_f	CGAGTAACGAACGGTGATGA
AtFT_qRT_r	CGCATCACACACTATATAAGTAAAACA
AtHEL_qRT_f	CCATTCTACTTTTTGGCGGCT
AtHEL_qRT_r	TCAATGGTAACTGATCCACTCTGATG
AtpriMIR398B_f	CACGAGTAATCAACGGCTGTAATG
AtpriMIR398B_r	TGAGTAAAAGCCAGCCTTGATAAAAG
AtSPL7_qRT_f	GCATTCAGGTCCCGTCAAACTA
AtSPL7_qRT_r	TGGATGGTAGAGAACCGCACAG
AtUBQ10_f	GGCCTTGTATAATCCCTGATGAATAAG
AtUBQ10_r	AAAGAGATAACAGGAACGGAAACATAGT
At_pFSD1_qRT_f	AGTAGCCACGAAGCCAGAAA
At_pFSD1_qRT_r	TGGTTGGATGGCTCTGCTTT
At_pMIR408_qRT_f	TACGCTTTAGCCAGATCGCA
At_pMIR408_qRT_r	GGAATCGTGTTTCTGTACGCC
At_pACT7_qRT_f	CGTTTCGCTTTCCTTAGTGTTAGCT
At_pACT7_qRT_r	AGCGAACGGATCTAGAGACTCACCTTG
At_pCITF1_f	TGCAGTACAAATGGCGCTAA
At_pCITF1_r	AGAGAGAGGAGAGATTACGGGT
At_pbHLH23_f	ACGTACCAGTTTGCTCGTACA
AtpbHLH23_r	CATCACTCACCACGAAGGCT

Supplemental Table S4 (continued). Oligonucleotides used in this study.

Oligo name	Oligo sequence (5' → 3')
Oligos used for RT-qPCR (miRNA)	
AtmiR156_RT	GTCGTATCCAGTGCAGGGTCCGAGGTATTCGCACTGGATACGA CGTGCTC
AtmiR156_f	GCGGCGGTGACAGAAGAGAGT
AtTUB3_RT	GTGCAGGGTCCGAGGTCCTCAGACAGCAAGTCACAC
AtTUB3_f	TACCCCCAGCTTTGGTGATTTG
miRNA_universal_r	GTGCAGGGTCCGAGGT
Oligos used for cloning	
AtSPL7_1cs_F	ATGTCTTCTCTGTCGCAATCG
AtSPL7_2406cs_R	TCAAATTTTGTGTACCAATCTCATTC
AtSPL7_A279T_for	GGAAGCGTGACCCGAGGTTGATTTGTTC
AtSPL7_A279T_rev	GGTCACGCTTCCTAACCCGATCCG
AtSPL7_-2506p_GGA_for	AACAGGTCTCAACCTGACGCGAAGAATGCTACA
AtSPL7_-5p_GGB_rev	AACAGGTCTCATGTTGAGTCTTCAATTTCTGATAAAATTC
AtSPL7_+HA_GGB_for	AACAGGTCTCAAACAATGGCTTATCCATACGACGTTCCAGATTA TGCATCTTCTCTGTCGCAATCG
AtSPL7_+HA_GGE_rev	AACAGGTCTCAGCAGTCAAGCGTAATCAGGCACATCATAAGGG TAAATTTTGTGTACCAATCTCATTC
AtSPL7_+1t_GGE_for	AACAGGTCTCACTGCTCAGATGAGGTCTTGCTCTTTC
AtSPL7_+438t_GGF_rev	AACAGGTCTCATAGTCTCAATCCAACATGCATCTTAATAC

Supplemental Table S5. List of locus identifiers for genes mentioned in this article.

Gene name	Locus identifier	Gene name	Locus identifier
ACT7	AT5G09810	*MIR408*	At2g47015
AOX1D	AT1G32350	*NRT2.1*	AT1G08090
bHLH100	AT2G41240	*PAA1*	AT4G33520
bHLH101	AT5G04150	*PAA2*	AT5G21930
bHLH23 (CITF2)	AT4G28790	*PC1/PETE1*	AT1G76100
bHLH38	AT3G56970	*PC2/PETE2*	AT1G20340
bHLH39	AT3G56980	*RAN1/HMA7*	AT5G44790
CA2	AT5G14740	*RGS1*	AT3G26090
CITF1	AT1G71200	*SPL1*	AT2G47070
CSD1	AT1G08830	*SPL10*	AT1G27370
CSD2	AT2G28190	*SPL11*	AT1G27360
CTR1	AT5G03730	*SPL12*	AT3G60030
DIN6	AT3G47340	*SPL13A*	AT5G50570
EIF4	AT3G13920	*SPL13B*	AT5G50670
EXP10	AT1G26770	*SPL14*	AT1G20980
FRO4	AT5G23980	*SPL15*	AT3G57920
FRO5	AT5G23990	*SPL16*	At1g76580
FSD1	AT4G25100	*SPL2*	AT5G43270
FT	AT1G65480	*SPL3*	AT2G33810
HCC1	AT3G08950	*SPL4*	AT1G53160
HXK1	AT4G29130	*SPL5*	AT3G15270
HY5	AT5G11260	*SPL6*	AT1G69170
KIN17	AT1G55460	*SPL7*	AT5G18830
LHCB1.1	AT1G29920	*SPL8*	AT1G02065
MIR172C	AT3G11435	*SPL9*	AT2G42200
MIR172D	AT3G55512	*YSL2*	AT5G24380
MIR398B	AT5G14545	*ZIP2*	AT5G59520
MIR398C	AT5G14565	*CRR1*	Cre09.g390023.t1.1

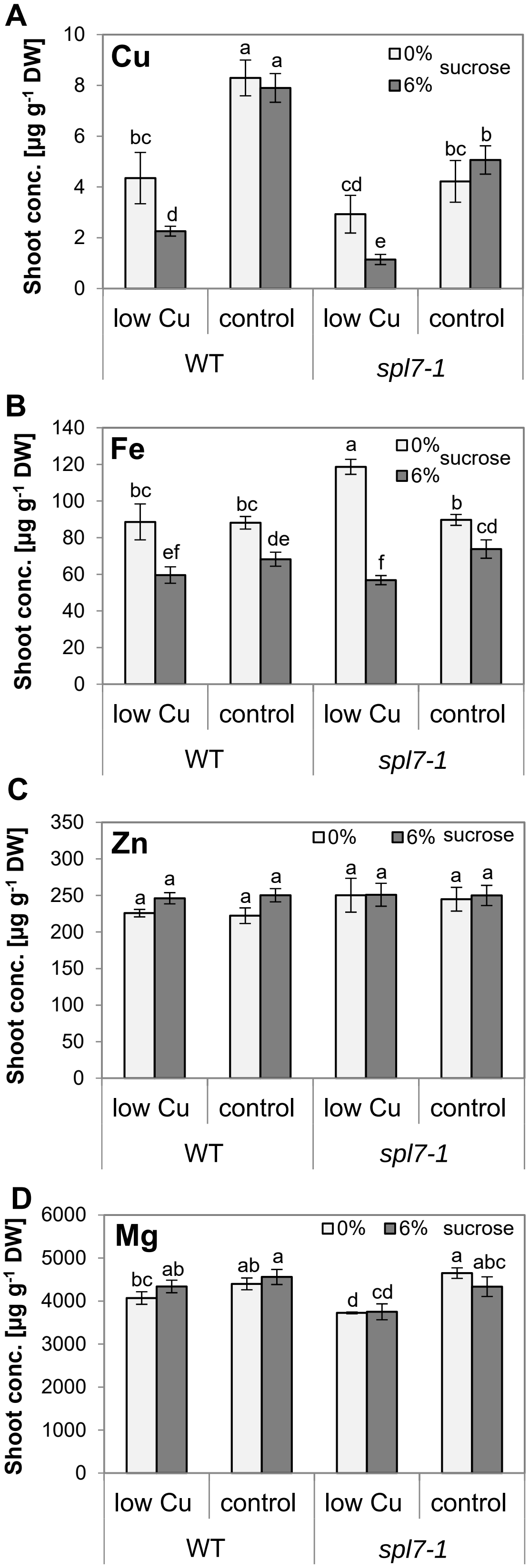

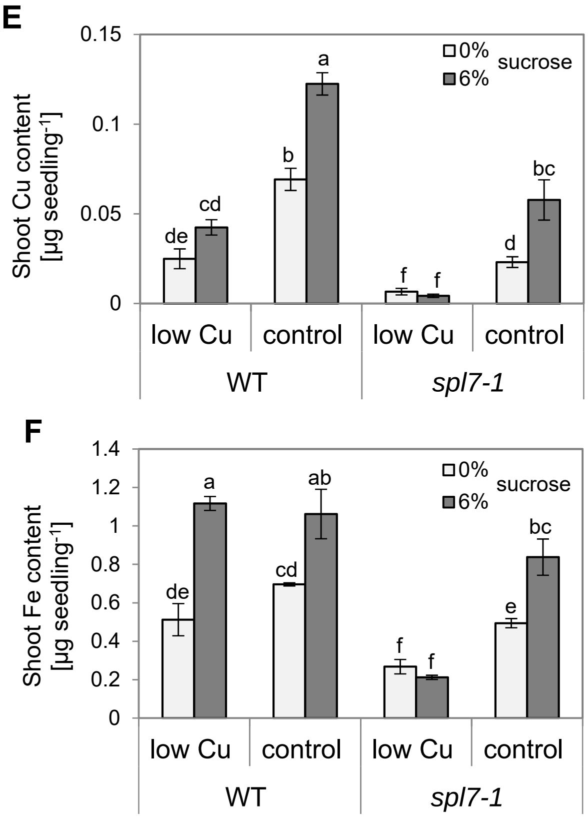

Supplemental Figure S1. Effects of Cu deficiency and sucrose on nutrient metal concentrations in wild-type and *spl7-1* mutant seedlings.

(A-D) Concentrations of Cu (**A**), Fe (**B**), Zn (**C**) and Mg (**D**) in shoots of 21-d-old seedlings cultivated in vertically oriented glass petri plates on low-Cu (0.05 µM $CuSO_4$) or control (0.5 µM $CuSO_4$) EDTA-washed agar solid medium with no added sucrose (0%) or supplemented with 6% (w/v) sucrose for 14 d. **(E, F)** Total shoot content of Cu (**A**) and Fe (**B**) in seedlings cultivated as described for (A-D).

Data are means ± SD (*n* = 3 replicate pools, each from one petri plate). Different lowercase letters denote significant differences between means (*t*-tests with FDR adjustment; *q*-value < 0.05).

(related to Figure 1).

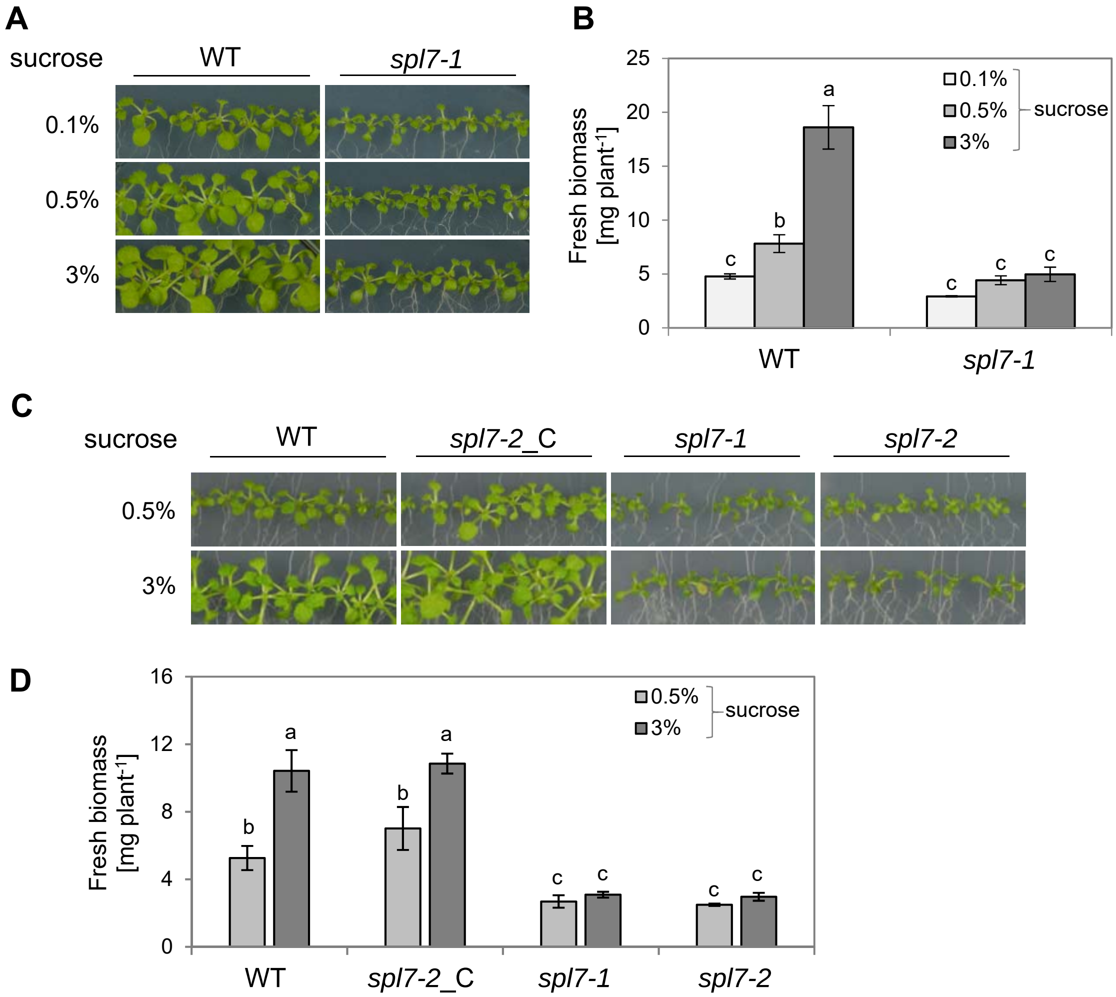

Supplemental Figure S2. Sucrose does not stimulate shoot fresh biomass gain in *spl7* mutants cultivated under low-Cu conditions.

(A-D) Photographs (**A, C**) and shoot fresh biomass (**B, D**) of 21-d-old seedlings cultivated in vertically oriented glass petri plates on low-Cu (0.05 µM $CuSO_4$) EDTA-washed agar solid medium supplemented with 0.1%, 0.5% or 3% (w/v) sucrose. Data are means ± SD (n = 3 pools of seedlings, each from a replicate plate). Different lowercase letters denote significant differences ($P < 0.05$) between means based on ANOVA (Tukey's HSD). *spl7-2*_C: *spl7-2 SPL7* complemented line (Bernal et al., 2012).

(related to Figure 1).

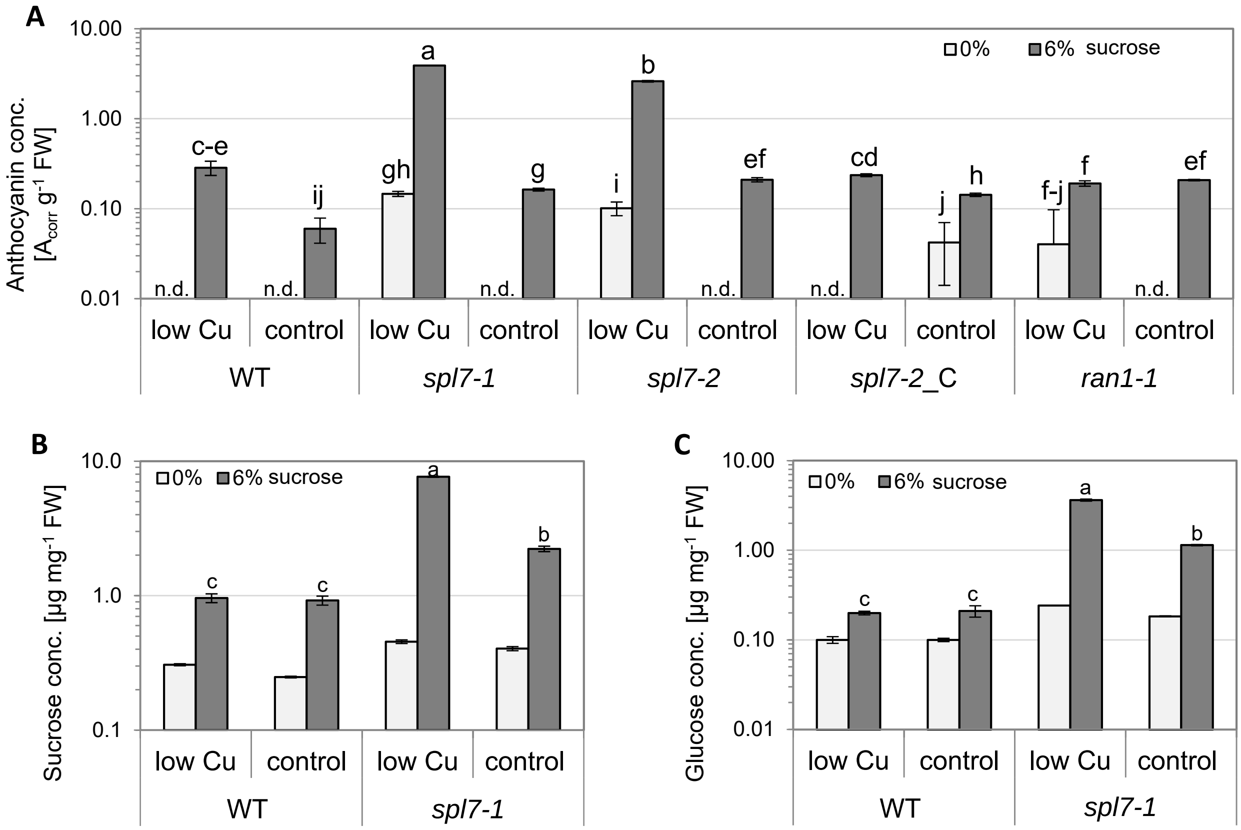

Supplemental Figure S3. Independent experiment (repeat) related to Figure 1, C-E.
(A) Anthocyanin levels in shoots of 21-d-old seedlings cultivated in vertically oriented glass petri plates on low-Cu (0.05 µM $CuSO_4$) or control (0.5 µM $CuSO_4$) EDTA-washed agar solid medium with no added sucrose (0%) or supplemented with 6% (w/v) sucrose for 14 d. Shown are data for the wild type (WT), *spl7-1*, *spl7-2*, an *spl7-2 SPL7* complemented line (*spl7-2*_C; Bernal et al., 2012), and the *ran1-1* mutant for comparison. Data are means ± SD (*n* = 3 technical replicates). **(B, C)** Sucrose (**B**) and glucose (**C**) concentrations in shoots of wild-type and *spl7-1* cultivated as in (A). Data are means ± SD (*n* = 2 and 3 technical replicates for 0% and 6% sucrose condition, respectively). Technical replicates (A-C) are replicate measurements conducted per extract from a pool of 20 or 40 shoots of seedlings cultivated on one plate per genotype and treatment; see Methods). Different lowercase letters denote significant differences between means (*t*-tests with FDR adjustment; *q*-value < 0.05), wherever *n* > 2.
(related to Figure 1).

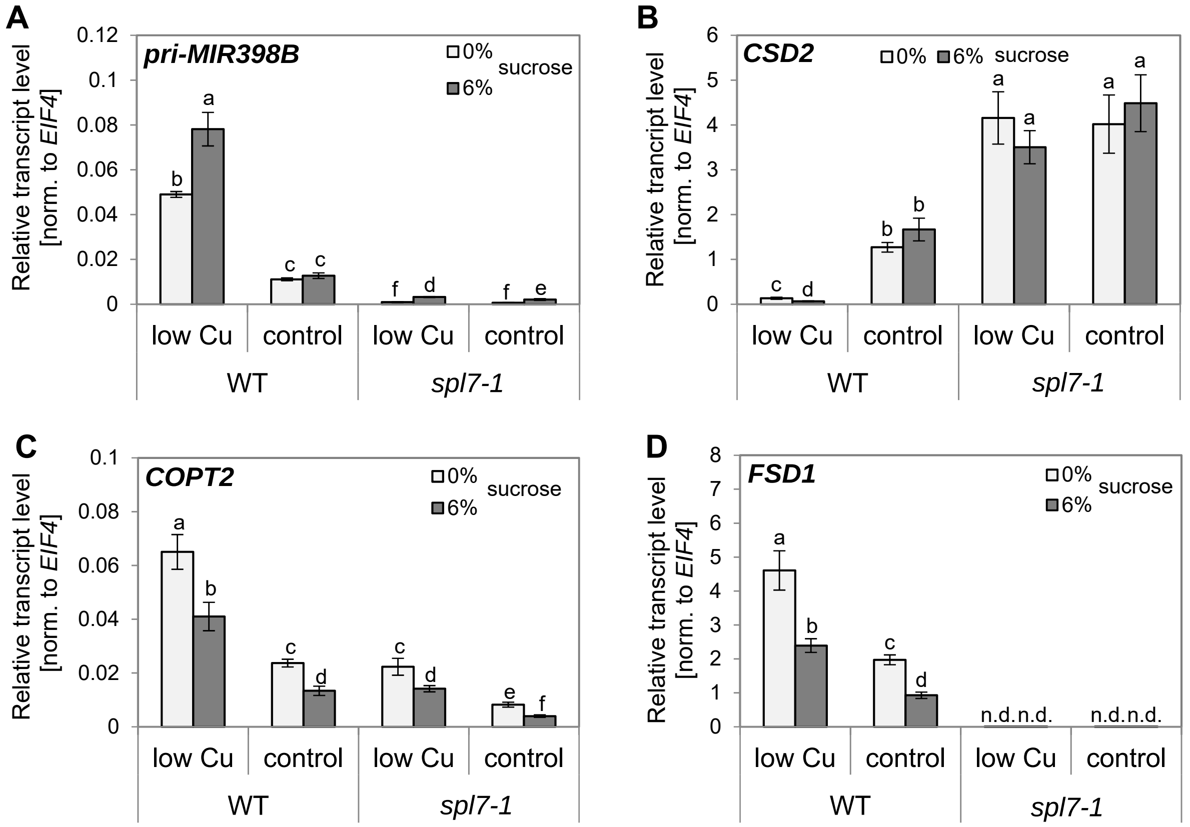

Supplemental Figure S4. Effects of Cu deficiency and sucrose on relative transcript levels of known SPL7-dependently expressed genes.

(A-D) Relative transcript abundance, measured by RT-qPCR, of *pri-MIR398B* (**A**), *CSD2* (**B**), *COPT2* (**C**) and *FSD1* (**D**) in shoots of 21-d-old seedlings. Seedlings were cultivated for 14 d in vertically oriented glass petri plates on low-Cu (0.05 µM $CuSO_4$) or control (0.5 µM $CuSO_4$) EDTA-washed agar solid medium with no added sucrose (0%) or supplemented with 6% (w/v) sucrose. Data are means ± SD (*n* = 3 technical replicates, i.e. independent PCR runs, each with three replicate wells per transcript). Different lowercase letters denote significant differences between means (*t*-tests with FDR adjustment; *q*-value < 0.05).

(related to Figure 1).

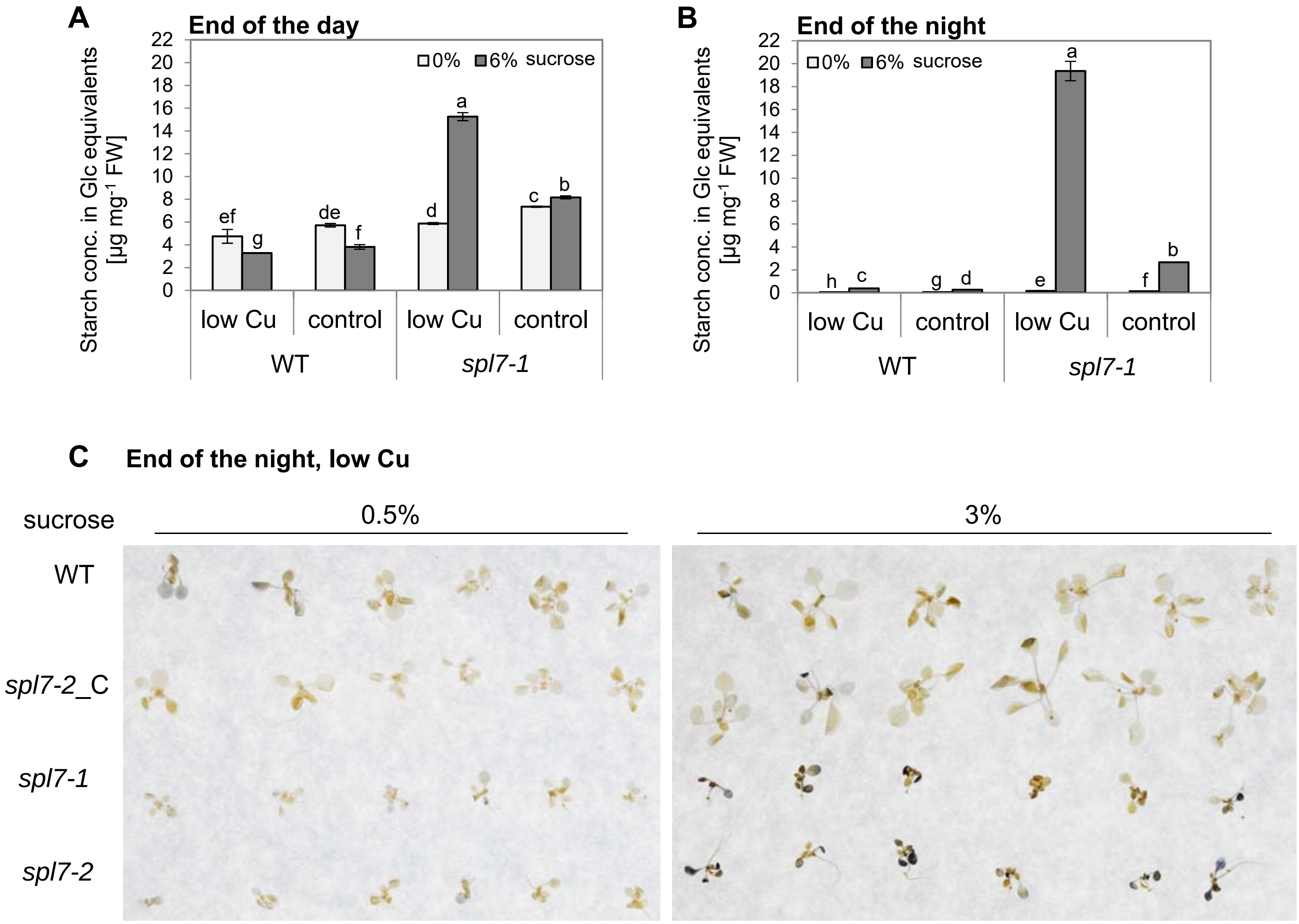

Supplemental Figure S5. Starch levels in WT and *spl7* mutant seedlings upon cultivation in solid agar media containing different combinations of Cu and sucrose .
(A, B) Starch was quantified at the end of the day (**A**) and at the end of the night (**B**) as glucose equivalents in 21-d-old seedlings cultivated for 14 d in vertically oriented glass petri plates on low-Cu (0.05 µM $CuSO_4$) or control (0.5 µM $CuSO_4$) EDTA-washed agar solid medium with no added sucrose (0%) or supplemented with 6% (w/v) sucrose. Seedlings were harvested at the end of the day at ZT 7.5 (**A**) and at the end of the night at ZT 23.5 (**B**). Data are means ± SD (n = 3 technical replicates). Different lowercase letters denote significant differences between means based on t-tests with FDR adjustment (q-value < 0.05, A) and ANOVA (Tukey's HSD; P < 0.05, B). **(C)** Starch staining in 21-d-old seedlings cultivated in vertically-oriented glass petri plates on low-Cu (0.05 µM $CuSO_4$) EDTA-washed agar solid medium supplemented with 0.5% or 3% (w/v) sucrose. Seedlings were harvested at the end of the night at ZT 23.5. *spl7-2*_C: *spl7-2 SPL7* complemented line (Bernal et al., 2012).
(related to Figure 1).

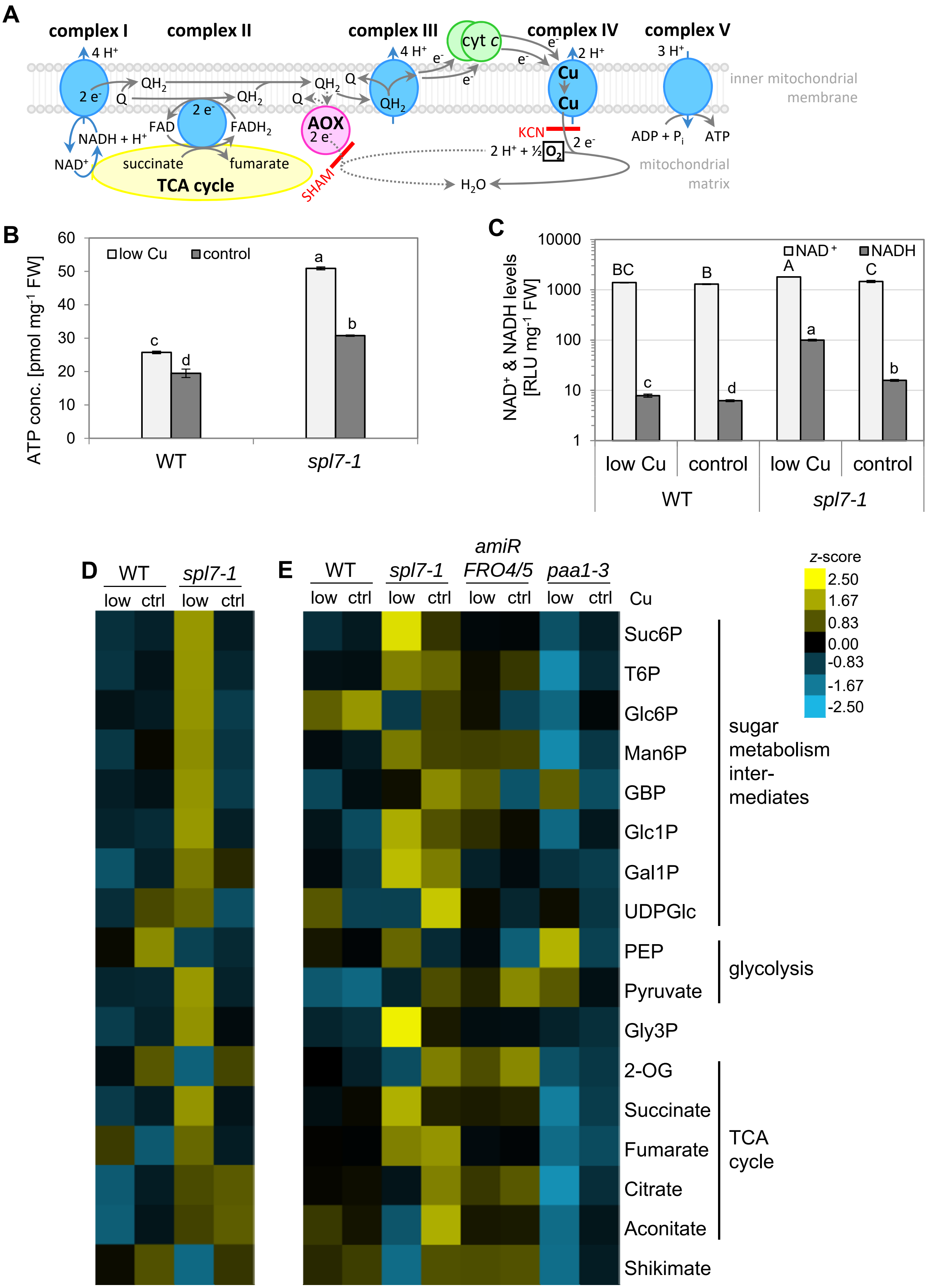
A
complex I
complex II
complex III
cyt c
complex IV
complex V
4 H+
2 e-
QH2
Q
FAD
FADH2
NADH + H+
NAD+
succinate
fumarate
TCA cycle
AOX
SHAM
KCN
Cu
2 H+ + ½ O2
H2O
3 H+
ADP + Pi
ATP
inner mitochondrial membrane
mitochondrial matrix
B
low Cu
control
ATP conc. [pmol mg-1 FW]
WT
spl7-1
C
NAD+
NADH
NAD+ & NADH levels [RLU mg-1 FW]
BC
B
A
C
a
b
c
d
D
E
amiR FRO4/5
paa1-3
low
ctrl
Cu
z-score
2.50
1.67
0.83
0.00
-0.83
-1.67
-2.50
Suc6P
T6P
Glc6P
Man6P
GBP
Glc1P
Gal1P
UDPGlc
PEP
Pyruvate
Gly3P
2-OG
Succinate
Fumarate
Citrate
Aconitate
Shikimate
sugar metabolism inter-mediates
glycolysis
TCA cycle

Supplemental Figure S6. Mitochondrial electron transfer chain and independent experiments (repeats) related to Figure 2.
(A) Schematic diagram of mitochondrial complex I to complex V carrying out oxidative phosphorylation. TCA: tricarboxylic acid; Q: ubiquionone; cyt *c*: cytochrome *c*; AOX: ALTERNATIVE OXIDASE; SHAM: salicylhydroxamic acid; KCN: potassium cyanide; Cu: the two Cu binding sites of cytochrome *c* oxidase (COX, complex IV). Oxygen (O_2) is written in bold font and boxed in black. **(B, C)** ATP (**B**), NAD^+ and NADH levels (**C**) in shoots of 21-d-old seedlings (wild type, *spl7-1*) cultivated in vertically oriented glass petri plates on low-Cu (no $CuSO_4$ added) or control (0.5 µM $CuSO_4$) EDTA-washed agar solid medium supplemented with 1% (w/v) sucrose. Data are means ± SD (n = 3 technical replicates). Different lowercase letters denote significant differences between means based on ANOVA (Tukey's HSD; $P < 0.05$) for (B) and based on *t*-tests with FDR adjustment (q-value < 0.05) for (C), with uppercase and lowercase letters corresponding to different statistical test groups.
(D, E) Heatmap representation of the levels of metabolic intermediates of respiratory and sugar metabolism in shoots. Data are from 21-d-old seedlings (wild type, *spl7-1*, amiR-*FRO4/5*, *paa1-3*) cultivated for 14 d in vertically-oriented glass petri plates on low-Cu (0.05 µM $CuSO_4$) or control (0.5 µM $CuSO_4$) EDTA-washed agar solid medium lacking added sucrose. Represented are *z*-scores (n = 4 and 6 replicate plates per genotype and condition in D and E, respectively. Suc6P: sucrose 6-phosphate; T6P: trehalose 6-phosphate; Glc6P: glucose 6-phosphate; Man6P: mannose 6-phosphate; GBP: glucose 1,6-bisphosphate; Glc1P: glucose 1-phosphate; Gal1P: galactose 1-phosphate; UDPGlc: uridine diphosphate glucose; PEP: phosphoenolpyruvate; Gly3P: glycerol 3-phosphate; 2-OG: 2-oxoglutarate.
(related to Figure 2).

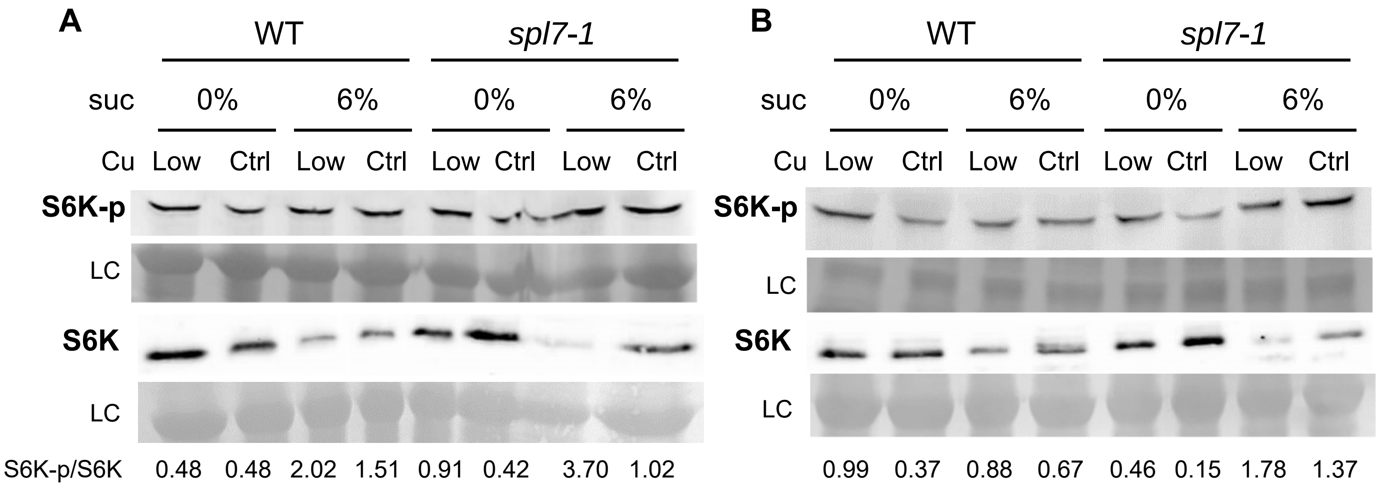

Supplemental Figure S7. Two independent experiments (repeats) related to Figure 4.
(A, B) Immunodetection of S6K protein (S6K-p, S6K; apparent sizes: 52 kDa) in shoots. Data are from 21-d-old wild-type (WT) and *spl7-1* seedlings cultivated for 14 d in vertically-oriented glass petri plates on low-Cu (0.05 µM $CuSO_4$) or control (0.5 µM $CuSO_4$) EDTA-washed agar solid medium with no added sucrose (0%) or supplemented with 6% (w/v) sucrose. Total protein extracts were separated by SDS-PAGE and transferred to nitrocellulose membranes. Proteins were visualized on the membrane through Ponceau S staining prior to immunodetection (loading control, LC). The ratio of S6K-p/S6K band intensities is shown below each lane.
(related to Figure 4).

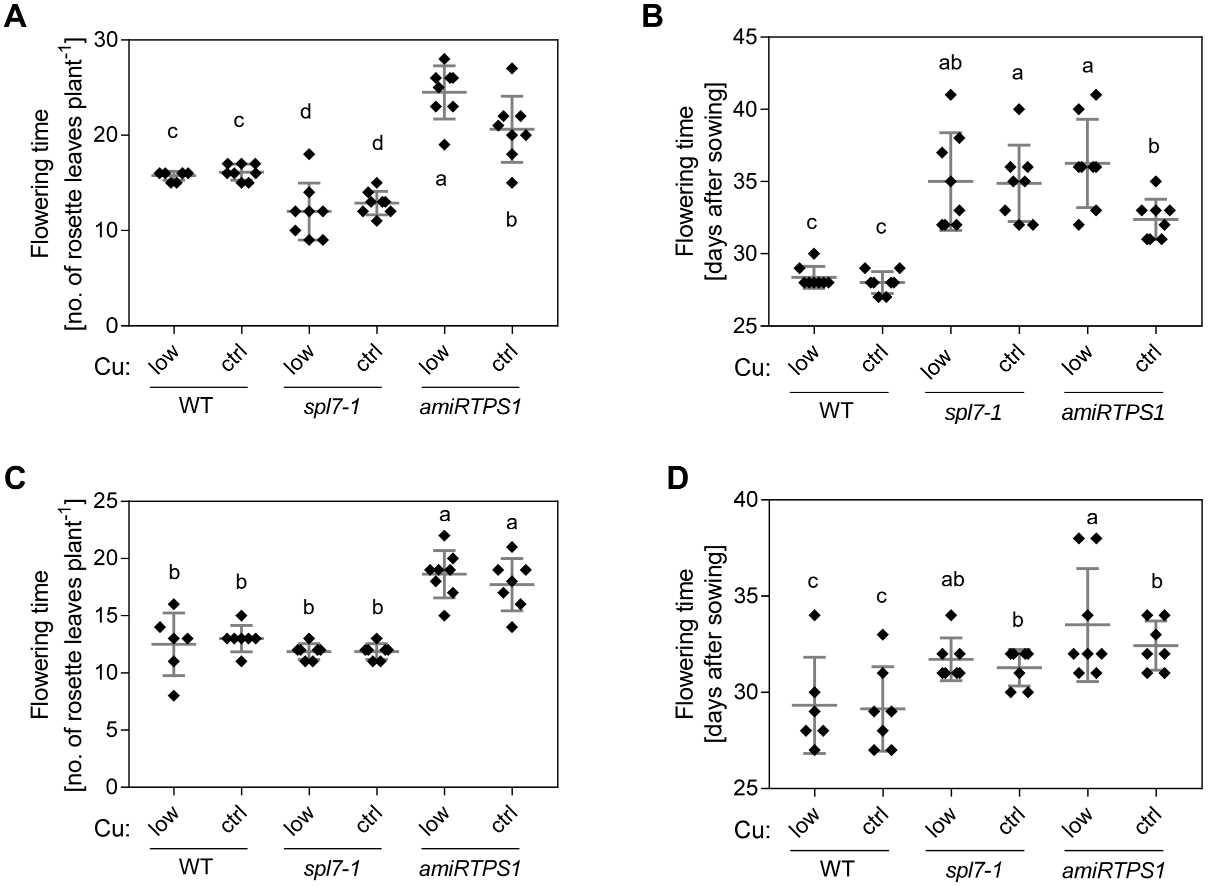

Supplemental Figure S8. Two independent experiments (repeats) related to Figure 5, D and E).

(A-D) Flowering time given as the number of rosette leaves (**A, C**) and plant age (**B, D**) at bolting time of plants cultivated in long days, for two independent experiments (A and B, C and D). Plants were watered with equal amounts of tap water without (low Cu) or with 2 mM $CuSO_4$ (control) once per week. Data are means (line) ± SD, and all individual data points are shown as diamonds (*n* = 8 plants per genotype and treatment).
(related to Figure 5).

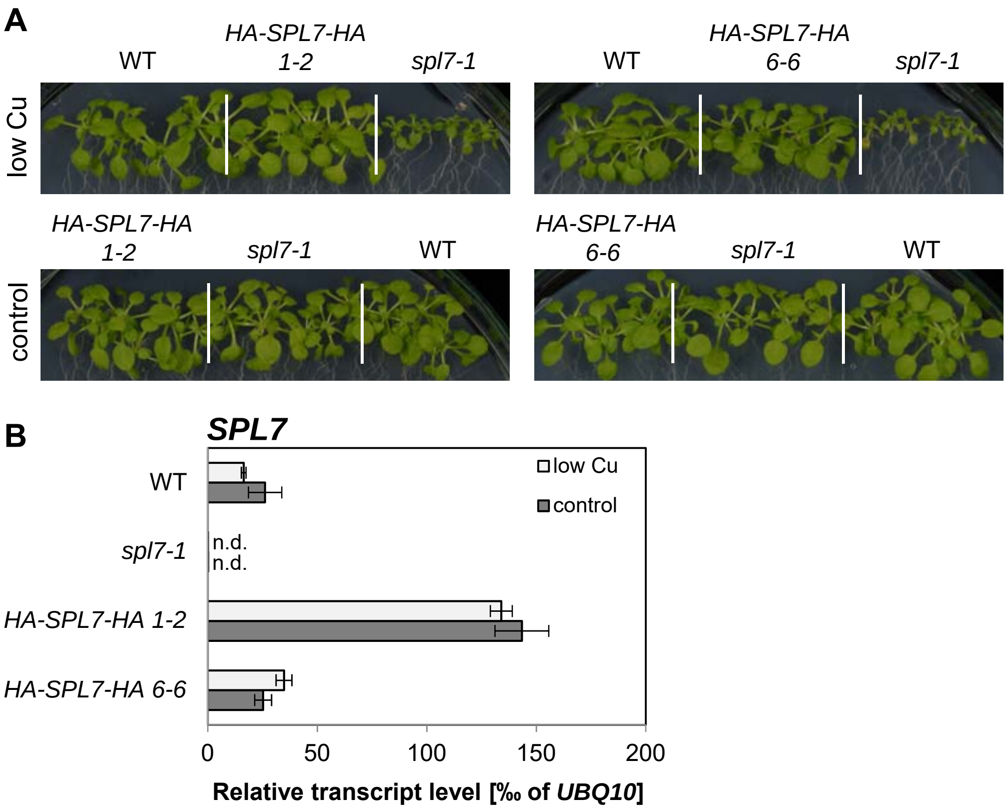

Supplemental Figure S9. Complementation of the *spl7-1* mutant by the *SPL7prom:HA-SPL7-HA:SPL7term* transgene (two independent lines in addition to line 4-1 shown in Figure 6).
(A) Photographs of the wild type (WT), the *spl7-1* mutant and the transgenic homozygous *spl7-1 SPL7prom:HA-SPL7-HA:SPL7term* (*HA-SPL7-HA*) lines 1-2 and 6-6. Shown are 21-d-old seedlings cultivated in low-Cu (no $CuSO_4$ added) or control (0.5 µM $CuSO_4$) medium supplemented with 1% (w/v) sucrose and solidified with EDTA-washed agar in vertically-oriented glass petri plates. **(B)** Relative transcript abundance of *SPL7*, determined by RT-qPCR, in shoots of seedlings cultivated as described in (A). Bars represent arithmetic means ± SD (*n* = 3 technical replicates, i.e. independent PCR runs, each with three replicate wells per transcript). (related to Figure 6).

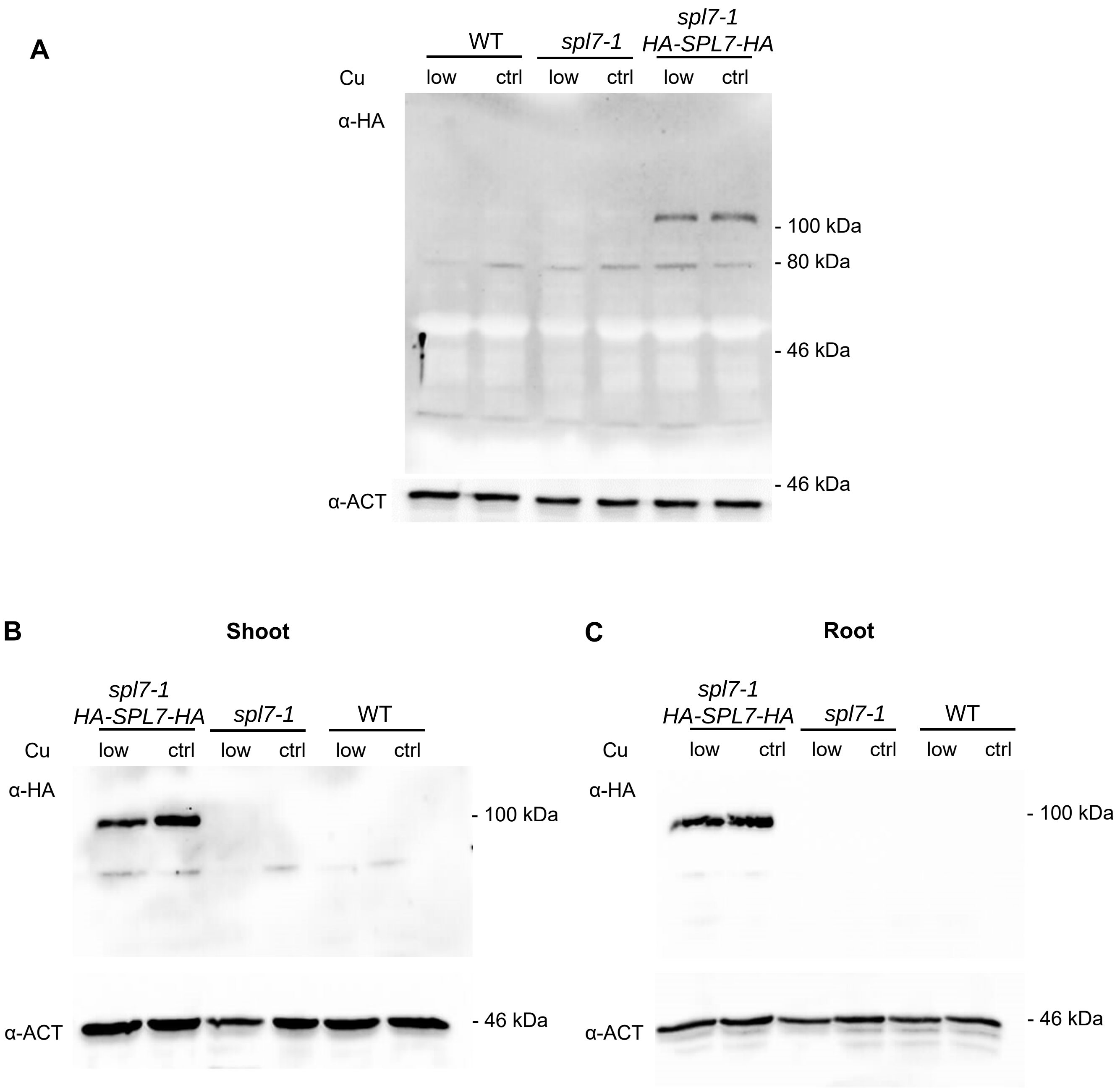

Supplemental Figure S10. Full image and independent replication of immunoblot shown in Figure 6C.
(A) Full image of immunoblot shown in Figure 6C. **(B, C)** Independent experiment with immunoblots of total protein extracts of shoots (**B**) and roots (**C**).
(related to Figure 6).

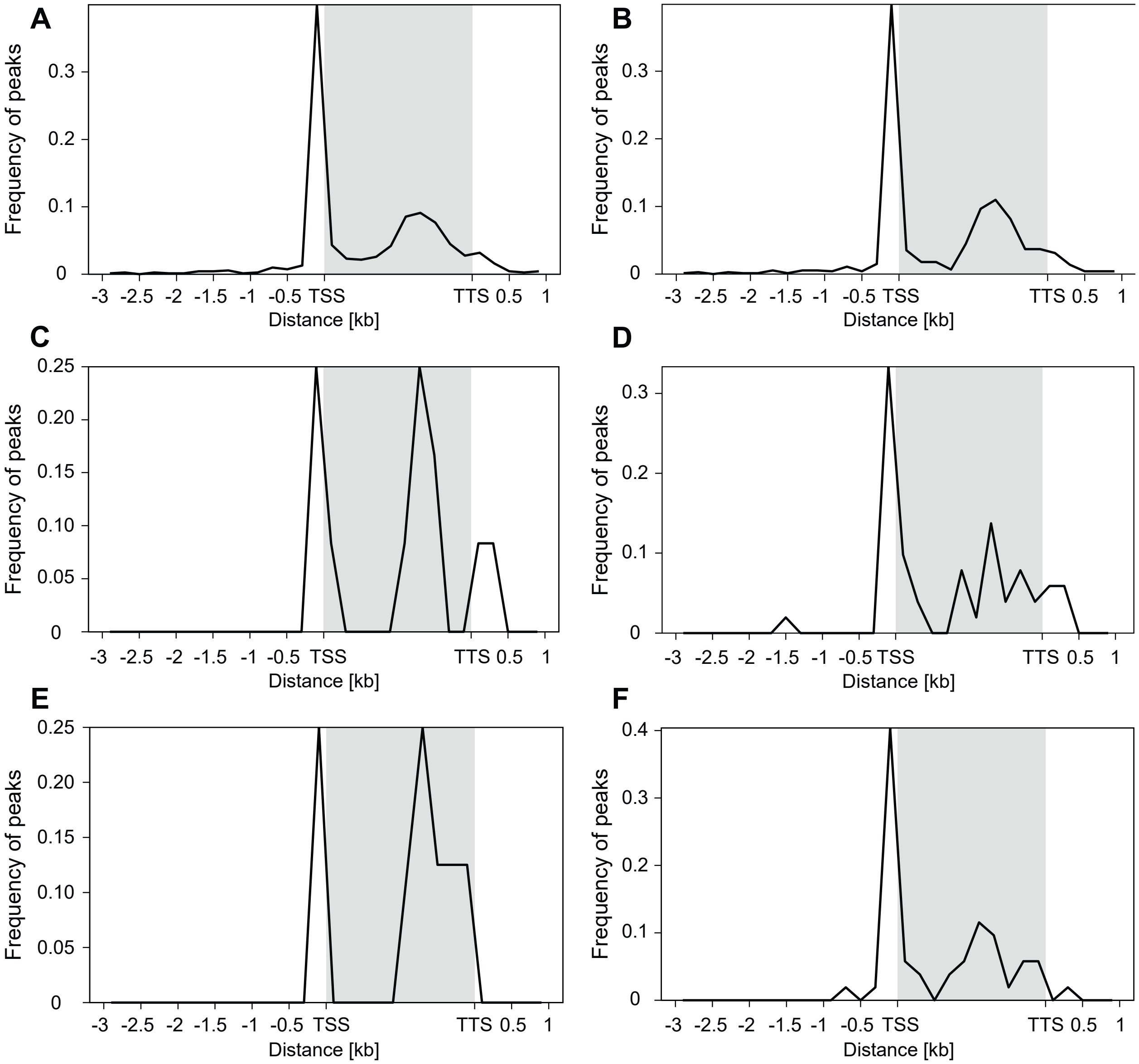

Supplemental Figure S11. Distribution of genomic SPL7 binding sites relative to genes.
(A-F) Positional distribution is shown as the frequency of peak centers (mid-points) of peaks detected by SPL7 ChIP-seq across a region from 3 kb upstream of transcriptional start sites (TSS) to 1 kb downstream of transcriptional termination sites (TTS) of genes (200-bp bin size). Data are shown in subgroups of peaks detected in the shoots of seedlings cultivated under control Cu (**A, C, E**) or low-Cu (**B, D, F**) conditions (see Figure 7), with all peaks **(A, B)**, the subset of peaks for genes transcriptionally activated **(C, D)**, and the subset of peaks of genes transcriptionally repressed dependent on SPL7 under the respective condition **(E, F)**. Diagrams are based on 713 (A), 758 (B), 12 (C), 51 (D), 8 (E) and 52 (F) peaks (see Supplemental Data Set S5 for the formation of groups of genes/peaks). The gene body (gray box) of all corresponding genes was normalized to 2,000 bp. TSS, transcriptional start site; TTS, transcriptional termination site (positions from *A. thaliana* TAIR10 genome annotation).
(related to Figure 7).

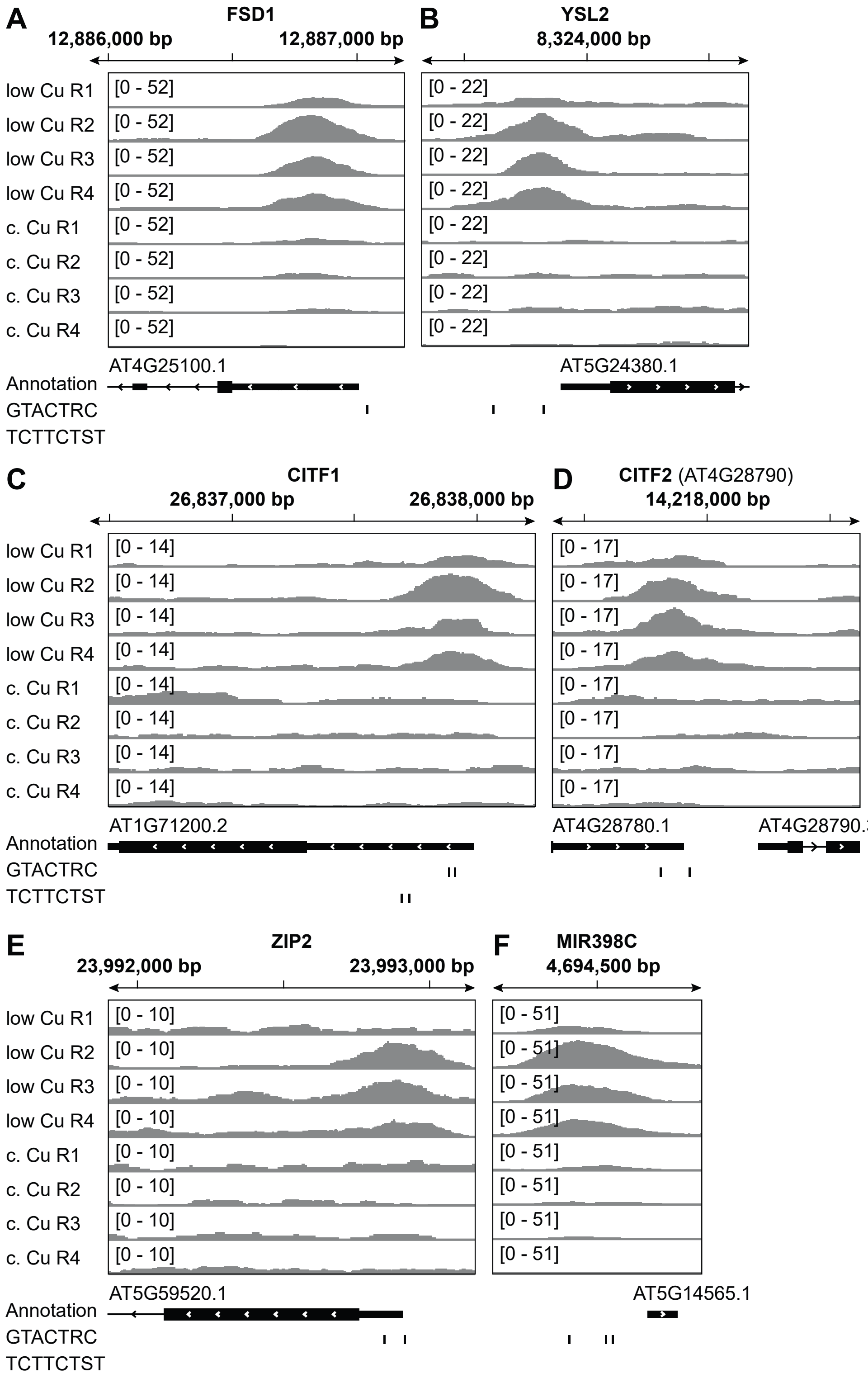
A
FSD1
12,886,000 bp
12,887,000 bp
B
YSL2
8,324,000 bp
low Cu R1
low Cu R2
low Cu R3
low Cu R4
c. Cu R1
c. Cu R2
c. Cu R3
c. Cu R4
[0 - 52]
[0 - 22]
AT4G25100.1
AT5G24380.1
Annotation
GTACTRC
TCTTCTST
C
CITF1
26,837,000 bp
26,838,000 bp
D
CITF2 (AT4G28790)
14,218,000 bp
[0 - 14]
[0 - 17]
AT1G71200.2
AT4G28780.1
AT4G28790.3
E
ZIP2
23,992,000 bp
23,993,000 bp
F
MIR398C
4,694,500 bp
[0 - 10]
[0 - 51]
AT5G59520.1
AT5G14565.1

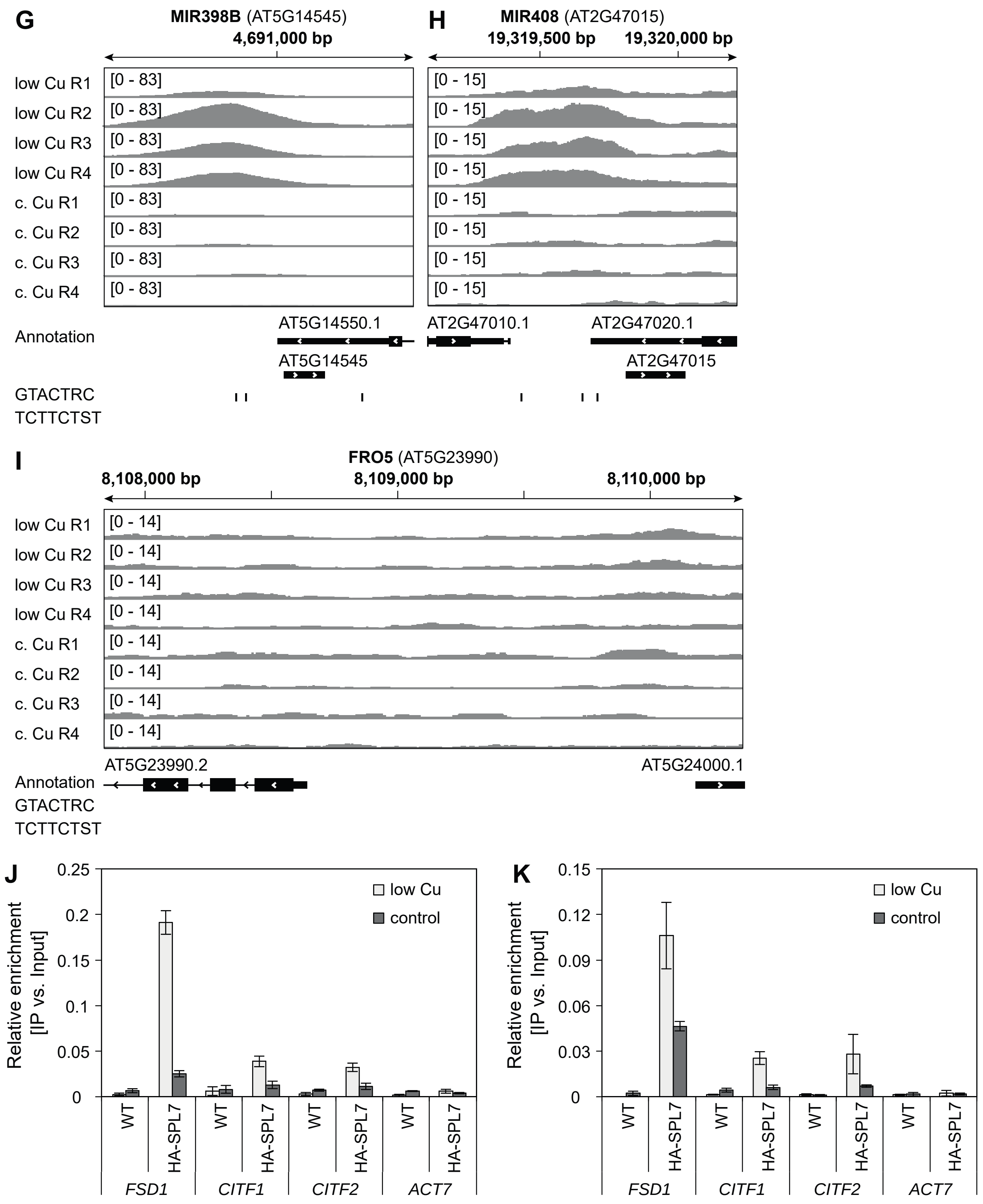
G
MIR398B (AT5G14545)
4,691,000 bp
H
MIR408 (AT2G47015)
19,319,500 bp
19,320,000 bp
low Cu R1
low Cu R2
low Cu R3
low Cu R4
c. Cu R1
c. Cu R2
c. Cu R3
c. Cu R4
[0 - 83]
[0 - 15]
Annotation
AT5G14550.1
AT5G14545
AT2G47010.1
AT2G47020.1
AT2G47015
GTACTRC
TCTTCTST
I
FRO5 (AT5G23990)
8,108,000 bp
8,109,000 bp
8,110,000 bp
[0 - 14]
AT5G23990.2
AT5G24000.1
J
K
Relative enrichment [IP vs. Input]
0.25
0.2
0.15
0.1
0.05
0
0.12
0.09
0.06
0.03
low Cu
control
WT
HA-SPL7
FSD1
CITF1
CITF2
ACT7

Supplemental Figure S12. SPL7 binding profiles at chosen loci, and two independent repeats related to Figure 7C.
(A-I) SPL7 binding profiles based on ChIP-seq peaks visualized using IGV (Integrated genome viewer) at the *FSD1* (**A**), *YSL2* (**B**), *CITF1* (**C**), *CITF2* (**D**), *ZIP2* (**E**), *MIR398C* (**F**), *MIR398B* (**G**), *MIR408* (**H**) and *FRO5* (**I**) loci. *FRO5* is shown as a negative example of a previously reported SPL7-dependently regulated gene that does not appear to be a direct target of SPL7 according to our ChIP-seq data. Numbers [0 … x] on the vertical axis indicate scaling of the shown coverage by sequencing reads. Diagrams are based on the *A. thaliana* Araport 11 genome annotation. Vertical lines below the gene annotations indicate the positions of consensus motifs identified here. c.: control. Rn: replicate number.
(J, K) Validation of ChIP-seq data using ChIP-qPCR for two additional independent experiments (see Figure 7A, C). Data are means ± SD (*n* = 3 technical replicates on a PCR plate) of relative DNA enrichment of the promoter regions of novel candidates *bHLH23* (*CITF2*), *CITF1*, *FSD1*, with *ACT7* (*ACTIN*) as a negative control. Chromatin was independently isolated from another replicate pool of shoots of *spl7-1*, *SPL7prom:HA-SPL7-HA:SPL7term* (*HA-SPL7-HA*) and wild-type seedlings (WT, negative control), generating independent immunoprecipitation (IP) and input samples. Input samples represent aliquots taken after chromatin shearing and before the addition of α-HA for the IP.
(related to Figure 7).

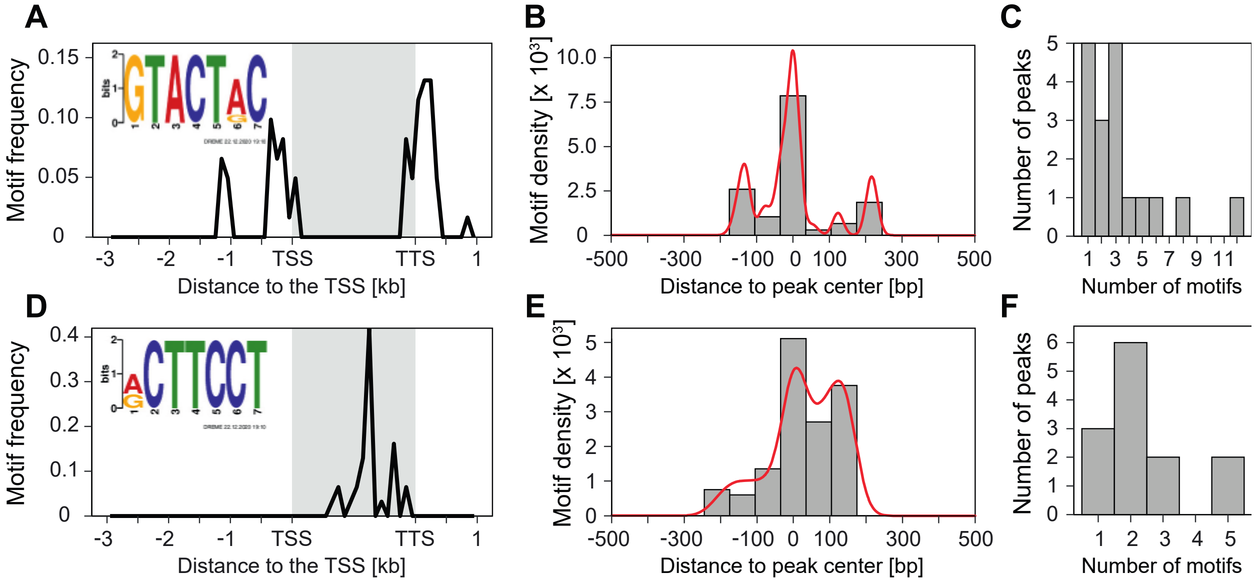

Supplemental Figure S13. Enriched motifs identified by MEME motif analysis among SPL7 binding sites detected exclusively under low Cu.
(A-C) The GTACTRC motif (E-value = 2.1 x 10^{-05}, 23 motif sites, Supplemental Data Set S4). (**D-F)** The RCTTCCT (AGGAAGY) motif (E-value = 2.9 x 10^{-03}, 13 motif sites, Supplemental Data Set S4). Motif frequency positional distributions (100 bp bin size), with conservation logos generated by MEME as an inset (**A, D**). Density plot summarizing distances of motif from center of peaks from ChIP-seq (75-bp bin size) (**B, E**). The red line visualizes the shape of the distribution. Number of motif copies within single peaks (**C, F**).
The gene body (gray box) of all corresponding genes was normalized to 2,000 bp (A, D). TSS: transcriptional start site, TTS: transcriptional termination site (positions from *A. thaliana* TAIR10 genome annotation).
(related to Figure 7).

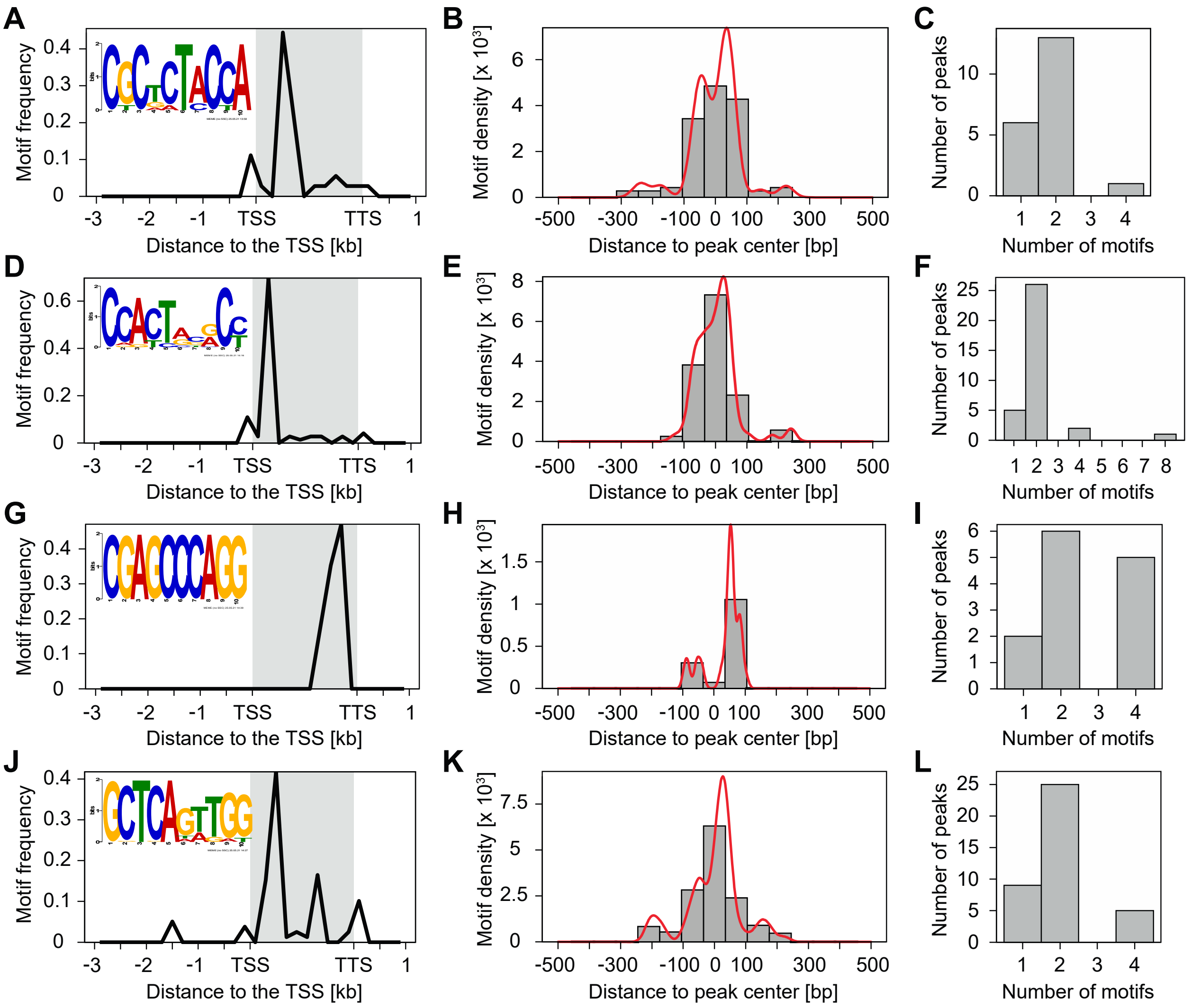

Supplemental Figure S14. Enriched motifs identified by MEME motif analysis on subgroups of SPL7 binding sites delineated by including information on gene expression.
(A-C) The CGCTCTACCA motif (E-value = 1.9 x 10^{-18}, 36 motif sites, Supplemental Data Set S6, iv).
(D-F) The CCACTRSRCY motif (E-value = 1.4 x 10^{-22}, 71 motif sites, Supplemental Data Set S6, vi).
(G-I) The CGAGCCCAGG motif (E-value = 2.5 x 10^{-07}, 34 motif sites, Supplemental Data Set S6, vii).
(J-L) The GCTCAGTTGG motif (E-value = 3.1 x 10^{-61}, 76 motif sites, Supplemental Data Set S6, v).
Motif frequency positional distributions (200-bp bin size), with conservation logos generated by MEME as an inset (**A, D, G, J**). Density plot summarizing distances of motif from center of peaks from ChIPseq (75-bp bin size) (**B, E, H, K**). The red line visualizes the shape of the distribution. Number of motif copies within single peaks (**C, F, I, L**).
The gene body (gray box) of all corresponding genes was normalized to 2,000 bp (A, D, G, J). TSS: transcriptional start site, TTS: transcriptional termination site (positions from *A. thaliana* TAIR10 genome annotation).
(related to Figure 7).

A

```
>mergedPeak_405; associated FSD1, Chr 2: 12886050 - 12887092
12887092 GTTTGAGATAATAAGTGAAAAGAAAACACAAGATAATAAATTATAATTTATAAATGCTTTATAGT
12887027 ATTGAAAAATAAGATGATTTTTTTTTTTTTTAATACCGGATTGGCTGATCCACTTATGATGACTC
12886962 AAATGTTATTAAGTTTCAAGACAATTTATGATGACACAAATCACAATGAGTCAATAGTAGCCACG
12886897 AAGCCAGAAAAAAAAAATGTACTACAAAAAGATAATGATAGTACAAAATGATACGTCGTACTGCC
12886832 ACATGTACGACACAACTCGATTACCAAAAAGCAGAGCCATCCAACCATAAAACTCAAAACACACA
12886767 GATTCCACTGGCGTGTGCTCTCCTCACTTCACTCGTCCTTGAAACTTGAGGTACTGATTCTATCT
12886702 CTCATCCACATCTTAGTTCATGTTCGAGTTTCTCTGGTCTCTTACTCTTTCACCTAGAAATTTGT
12886637 TTGGTCTCCCAACAACATAAACGCATGTATACATTTTCATGTGTCTTTGTAAGGATATTGATTCA
12886572 TTTTTTTGGGCAAATAGATAATCAATTCAATGCTATGTTTTGTCTGAAATGTGCAGCTTCAATTA
12886507 CAAAGAATGGCTGCTTCAAGTGCTGTCACCGCAAACTACGTCCTCAAGCCACCTCCATTCGCACT
12886442 GGTACTTTCTTATGATAAAGATTGATATTGTCGGCAAATGTCATTTTGTTTTCGTTGGACTATAG
12886377 ATTTTGCTTTTCTATTACTAGATGATATAACTTGTTGCTCCCACAAAGCCCTAAAAATATCTTTT
12886312 CATCCTCGTCAAAGGCGTTACAAATGATTTTCAGCTATTATTAACGGAAAACCAATACCAAAAAG
12886247 GCGTTACAAATGAACTCCTCTAATTCATATTTGAAGAAAATAAAAATCGAATCAATCTACGCATT
12886182 AGTGTTTTTCCCGGAAATTAATTGTAGGAACGGGTCAAGTTGGGTGGTCCATATTGGGTAAAGGC
12886117 CGTGAATGTTCTAGTTGGTACGTACTATCCGACATTTGTCAAATGGGAAATTTGGGTATGGCCAT
12886052 AGG
```

GTAC motif

motif flanking-nucleotides

TSS approximated from RNA-seq data of this study

TSS AT4G25100.1 (TAIR)

B

>1_FSD1
AAT**GTAC**TAC
>2_FSD1
ATA**GTAC**AAA
>3_FSD1
GTC**GTAC**TGC
>4_FSD1
CAT**GTAC**GAC
>5_FSD1
GAG**GTAC**TGA
>6_FSD1
CTG**GTAC**TTT
>7_8_FSD1
TTG**GTACGTAC**TAT

C

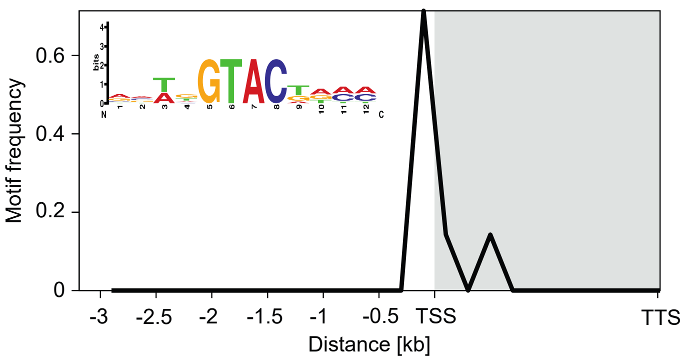

Supplemental Figure S15. Putative SPL7-binding motifs at the *FE SUPEROXIDE DISMUTASE1* (*FSD1*) locus identified by ChIP-seq.

(A) Genomic sequence corresponding to the region spanned by the peak associated with the *FSD1* locus (At4g25100). The header indicates the peak name, its association and the genomic position. The sequence is given as the reverse complement because *FSD1* is encoded on the negative (–) DNA strand. Nucleotides from the beginning of the coding sequence to the end of first exon are underlined. Note that five variants of the transcript are annotated at TAIR, each with a different position of the transcriptional start site (TSS).

(B) List of all core GTAC core motifs (bold) and three flanking nucleotides upstream and downstream (regular) within the *FSD1*-associated ChIP-seq peak.

(C) Motif frequency positional distribution (200-bp bin size), with conservation logo generated by WebLogo (https://weblogo.berkeley.edu/logo.cgi) as an inset, for all *FSD1*-associated GTAC-containing motifs (see A, B). TSS, transcriptional start site approximated from RNA-seq data of this study; TTS transcriptional termination site from *A. thaliana* TAIR10 genome annotation. The gene body (gray box) of *FSD1* was normalized to 2,000 bp.
(related to Figure 7).

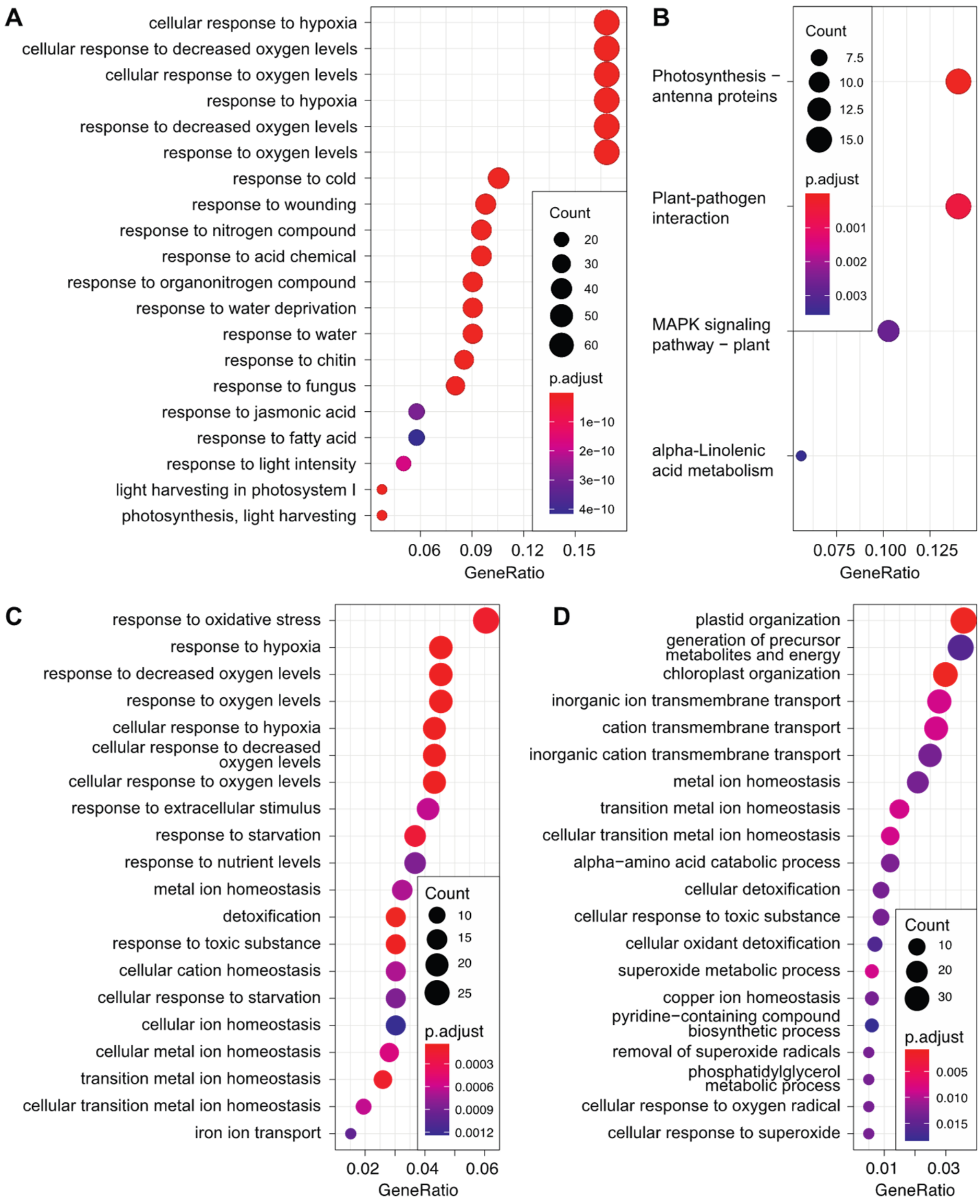
A
cellular response to hypoxia
cellular response to decreased oxygen levels
cellular response to oxygen levels
response to hypoxia
response to decreased oxygen levels
response to oxygen levels
response to cold
response to wounding
response to nitrogen compound
response to acid chemical
response to organonitrogen compound
response to water deprivation
response to water
response to chitin
response to fungus
response to jasmonic acid
response to fatty acid
response to light intensity
light harvesting in photosystem I
photosynthesis, light harvesting
Count
20
30
40
50
60
p.adjust
1e−10
2e−10
3e−10
4e−10
0.06
0.09
0.12
0.15
GeneRatio
B
Photosynthesis − antenna proteins
Plant-pathogen interaction
MAPK signaling pathway − plant
alpha-Linolenic acid metabolism
Count
7.5
10.0
12.5
15.0
p.adjust
0.001
0.002
0.003
0.075
0.100
0.125
GeneRatio
C
response to oxidative stress
response to hypoxia
response to decreased oxygen levels
response to oxygen levels
cellular response to hypoxia
cellular response to decreased oxygen levels
cellular response to oxygen levels
response to extracellular stimulus
response to starvation
response to nutrient levels
metal ion homeostasis
detoxification
response to toxic substance
cellular cation homeostasis
cellular response to starvation
cellular ion homeostasis
cellular metal ion homeostasis
transition metal ion homeostasis
cellular transition metal ion homeostasis
iron ion transport
Count
10
15
20
25
p.adjust
0.0003
0.0006
0.0009
0.0012
0.02
0.04
0.06
GeneRatio
D
plastid organization
generation of precursor metabolites and energy
chloroplast organization
inorganic ion transmembrane transport
cation transmembrane transport
inorganic cation transmembrane transport
metal ion homeostasis
transition metal ion homeostasis
cellular transition metal ion homeostasis
alpha−amino acid catabolic process
cellular detoxification
cellular response to toxic substance
cellular oxidant detoxification
superoxide metabolic process
copper ion homeostasis
pyridine−containing compound biosynthetic process
removal of superoxide radicals
phosphatidylglycerol metabolic process
cellular response to oxygen radical
cellular response to superoxide
Count
10
20
30
p.adjust
0.005
0.010
0.015
0.01
0.03
GeneRatio

Supplemental Figure S16. GO and KEGG enrichment analyses.
(A, B) Gene Ontology (GO) terms (**A**) and Kyoto Encyclopedia of Genes and Genomes (KEGG) pathways (**B**) enriched among genes associated with ChIP-seq peaks observed under any condition in this study (3,927 genes).
(C, D) GO terms enriched among genes exhibiting SPL7-dependent regulation of transcript levels under control (**C**, 599 genes) and low-Cu (**D**, 1,264 genes) conditions. KEGG pathways phenylpropanoid biosynthesis (P_{adj} < 0.01; 15 genes), starch and sucrose metabolism (P_{adj} < 0.01; 13 genes), cyanoamino acid metabolism (P_{adj} < 0.01; 10 genes), MAPK signaling pathway (P_{adj} < 0.05; 10 genes), nitrogen metabolism (P_{adj} < 0.01; 7 genes) and carotenoid biosynthesis (P_{adj} < 0.01; 5 genes) were enriched in the gene set analyzed in (C) (not shown). No KEGG pathway was enriched in the gene set analysed in (D). KEGG and GO enrichment analyses were done using the R package "clusterProfiler" (version 4.0.5) and the organism database "org.At.tair.db" (version 3.13.0). adj: adjusted (Benjamini-Hochberg multiple testing).
(related to Figure 7).
