# Supplementary material for: Energy status-promoted growth and development of Arabidopsis require copper deficiency response transcriptional regulator SPL7
Source: Plant Cell. 2022 Jul 22;34(10):3873–98. doi: 10.1093/plcell/koac215 (PMC9516184; doi:10.1093/plcell/koac215)
Supplement: koac215_Supplementary_Data [file TPC2021RA00796R2 Peer Review Report.pdf › koac215_Supplementary_Data/TPC2021RA00796R2 Peer Review Report.pdf]

Energy status-promoted growth and development of Arabidopsis require copper deficiency response transcriptional regulator SPL7

Anna Schulten, Björn Pietzenuk, Julia Quintana, Marleen Scholle, Regina Feil, Marcus Krause, Maida Romera-Branchat, Vanessa Wahl, Edouard Severing, George Coupland, Ute Krämer

Corresponding author: *Ute Krämer* *Ute.Kraemer@ruhr-uni-bochum.de*

Review timeline:

**TPC2021-RA-00796**	Submission received:	Sep. 17, 2021
	1st Decision:	Nov. 05, 2021 *Request revisions*
**TPC2021-RA-00796R1**	Revision received:	Apr. 11, 2022
	2nd Decision:	May 13, 2022 *Accept (minor revisions)*
**TPC2021-RA-00796R2**	Revision received:	May 31, 2022
	3rd Decision:	Jun. 05, 2022 *Accept*
	Final acceptance:	Jul. 19, 2022

REPORT: (The report shows the major requests for revision and author responses. Minor comments for revision and miscellaneous correspondence are not included. The original format may not be reflected in this compilation, but the reviewer comments and author responses are not edited, except to correct minor typographical or spelling errors that could be a source of ambiguity.)

**TPC2021-RA-00796**	**Submission received**	**Sep. 17, 2021**

**TPC2021-RA-00796**	**1st Editorial Decision – *Request revisions***	**Nov. 05, 2021**

We have received reviews of your manuscript entitled "Energy status-promoted growth and development of Arabidopsis require copper deficiency response transcriptional regulator SPL7." Thank you for submitting your best work to The Plant Cell. The editorial board agrees that the work you describe is substantive, falls within the scope of the journal, and may become acceptable for publication, pending revision and potential re-review.

We ask you to pay attention to the following points in preparing your revision:

1. Please consider carefully the comments of reviewer #1 regarding the use of single spl7 allele (spl7-1) and respond with additional documentation.

2. Please consider comments regarding the need for additional controls.

3. Reviewer #2 has made several comments regarding interpretation of results that warrant consideration and response.

**TPC2021-RA-00796R1**	**1st Revision received**	**Apr. 11, 2022**

-- Reviewer comments (with **Authors responses in** blue, and changes in the manuscript in green):

Dear editors and reviewers,

We thank you for considering our manuscript TPC2021-RA-00796 entitled "Energy status-promoted growth and development of Arabidopsis require copper deficiency response transcriptional regulator

SPL7" and for the positive feedback and constructive comments from the reviewers. Please find our point-by-point responses below. Note that all line numbers refer to positions in the first submission.

Editor's comments

1. **Editor:** Please consider carefully the comments of reviewer #1 regarding the use of single *spl7* allele (*spl7-1*) and respond with additional documentation.

Our response: We conducted additional experiments in order to expand the data provided on a second *spl7* allele. Because of the reproductive defects of *spl7* mutants, while working on this manuscript we had unfortunately lost *spl7-2*, but we have recovered it lately.

Changes made: See reviewer #1, 1.

2. **Editor:** Please consider comments regarding the need for additional controls.

Our response: We considered them and made the requested changes.

Changes made: See reviewer #1, comment 3.

3. **Editor:** Reviewer #2 has made several comments regarding interpretation of results that warrant consideration and response.

Changes made: We have modified the text in the manuscript where necessary in order to argue our decisions or provide more explanation or background information. Reviewer #2, 1., 3.-7., 9., 10.

Reviewer #1

1. **Reviewer's comment:** … I am afraid that I have some issues with the replication strategy for some of the experiments. Most experiments in this work were performed with two independent replicates (which is fine), but using a single mutant allele (*spl7-1*). The Plant Cell guidelines recommend to either use two independent mutant lines or a complemented line in experiments facilitating mutants to support any major conclusions. The use of a **single allele** for most experiments is in particular puzzling to me as the authors are working with/have access to an independent mutant line *spl7-2* and a complemented line *spl7-2 C9* (shown in supplemental Figure 3). I am wondering why experiments were not repeated with the independent mutant line *spl7-2* instead of repeating the experiment with the same allele, *spl7-1*? It would make parts of the manuscript much more convincing to me.

Our response: We believe that both independently repeating experiments with the same mutant genotype and confirming results employing a different genotype with a lesion in the same gene are relevant. Please note that our experiments require extremely laborious accurate medium preparation for Cu deficiency, which limits the size of each single experiment, i.e. the number of genotype-condition combinations. We had thus judged Suppl. Fig. 3A to be sufficient for confirmation in a second allele of *spl7* and a complemented line prior to first submission. During the revision, we have now conducted additional experiments to also include the second *spl7-2* allele and the complemented line to independently reproduce additional central results reported in our manuscript.

Changes made:

- We confirmed the inability of sucrose to stimulate growth (now added in Suppl. Fig. 2)
- We confirmed the starch accumulation (now added in Suppl. Fig. 5)
- We confirmed elevated levels of T6P (new Suppl. Table 2, Experiment 4)

… as reported in *spl7-1* also in *spl7-2*, as well as their rescue in the complemented *spl7-2* C line. The texts of the corresponding legends were adjusted as appropriate. Methods were expanded as appropriate. The results text was adjusted as appropriate (lines 130 and 131, 163-164, 168, 260-261).

2. **Reviewer's comment:** The authors note that SPL7 mRNA abundance is unaltered in response to different external copper concentrations and that HA-SPL7-HA protein abundance (which is driven by the endogenous *SPL7* promoter) is unchanged between low and control copper. It is stated that this data indicates posttranslational regulation. Do the authors assume that SPL7 trafficking is changed upon copper starvation?

Our response: Our intention was not to specifically assume or imply a change in SPL7 trafficking upon Cu deficiency, although we are aware that this has been suggested in the literature (see lines 620-631 of first submission). Instead, we were hoping to leave this statement (lines 373-375) fully open because our data do not provide any information on this. A published model (Kropat *et al.*, 2005; Sommer et al., 2010) that we cite here suggests that binding of Cu to the SPL7 protein interferes with the ability of SPL7 to bind to GTAC motifs in DNA. We would also consider this to be a post-translational mechanism (among a variety of other possibilities). Our data do not confirm proteolytic processing of HA-SPL7-HA (Figure 6C)(see lines 620-631). Our ChIP-seq data also suggest that SPL7 can bind to nuclear DNA under +Cu (control) conditions (we are discussing this result in relation to the ChIP-seq data and the literature in lines 620-631). The Kropat-Sommer model, or possibly a modified version of this model, could in principle be enough to explain Cu status-dependent regulation of SPL7 activity even without having to assume SPL7 trafficking. We attempted subcellular fractionation and also immunolocalization to address the intracellular localization of HA-SPL7-HA. Using the former approach, within the time frame available, we could not obtain sufficient resolution between ER and the nucleus to be able to either demonstrate or exclude SPL7 trafficking. Using the latter approach (immunogold and immunofluorescence), we could not obtain sufficiently strong signals for a localization.

Changes made: We modified the sentence in line 621 of the first submission by inserting "at least", to read "… implying that at least small amounts of SPL7 are located inside the nucleus and active…". At the end of this paragraph (line 631), we clarified by adding: "Our data do not provide any evidence in support of SPL7 trafficking."

3. **Reviewer's comment:** Chromatin-Immunoprecipitation (ChIP) was performed on HA-SPL7-HA lines as well as wild-type lines. ChIP-Seq using an antibody targeting a (tagged) transcription factor is in general a very challenging thing to do, as the expected amount of ChIP-ed DNA is often very low. The ChIP-qPCR data shown in Figure 6D looks very convincing but I urge the authors to include the replication strategy in the figure legend. In addition, I have some concerns regarding the ChIP-Seq study. One control that was included was Input genomic DNA. This is great and will allow to correct for false positive peaks due to genomic DNA properties itself. Based on the information provided, it is not clear to me how ChIP on wild-type cells was used to correct for unspecific cross-reaction of the HA-antibody (to exclude false

positives). It is also not clear to me what R1/2/3/4 refers to in this case, what does four independent experiments mean? Is it independent ChIPs done on the same material four times? Or ChIP done on independent *HA-SPL7-HA* lines? I would recommend that the authors elaborate on this, as one is a necessary control and the other will significantly affect data interpretation/significance.

Our response: We agree with the reviewer that the experiment was challenging, especially in the way we did it, i.e., expressing *HA-SPL7-HA* under the control of the endogenous *SPL7* promoter and not using a highly active ubiquitously expressed promoter (as was done previously).

• Replication: For ChIP-seq, each of the independent experiments denoted as R 1, R 2, R 3, and R 4 are from one independent plant cultivation (i.e., four independent cultivations), with independent ChIP for each of them. In each experiment, we pooled shoots of 80 seedlings per sample, i.e. we pooled from 4 plates per sample with 20 seedlings cultivated per plate, and we generated three pools (samples) per genotype and treatment from each independent experiment. The legend of Figure 7 states this.

• We sequenced input and ChIP. For each independent experiment (= independent plant cultivation), chromatin was immunoprecipitated once for sequencing. For those three experiments for which we carried out a ChIP-qPCR validation, we conducted an independent chromatin immunoprecipitation on a second pool of shoots harvested in the same experiment. The qPCR shown in Fig. 6D was conducted after the chromatin immunoprecipitation for R 1, and the remainder of the DNA was subsequently processed for sequencing.

• We agree with the reviewer that conducting ChIP on the wild type (WT) can reveal nonspecific cross-reaction of the anti-HA antibody with DNA-bound proteins. In each of the ChIP-qPCRs (Figure 6D, 7C, Suppl. Fig. 12J,K), we included the ChIP of the wild type as a control. To save costs, although WT were included in ChIP, we only sequenced WT ChIP once from a single experiment for both – Cu and for + Cu, respectively. We processed WT ChIP data exactly like *HA-SPL7-HA* ChIP data. After correction using the corresponding input data, the remaining coverage in peak regions was only very low (10-13% of raw coverage overall). In addition, peak shapes in the data from those two WT ChIP-seq samples were conspicuous, with frequent high single-read stacks and with the called peaks covering considerably shorter DNA segments than *HA-SPL7-HA* ChIP-seq data, suggesting a comparably high occurrence of PCR amplification artefacts in the WT ChIP-seq data. Our interpretation of these observations was that very small amounts of DNA were immunoprecipitated in wild-type samples and that this affected the preparation of libraries for sequencing. Consequently, we felt that WT ChIP-seq data could be misleading: When there was an overlap, it was often several very short peaks of WT ChIP-seq with one broader peak of *HA-SPL7-HA* ChIP-seq, with the maxima of coverage at distinct positions. We also felt that a true SPL7 binding site could in theory be close to the binding site of a protein that non-specifically cross-reacts with the anti-HA antibody, and consequently the peaks could be identified as overlapping, especially using our rather generous approach in detecting overlaps between peaks (see Methods). To avoid generating false negatives, we decided not to include the WT ChIP-seq data in our data analysis pipeline.

• We used one *spl7-1 HA-SPL7-HA* line for all ChIP experiments, which we chose because its *SPL7* transcript levels were comparable to (but in no case lower than) those in the wild type (Fig. 6B). In other transgenic *spl7 HA-SPL7-HA* lines we had obtained, *SPL7* transcript levels were similar to (sometimes a bit lower) or considerably higher than in the wild type (Suppl. Fig. 9B).

Changes made:

• We have carefully revised the legend texts and hope that we have now fully clarified replication in Fig. 6, 7C and Suppl. Fig 12J,K.
• We clarified plant growth and pooling in the Methods (line 873).
• We also clarified replication in the results text, line 387. We are now providing information on which of the peaks/genes detected with *HA-SPL7-HA* ChIP-seq under both low Cu and control Cu conditions overlapped with any peaks detected in wild-type ChIP-seq (one for low Cu, one for control Cu).
• We have now added the information on peaks and associated genes of the wild type ChIP-seq in Supplemental Data Set 2, and we added a corresponding statement in the results section (line 388) and methodological information in the methods (line 947).

Reviewer #2

1. **Reviewer's comment:** Fig. 2 A to C and a corresponding discussion section.

To me, the authors' conclusion that the respiratory flux was not affected in the *spl7* mutant needs further clarification, especially as it seems that alternative oxidase operates, at least in the *spl7* mutant under low copper conditions.

(**a**) Another concern is that if AOX is involved under these conditions, **why the ATP level is increased**? As far as I am aware, the AOX pathway will not yield ATP because the AOX pathway is not associated with the generation of proton gradients required for ATP synthesis. Therefore, the overall ATP level is expected to be reduced even if complex I in ETC (NADH -dependent) is involved because AOX bypasses two other points in ETC, engaged in the generation of proton gradients.

(**b**) Also, given the essential role of copper in the COX complex activity, it is surprising that the mitochondrial respiration rates (as inferred by oxygen consumption) do not change in the wild type under low copper. It is even more surprising that the *spl7* mutant, which is somewhat copper deficient in control conditions and more so under low copper, is not defective in the COX complex activity/respiration and ATP production). It is unexpected because COX activity requires copper. The requirement for copper for COX activity has been substantiated recently by finding that **copper deficiency significantly reduces the COX complex activity in leaves and flowers of A. thaliana** (https://doi.org/10.1002/pld3.288). This discrepancy merits discussion, and overall, this section needs further clarification. I think COX activity under these conditions should be evaluated to avoid possible artifacts.

(**c**) I would also like to note that it has been recently emphasized that **using AOX inhibitors do not provide a good mean for measuring AOX activity**: AOX capacity can be measured by a sequential addition of KCN (to inhibit COX) and then SHAM, not only by using SHAM; however, the preferred method is by using the oxygen stable isotope fractionation to avoid underestimation of AOX involvement. (doi: 10.1104/pp.110.1.1 and https://doi.org/10.1034/j.1399-3054.2002.1160201; DOI: 10.1007/978-1-4939-7292-0_17).

Our response: Please note that in the abstract, in the results, and the discussion, we concluded more specifically that "in the *spl7-1* mutant, respiratory electron flux *via* Cu-dependent cytochrome *c* oxidase remained unaffected under both normal and low-Cu conditions" (lines 5-7). We agree

that it is an insightful result, which was in accordance with our finding that ATP levels - as an independently measured parameter – were not decreased, but instead elevated in *spl7-1* compared to the wild type (Figure 2, Supplemental Figure 6). As a further finding of less importance for our conclusions, our data additionally suggested that the capacity for AOX-dependent O_2 consumption was enhanced in *spl7-1* cultivated in low Cu. To our understanding, this latter result is consistent with the former two findings. Our understanding from the literature, e.g. Day *et al*. (1996) cited by the reviewer, is that an overly reduced state, in particular, results in enhanced electron flux through AOX. Such an overly reduced state can result from a variety of causes, and it is not necessarily only observed when COX activity is decreased.
(**a**) While the ATP yield from the AOX pathway is lower compared to the (non-AOX) full pathway *via* COX, **some ATP can still be produced *via* the proton-pumping complex I** (Vanlerberghe 2013) (see new Suppl. Fig. 6A). In our manuscript, more importantly, we observed that the respiratory flux via **COX was not decreased** in *spl7* and also not decreased in the wild type cultivated under the low-Cu conditions used in our study – we infer that here the **COX pathway remains able to generate the full extent of the proton gradient required for ATP production**. See also our answers (**b**, **c**) below.

(b) We interpret our findings to suggest that **elevated AOX activity in *spl7* mutants under low Cu is a consequence of an overly reduced state and excess ATP levels** in the mutant and reflects homeostatic regulation in these plants in an attempt of counteracting this imbalance (lines 206 to 212, 506 to 209 of first submission).

Please note that the hypothesis that the *spl7* mutant is largely equivalent to a severely Cu-limited wild type, which we admit appears very straightforward at first glance, is not necessarily true, and our data clearly indicate otherwise (e.g. also Fig. 2D, Suppl. Fig 6D, E).

We agree that COX activity measurements would be very interesting, and we had considered this thoroughly: Unfortunately, the purification of mitochondria for such measurements usually requires very large amounts of tissue (as cited by the reviewer, Del-Saz *et al*. 2017) because of the low yield of clean mitochondria. Because of the very low biomass of *spl7* under our Cu deficiency conditions, we are sorry that were unable to generate enough tissue to perform such a confirmatory experiment.

We hypothesize in the discussion that "It is possible that *spl7-1* seedlings can only maintain adequate Cu supply to COX because of their attenuated growth" (lines 499 to 500), which provides a possible explanation of our finding. Please note also that tissue ATP levels reflect the net outcome of ATP production and ATP consumption.

In the publication cited by the reviewer (Rahmati Ishka and Vatamaniuk (2020), which reports reduced COX activity in the wild type under Cu deficiency conditions, the authors seem to have generated very severe Cu deficiency (i.e. Cu limitation that decreased the growth of the wild type; for more on this see also 5. below). By contrast, in our low-Cu conditions, growth of the wild type was not decreased, i.e. there was Cu deficiency but no Cu limitation in the wild type. This difference between the two studies could very well explain why we saw no difference in COX-dependent respiration in WT seedlings under our Cu deficiency conditions. For a more in-depth comparison with the mentioned study, we felt unable to relate the normalization in the published study ([units ml^{-1}], Rahmati Ishka and Vatamaniuk, 2020) to biomass (used for normalization in our study),

because the concentration of mitochondria per mL volume and the relationship between this and the biomass of used plant tissues were inaccessible to us.

The plants in which COX activity was analyzed in the cited study (Rahmati Ishka and Vatamaniuk (2020) were in the reproductive stage, at which stage floral tissues and likely the reproductive organs themselves are prioritized in Cu allocation (see Figure 5A), in contrast to our experiments conducted for Figure 2, in which plants were at the vegetative stage. This may also contribute to the differences between the findings of these two studies. Please note also that a number of studies, pioneered by the Merchant lab, have repeatedly demonstrated striking and fascinating cases of prioritization of Cu allocation to essential Cu-requiring pathways. So we do not consider our results to be counter-intuitive.

(**c**) We were aware of the limitation of our use of the SHAM inhibitor to measure AOX activity rather than AOX capacity, and have therefore reported our results as an "increased capacity for KCN-insensitive respiration" (lines 192-193). Based on the additional observation that AOX transcript levels are increased in the *spl7* mutant under low Cu (which may explain why we see the increase in KCN-insensitive respiration in *spl7* under low Cu in this experiment), we infer that this likely reflects higher AOX activity (lines 193-197).

If we understand correctly, the main reservation raised in Day et al. (1996) cited by the reviewer, was that the activity of the AOX pathway capacity can be underestimated (**i**) in the absence of any inhibitor or through analyzing the degree of inhibition of AOX-dependent respiration by SHAM. (**ii**) The authors also mentioned that the AOX pathway could be activated artificially as a response to the addition of KCN though the resulting elevated $NAD(P)H/NAD(P)^+$ poise and thus cause an overestimate of KCN-insensitive AOX-dependent respiration. These considerations were based on studies using isolated mitochondria and the use of specific conditions in some experiments, e.g. the presence of high levels of pyruvate. Generally, we feel that these concerns (Day et al., 1996) are likely to affect absolute measurements far more strongly than relative differences observed in comparisons between genotypes and treatments as done in this manuscript. In addition, measurements *in planta* are conducted in the present redox poise, whereas measurements in isolated mitochondria *in vitro* could impose conditions that have little relevance *in planta* and may thus give misleading results.

It would follow from Day et al. (1996) that, if this applied also to our measurements in intact leaves, then, in conclusion, (**i**) there would be a possibility that in WT and – to a lesser extent – *spl7* +Cu COX-dependent respiration would be a bit smaller than indicated by the grey (SHAM) bars (with clearly less expected change in *spl7* –Cu plants because they are already in a highly reduced state even before KCN treatment, as we show). This would thus not affect our conclusions. Secondly, it could be that (**ii**) AOX capacity may be less strongly increased in *spl7* –Cu than suggested by our data (KCN), because our data may lead to a larger increase in AOX capacity in the WT (largest) and in *spl7* +Cu (less), than in *spl7* –Cu, for the same reason as given in (**i**).

Changes made:

- (**a**) We added Suppl. Figure 6A to clarify that ATP production remains possible also when AOX is operating.
- We added in the introduction in line 105 in the introduction after "respiratory electron flux" "*via* COX".

- (**b**) We inserted in sentence line 506-509, after "(Dahan et al., 2014)", severely Cu-limited Arabidopsis (Rahmati Ishka and Vatamaniuk, 2020)".

2. **Reviewer's comment:** Line 169-170 and Fig. 1 D, E: The authors conducted experiments in extreme sucrose conditions: either no sucrose (0%) or high sucrose (6%). Such a high sucrose concentration is, actually, repressive for plant development (11553753 DOI: 10.1104/pp.127.1.252).

Our response: We were aware of this generally known fact, but several aspects need to be considered.

• We would like to emphasize that the phenotypes we observed for the *spl7* mutants under high sucrose (6%), such as sugar accumulation and a failure to stimulate an increase in fresh biomass, persisted without sucrose (sugar accumulation, e.g. Fig. 1E) and in the presence of lower sucrose concentrations such as 0.5-3% (biomass, Figure S2).

• We used this high sucrose concentration because we started out with the intention to follow up on/compare directly with the publication Ren and Tang (2012), in which 6% sucrose was used but only one single Cu condition. In the publication cited by the reviewer (Ohto et al. 2001), seedlings were not only cultivated but seeds were also germinated under these high-sugar conditions: The inhibitory effects of high sugar concentrations are particularly well documented in the literature for seed germination and the early stages of seedling development (eg. Laby et al. 2000, 10.1046/j.1365-313x.2000.00833.x; Dekkers et al. 2004, 10.1007/s00425-003-1154-9, …). Therefore, **we did not germinate our seeds on high sucrose medium, but instead transferred them to such media only after a week of pre-growth on 0.5% sucrose-containing medium** (see Methods for details). Evidently, our data indicate that there was a stimulatory (and no inhibitory) effect of sucrose on the growth of the wild type under our cultivation conditions during the exposure period to high sucrose (14 d in total until harvest). We acknowledge that medium composition, light intensity, daylength, all of which differed between Ohto et al. (2001) and our study, may well impact on the degree of plant sucrose tolerance. While in Ohto et al. (2001), the effects on flowering time were observed after several weeks of cultivation on high sucrose medium, their experimental setup makes it difficult to untangle effects on germination from the delay in plant development. We have explicitly explained the transfer in the results text (lines 124-125). Our data clearly show that the sucrose treatment increased growth with no signs of damage in the wild type (photos) (Figure 1A, B). We feel that a discussion of this would be excessive.

3. **Reviewer's comment:** At high sucrose and low copper, the spl7 mutant accumulates soluble sugars and starch; this defect seems to be absent under control conditions /no sucrose in the spl7 mutant (Fig 1D&E and Supplemental Fig. 5A; please note that statistically significant values are not indicated). Therefore, I disagree that spl7 is generally impaired in sugar utilization (Lines 169-180). It seems to be impacted only under low copper conditions.

Our response: Please see our response to 9. The difference is there under control Cu, but to a quantitatively less pronounced extent than under low Cu (compare bars for 0 and 6% sucrose, respectively, between WT control and spl7-1 control in Fig. 1D, E, Suppl. Fig. 3B, C and Suppl. Fig. 5A, B). Compare also T6P levels between WT and spl7 under control Cu (Fig. 2D, E, Fig. 5A, Suppl. Fig 6E).

4. **Reviewer's comment:** … It seems that Cu accumulation is decreased even further in the *spl7* mutant vs. wild type under high sucrose. Please clarify.

Our response: To address this, it is generally preferable to first look at shoot Cu content, i.e. total shoot Cu (Suppl. Fig. 1E) rather than shoot Cu concentration (Suppl. Fig. 1A), because differences in growth can be confounding (see Bernal et al., 2012). We did not observe statistically significant differences for either WT or *spl7* between no sucrose and high sucrose in low-Cu conditions. So this would suggest that Cu is down in *spl7* compared to the wild type to a similar degree under both high- and no-sugar conditions.

When looking at shoot Cu concentrations (although not preferable, see above), they were significantly lower under high-sucrose compared to no sucrose supply in both *spl7* (41% of high sucrose condition) and in the wild type (53% of high sucrose condition).

In conclusion, our data suggest that shoot Cu concentrations are lower in high-sucrose than in low-sucrose media in both the wild type and in *spl7* as the major effect. To our impression, given the levels of variation, our data do not convincingly show that the reduction in shoot Cu concentrations under high sucrose is more severe in *spl7* than in the wild type - this is a minor effect at best. The (major) genotype-independent effect of sucrose might be related to *COPT2* transcript levels, for example (lines 145-147). We were trying not to expand this text too much and not to go into excessive descriptive detail of quantitatively minor differences in order to keep the manuscript to a reasonable length.

Changes made: We corrected some characters in Suppl. Fig. 1A to place them into an ascending order of use from highest to lowest numbers.

5. **Reviewer's comment:** Fig. 5 D: According to OI: 10.1093/jxb/erp173: the increased/decreased number of rosette leaves upon transition to flowering serves as an indicator of the defect in the vegetative to reproductive stage transition. Since the number of rosette leaves in the mutant was similar to that in the wild type (unlike amiR-*TPS1*), it seems that the mutant does not have a defect in transition from the vegetative to reproductive stage. The delayed flowering time of the mutant (Fig. 5E) is an indicator of the slower development. Would you please clarify accordingly? I would also like to note that the authors' finding is opposite to what is shown for the effect of copper deficiency on the transition to flowering https://doi.org/10.1002/pld3.288. Please discuss.

Our response: We agree with the interpretation of the reviewer concerning the vegetative to reproductive stage transition in the *spl7* mutant, and we would like to point out for precision that we conducted this experiment in long (16-h) days. We conclude that based on internal sugar levels, *spl7* would be expected to flower earlier (i.e., with fewer leaves) than the wild type, and *spl7* is thus observed in our work to flower later than expected. In Rahmati Ishka and Vatamaniuk (2020), which the reviewer refers to, the authors reported the effect of severe Cu deficiency on flowering time in the wild type without the inclusion of an *spl7* mutant. The authors observed flowering at an increased age with a larger number of leaves, suggesting a delay of vegetative-to-reproductive stage transition in severely Cu-deficient wild-type plants in 14-h days. It appears that the authors generated a very severe degree of Cu limitation in the wild type, which is possible in hydroponics but not in the soil substrate that we used here. Under the conditions of Rahmati Ishka and Vatamaniuk (2020), the *spl7* mutant would probably not have survived at all.

We feel that the observations we report on the difference between the wild type and *spl7* in Figure 5 suggest that there is more than merely Cu deficiency, or something entirely different from Cu deficiency, which impacts on the *spl7* mutant (Cu supply made no difference although it partially rescued the low Cu levels and the growth defect of *spl7*). We regret that unfortunately we could not identify a suitable reference point for directly comparing our data to the findings reported by Rahmati Ishka and Vatamaniuk (2020) because these authors did not include in their publication leaf Cu levels in the Cu-deficient and Cu-sufficient wild-type plants in which they analyzed flowering time (and there is also no information on sugar levels in this publication). In our study, we observed no alterations in sugar levels in Cu-deficient wild type plants – yet we acknowledge that Cu deficiency was probably far less severe in our work than in Rahmati Ishka and Vatamaniuk (2020). Differences in the experimental design is another reason for our difficulties in comparing our results with the published results. In our study, plants were grown in the same soil throughout, whereas in the published work, if we understand correctly, plants were cultivated in a Cu-containing hydroponic solution for 4 weeks and subsequently transferred to various hydroponic solutions containing Cu concentrations from 0 nM to 500 nM so hat Cu deficiency conditions (0 nM Cu) lasted for approximately 25 d until flowering.

Changes made:

• Abstract; we replaced "Late flowering of *spl7-1*" (line 11) by "Delayed *spl7-1* development"…

• In the discussion (line 560), we inserted after "flowering of *spl7-1* was delayed", "relative to plant age", before "and unaltered by Cu supplementation", although at a given ~~plant~~ age the levels of metabolizeable sugars … were increased.".

• At the end of this paragraph (line 572), we added "Note that a delay in the transition from vegetative to reproductive stage was reported in hydroponically cultivated severely Cu-deficient wild-type Arabidopsis plants (Rahmati Ishka and Vatamaniuk, 2020)."

6. **Reviewer's comment:** Surprisingly, there are no statistically significant differences in the fresh biomass accumulation of the *spl7* mutant vs wild type under low copper and *spl7* under control conditions. The difference is obvious in Fig. 1A. I wonder about the appropriate statistical analysis for this data set, or the number of replicates used to increase the statistical power. Also, please clarify how many independent experiments were used and what technical replicates mean (for example, for Fig. 1C, n=3 technical replicates for one pool; the same is for D and E). Also, please clarify why not all values are characterized statistically in Fig. 1D and E and Supplemental Figures 3, 5 (characters denoting statistically significant differences are not present for all data points).

Our response: We fully agree with the reviewer that the lack of a statistically significant difference in biomass between low-Cu grown wild type and *spl7* mutant plants was counter-intuitive based on the means and error bars (Fig. 1B).

Fig. 1C, D, E: "technical replicates" means that one extract was generated from a pool of shoots collected from one single glass petri plate per genotype and condition (for number of seedlings per plate see line 716) with *n* indicating the number of replicate measurements conducted for each extract. Independent experiments (i.e., independent plant cultivation) is shown in Suppl. Fig. 3A and B-C, respectively.

We are very sorry that in conducting these very laborious experiments that are highly sensitive to Cu contamination, we were limited in the size of one experiment and also by the low biomass obtained for some genotypes/treatments. We did not conduct statistics when $n < 3$ (as a result of insufficient biomass obtained).

Changes made:

• We re-ran the statistics for Fig. 1B using a more appropriate procedure (multiple testing with FDR adjustment instead of Tukey's HSD). This revealed a statistically significant difference. We adjusted diagram 1B accordingly.

• Adjusted legend texts of Fig. 1C-E and Suppl. Fig. 3 to clarify replication and pooling.

7. **Reviewer's comment:** Line 137: "The maintenance of wild type-like anthocyanin levels in *ran1-1* (Woeste and Kieber, 2000) indicated against an ethylene-signaling defect as a cause of this *spl7* mutant phenotype". Please explain the reference to *ran1* and define what is it. Perhaps it is worth mentioning the role of Cu in ethylene perception in the Introduction. Also, if I interpreted the results and text in Woeste and Kieber, 2000 correctly, the *ran1-3* allele used in this manuscript accumulates more anthocyanins than wild-type (fig. 2 in Woeste and Kieber, 2000). Please either explain the statement in lines 137-138, or delete it.

Our response: We regret that this text was overly shortened during our streamlining of the text when we edited the manuscript.

Changes made: We expanded this section to explain in more detail. Woeste and Kieber (2000) is the description of a strong *ran1-3* allele that is rosette-lethal on soil. The authors report anthocyanin accumulation in flowers of a very sick mutant based on a photo, as well as in soil-grown rosette leaves (not visible in the photo/not quantified). Different from Woeste and Kieber, we used the mild *ran1-1* allele identified by Hirayama *et al*. (1999). We modified the results and the citations therein to provide more background information and to specify our expectations as well as to explain previously published results on the relationship between ethylene signaling and sugar-inducible anthocyanin accumulation (expanded lines 137-139 of first submission). In essence, we uphold that the anthocyanin accumulation in *spl7* cannot be explained by altered ethylene signaling resulting from an expected lack of Cu in ethylene receptors of *spl7* (at least when cultivated under Cu deficiency).

8. **Reviewer's comment (other comments):** Given that the authors test the role of SnRK1 and TOR pathways in the *spl7-1* mutant-related phenotypes, I suggest including a brief description of the pathways in the Introduction. I would also suggest including a description of the respiratory pathways and perhaps an image in the supplemental information to help the reader navigate the authors' data.

Our response: We prefer to provide the introduction to TOR and SnRK1 in the results section (as in the first submission) because bringing up these topics in the introduction would seem a little out of context. Moreover, the corresponding results are negative, i.e., both TOR kinase and SnRK1 remain sugar-responsive in *spl7*, which means that they are ultimately not relevant for explaining *spl7* phenotypes.

A similar argument applies for mitochondrial respiration, and therefore we are not expanding on it further in the revised introduction.

Changes made: Following the reviewer's suggestion, we have introduced a new scheme as panel Suppl. Fig. 6A in order to help the readers understand our approaches and interpretations in Fig. 2 and Suppl. Fig. 6. We have introduced a reference to this in the results section (line 178).

9. **Reviewer's comment (other comments):** Lines 117-119: "In summary, maintaining a balance between energy availability, growth and development requires SPL7 function, especially under Cu-deficient growth conditions." I think this is mainly copper-related function of SPL7. Most of the defects in the *spl7* mutant in this manuscript are observed under low copper. Please amend.

Our response: We thought very thoroughly about how to briefly and appropriately reflect our findings when phrasing this summarizing statement at the end of the introduction. We agree with the reviewer that some of the phenotypes are only observed under low-Cu conditions, and that phenotypes are generally more severe in low-Cu conditions. However, very importantly, central results clearly suggest that there is also a (smaller) difference between *spl7* and the wild type in control (+ Cu) conditions (Fig. 1D, E and Suppl. Fig. 3B, C; Fig. 2C and Suppl. Fig. 6B; Suppl. Fig. 5A, B). Even more importantly, some of our results suggest that some central phenotypes, most importantly elevated T6P levels in *spl7* mutants, are independent of Cu supply (Fig. 2E and 5A; Fig. 5D, E and Suppl. Fig. 9B, D; Fig. 2D and Suppl. Fig. 6C, D and Suppl. Table 1 (in revision also 2), see also abstract lines 10 and 11. To reflect this appropriately, we believe that we should leave this summarizing statement (for brevity) here as is.

Changes made: We checked throughout the manuscript whether we clarified this sufficiently in the first submission. We have now made a small modification in the introduction (line 104) (see also 3. above, 10. below).

10. **Reviewer's comments (other comments):** Discussion, starting from line 558 "SPL7 and Cu deficiency in plant development". A recent study examined the role of copper in plant development and reproduction comprehensively and invoked the role of sugars and T6P and FT, and miRs in the vegetative to reproductive stage transition https://doi.org/10.1002/pld3.288. Please discuss as relevant to the data presented in this manuscript.

Our response: See also reviewer #2, comment 5. We did not find any sugar or T6P data in this publication. Sucrose was invoked in the discussion to act upstream of FT. The authors discuss that under severe Cu deficiency, wild-type plants may contain too little copper for sustaining photosynthesis, resulting in lower sucrose levels and reduced FT expression.

Changes made:

- See also reviewer #2, comment 5.

- We changed line 11 in the abstract and lines 108-109 in the introduction accordingly.

- We added additional sentences below this in the discussion (after line 572): "This finding was in accordance with lowered *FT* transcript levels that were attributed to decreased photosynthetic

activity, but in opposition to strongly increased transcript levels of the floral marker *MIR172C* and *D*, in Cu-limited wild-type plants (Rahmati Ishka and Vatamaniuk, 2020). Similar to the Cu-limited wild-type plants examined by the authors, the *spl7* mutants of this study lack Cu, but they appear to be impaired in the utilization of sugar, and not in sucrose production." It seems that the authors analyzed the levels of the primary transcripts and not the levels of the mature processed miRNA.

• In addition, we altered and expanded the text from line 559 to line 562 to read: "Compared to the wild type, flowering of *spl7-1* was delayed relative to plant age and unaltered by Cu supplementation. We observed this despite the fact that at a given age of *spl7-1*, the levels of the metabolizeable sugars sucrose and glucose were increased, and T6P concentrations were similarly elevated irrespective of whether plants were cultivated in low-Cu or Cu-supplemented conditions (Figures 1 and 5, Supplemental Figures 3 and 8)."

We hope that these changes clarify our conclusion that these *spl7* phenotypes are unrelated to Cu limitation, which we expect to occur predominantly in low-Cu conditions.

11. **Reviewer's comment:** Given that CITF1 has been identified to act together with SPL7 in regulating copper homeostasis, please mention it in the Introduction (doi: 10.1105/tpc.17.00363).

Our response: We were trying to keep the introduction short and not introduce excessive complexity here.

Changes made: We moved the description of CITF1 function from the discussion to the introduction and slightly expanded it as necessary in this position of the manuscript (line 98).

12. **Reviewer's comment:** Lines 126-127: "To test for interactions between Cu homeostasis and sugar metabolism, we transferred 7-d-old wild-type and *spl7-1* mutant seedlings onto agar-solidified medium differing in Cu and sucrose contents (Supplemental Figure 1A and E, compare B-D and F) (Bernal et al., 2012; Marschner and Marschner, 2012)". Unsure why this statement needed a reference. Also, the authors point to panels B-D and F but do not state what we need to compare. I would suggest the author to avoid pointing the reader to specific panels unless the authors specify what it is about the data on these panels that need attention.

Our response: We are sorry: This originates from several rounds of aggressive shortening of a previous draft text.

Changes made:

Instead of the text in parentheses in lines 126/127, we inserted a full sentence: "After 14 d, shoot concentrations of Cu (Supplemental Figure 1A and E), when compared with those of Fe, Zn and Mg (Supplemental Figure 1B-D and F), confirmed the implementation of Cu deficiency, and were in agreement with our expectation of generally lowered Cu levels in *spl7-1* (Bernal et al., 2012; Marschner and Marschner, 2012). "

13. **Reviewer's comment:** Lines 149- 151: "We confirmed sucrose responses of transcript levels of known SPL7-dependently regulated genes in the wild type, which were reported previously on media containing identical sucrose concentrations (Ren and Tang, 2012) (Supplemental Figure 4)." I would

suggest the authors explain in the text what the reader expects to see with the transcript levels of SPL7 targets in response to sucrose, since to me this is not obvious. Please do not force the reader to go back to the referenced manuscript to infer what to expect in the current manuscript.

Our response: Sorry, these data are a little difficult to follow- we had thus tried to keep our short, because our central goal here was to analyze whether/which of these previously reported sugar responses were *SPL7*-dependent. And the answer was: none of these were *SPL7*-dependent. We are presenting these results because they explain how we proceeded subsequently, but they are broadly in agreement with previously published data and thus not novel or highly relevant here.

Changes made: We changed the corresponding text to provide the details requested by the reviewer: "Sucrose responses of transcript levels of known *SPL7*-dependently regulated genes were previously reported in wild-type plants cultivated on MS (Murashige and Skoog, 1962) media containing 6% sucrose, for example (Dugas and Bartel, 2008; Ren and Tang, 2012). We confirmed a sucrose-dependent upregulation of *pri-MIR398B* accompanied by a downregulation of miR398 target *CSD2*, albeit only under low-Cu conditions, as well as downregulation of copper transporter(*COPT2*)-encoding and *FSD1* transcript levels (Supplemental Figure 4). Importantly, these sucrose responses…"

14. **Reviewer's comment:** Fig. 3A can be moved to the supplemental information.

Our response: We agree that this would be possible in principle. There are already 16 Supplemental Figures and that this panel would have to become a new independent Supplemental Figure because of the question addressed here and the differing experimental design. Moving Fig. 3A would mean splitting the contents of current Figure 3 into Figure 3 and a new Supplemental Figure. We thus prefer to leave Fig. 3A in its context of Fig. 3B and C.

Changes made: None.

15. **Reviewer's comment:** Fig. 5C: please indicate plant lines shown in this figure.

Our response: They are indicated in the legend text.

Changes made: We inserted "(left to right)" in the legend text to clarify.

16. **Reviewer's comment:** Lines 24-26: Please provide a reference for classifying the transition elements as class "B" elements.

Changes made: Done.

17. **Reviewer's comment:** Lines 39-40: "As a result, Cu cofactors carry out challenging ligand binding tasks, for example, the interaction with gas molecules" - please provide relevant support from the literature.

Our response: Would be Fraústo da Silva and Williams, 2001.

Changes made: We removed this sentence in an effort to shorten the manuscript (see reviewer #3).

Reviewer #3

1. **Reviewer's comment:** … My suggestion for revision is for shortening of sections particularly Introduction, Results and Discussion. e.g. I felt the beginning of the Discussion was more Introductory material. But also, the Introduction and Results sections could be shortened.

Changes made: We shortened the manuscript text.

- We shortened the introduction on Cu in biology and removed or shortened text on metallochaperones and Chlamydomonas (as long as not relevant for any of the results presented).
- We deleted introductory material on plastocyanin, mETC, energy signaling and the miR156/subfamily II SPL module from the discussion, and moved part of the references or fragments of sentences into the introduction.
- We deleted text to remove any redundancies or excessive detail when giving literature background in the results.

TPC2021-RA-00796R1	2nd Editorial decision – *Accept (minor revisions)*	May 13, 2022

We have received reviews of your manuscript entitled "Energy status-promoted growth and development of Arabidopsis require copper deficiency response transcriptional regulator SPL7." On the basis of the advice received, the board of reviewing editors would like to accept your manuscript for publication in The Plant Cell. This acceptance is contingent on revision based on the comments of our reviewers. In particular, please consider the following:

The reviewer asked that you consider adding text to guide the reader to supplementary figures that clarify replication/statistical analyses (see comment below).

TPC2021-RA-00796R2	2nd Revision received	May 31, 2022

-- Reviewer comments (with **Authors responses in** blue):

Dear editors and reviewers,

We thank you for considering our manuscript TPC2021-RA-00796.R1 entitled "Energy status-promoted growth and development of Arabidopsis require copper deficiency response transcriptional regulator SPL7" and for your positive feedback and constructive comments from the reviewers. Please find our point-by-point responses below.

Editor's comments

4. **Editor:** The reviewer asked that you consider adding text to guide the reader to supplementary figures that clarify replication/statistical analyses (see comment below).

Our response: See Reviewer #2, comment no. 2 below.

Reviewer #2

4. **Reviewer's comment:** Overall, the authors satisfactorily addressed my comments.

(a) However, I still would like to come back to my point regarding energy generation in the *spl7* mutant, COX vs. AOX activities, etc. I want to emphasize that the *spl7* mutant **generates more ATP while growing under low copper**. Actually, it seems that this was a severe copper deficiency as the mutant was grown on EDTA-washed agar without added copper). While I agree with the suggestion made by the authors that "*spl7-1* seedlings can only maintain adequate Cu supply to COX because of their attenuated growth." I wonder why a **similar event does not occur in *A. thaliana* wild type grown under severe copper deficiency**. Under severe copper deficiency, ***A. thaliana* wild-type growth is also restricted, but COX activity is much lower (Rahmati Ishka & Vatamaniuk, 2020).**

(b) Also, while I agree that AOX function can preclude overproduction of ETC, usually **high AOX activity is accompanied by reduced ATP levels** (as the authors stated, **some ATP is still produced if the complex I is active and generates proton motive force for the subsequent ATP synthesis**). I do not want to dwell on and look forward to seeing a follow-up study.

Our response:

(a) In the response to reviewer #2, comment 1b with first revision, we detailed a number of reasons why a comparison of our results with those published on the wild type in Rahmati Ishka & Vatamaniuk, (2020) is unfortunately difficult. Note that we provide in our manuscript ATP concentrations, which are a result of the processes of ATP generation and ATP utilization. Our results cannot be interpreted to indicate that *spl7* generates more ATP when grown under low copper.

Please note that the hypothesis that the *spl7* mutant is largely equivalent to a severely Cu-limited wild type, which we admit appears very straightforward at first glance, is not necessarily true. Indeed, our data very clearly show that the *spl7* mutant is phenotypically very different from Cu-deficient wild-type plants (e.g. also Fig. 2A, D, Suppl. Fig 6D, E). Thus, the concluding sentence of the results paragraph "Increased respiration rates and altered profile of respiration-related metabolites in *spl7-1*", reads as follows (first revision): "Together, these results suggest that the primary metabolite profile of *spl7-1* mutants is rather different from that of other Cu-deficient mutants, and thus they implicate *SPL7*-dependent processes, rather than pleiotropic defects common to Cu-limited plants, in reduced growth despite a high energy status of *spl7-1*." We also emphasized in the discussion that "Dissimilar from the properties of COX-defective (Dahan et al., 2014), severely Cu-limited wild-type plants (Rahmati Ishka and Vatamaniuk (2020), and other Cu-deficient genotypes (Figure 2D), we interpret enhanced respiration, increased $NADH/NAD^+$ ratio, accumulation of ATP and an altered metabolite profile of *spl7-1* as consequences of an elevated sugar status, and not as its causes." (first revision).

(b) Please note that in the abstract, in the results, and the discussion, we concluded more specifically that "in the *spl7-1* mutant, respiratory electron flux *via* Cu-dependent cytochrome *c* oxidase remained unaffected under both normal and low-Cu conditions" (lines 4-6 of the abstract). This was in accordance with our finding that ATP levels - as an independently measured parameter – were not decreased, but instead elevated in *spl7-1* compared to the wild type (Figure 2, Supplemental Figure 6). As a further finding of less importance for our conclusions, our data additionally suggested that the capacity for AOX-dependent O_2 consumption was enhanced in *spl7-1* cultivated in low Cu. We did not directly quantify any proxies of *in planta* AOX activity. Our understanding from the literature, e.g. Day *et al.* (1996), is that an overly reduced state, in

particular, results in enhanced electron flux through AOX. An overly reduced state can result from a variety of causes, and it is not necessarily only observed when COX activity is decreased (and based on our respiration measurements, COX activity does not seem to be decreased in *spl7*). We interpret our findings to suggest that **elevated AOX capacity in *spl7* mutants under low Cu is a consequence of an overly reduced state and excess ATP levels** in the mutant and reflects homeostatic regulation in these plants in an attempt of counteracting this imbalance (as stated in results and discussion of first submission and first revision).

Changes made:

To respond to the need for clarifying (a) further,

• we introduced a novel sentence at the end of the introduction "These metabolic features clearly differentiated *spl7* mutants from Cu-deficient wild-type plants, and they were also unlike the metabolic features previously reported in severely Cu-limited wild-type plants (Rahmati Ishka and Vatamaniuk, 2020). "
• we rephrased the corresponding section in the discussion (underlined above), to read: "Enhanced respiration, increased NADH/NAD^+ ratio, accumulation of ATP, and an altered metabolite profile of *spl7-1* are metabolic features dissimilar from the properties of COX-defective plants (Dahan et al., 2014), severely Cu-limited wild-type plants (Rahmati Ishka and Vatamaniuk, 2020), and other Cu-deficient genotypes (Figure 2D). We interpret these metabolic phenotypes of *spl7* mutants as consequences of an elevated sugar status, and not as its causes."

5. **Reviewer's comment:** Concerning my comment about the number of replicates and statistical analysis in Fig. 1 D,E and Supplemental Fig. 3B,C: I would suggest the authors guide the reader in the figure legend for Fig. 1 D, E to the independent experiment in Supplemental Fig. 3B.C. Otherwise, n=2 is not satisfactory.

Our response: To our understanding, we did this in the first revision. We regret very much that we do not understand what else the reviewer wishes us to add beyond what we wrote already in the first revision for guiding the reader:

• Figure legend of Fig. 1D, E reads as follows according to the first revision: "(D, E) Sucrose (D) and glucose (E) concentrations in shoots (see A). Bars represent arithmetic means ± SD (n = 2 and 3 technical replicates for 0% and 6% sucrose samples, respectively). Technical replicates (C-E) are replicate measurements conducted per extract from a pool of 20 or 40 shoots of seedlings cultivated on one plate per genotype and treatment, see Methods). Data from independent experiments (independent plant cultivation) are shown in Supplemental Figure 3 (C-E)."
• Figure Legend of Supplemental Figure 3 (B, C) reads as follows according to the first revision: "Supplemental Figure 3. Independent experiment (repeat) related to Figure 1 (C-E). (A) Anthocyanin levels in shoots of 21-day-old seedlings cultivated in vertically oriented glass petri dishes on low-Cu (0.05 µM CuSO4) or control (0.5 µM CuSO4) agar-solidified media supplemented with 0% or 6% (w/v) sucrose for 14 d. Shown are data for the wild type (WT), *spl7-1*, *spl7-2*, an *spl7-2 SPL7* complemented line (*spl7-2 C*; Bernal et al., 2012), and the *ran1-1* mutant for comparison. Bars represent arithmetic means ± SD (n = 3 technical replicates). (B, C) Sucrose (B) and glucose (C)

concentrations in shoots of wild-type and *spl7-1* cultivated as for (A). Bars represent arithmetic means ± SD (n = 2 and 3 technical replicates for 0% and 6% sucrose condition, respectively). Technical replicates (A-C) are replicate measurements conducted per extract from a pool of 20 or 40 shoots of seedlings cultivated on one plate per genotype and treatment, see Methods). Different characters denote statistically significant differences between means (t-tests with FDR adjustment; q-value < 0.05), wherever n > 2."

• In the results text of the first revision, when Figure 1D-E is referred to the first time, it is done as "(Figure 1D-E, Supplemental Figure 3B-C for an independent experiment)".

TPC2021-RA-00796R2 2nd Editorial decision – *Accept* Jun. 05, 2022

We are pleased to inform you that your paper entitled "Energy status-promoted growth and development of Arabidopsis require copper deficiency response transcriptional regulator SPL7" has been accepted for publication in The Plant Cell, pending a final minor editorial review by journal staff. At this stage, your manuscript will be evaluated by a Science Editor with respect to its presentation of scientific content, compliance with journal policies, and presentation for a broad readership.

Final acceptance from Science Editor Jul. 19, 2022
